# MNK2 deficiency potentiates β-cell regeneration via translational regulation

**DOI:** 10.1038/s41589-022-01047-x

**Published:** 2022-06-13

**Authors:** Christos Karampelias, Kathleen Watt, Charlotte L. Mattsson, Ángel Fernández Ruiz, Habib Rezanejad, Jiarui Mi, Xiaojing Liu, Lianhe Chu, Jason W. Locasale, Gregory S. Korbutt, Meritxell Rovira, Ola Larsson, Olov Andersson

**Affiliations:** 1 Department of Cell and Molecular Biology, Karolinska Institutet, Stockholm, Sweden; 2 Science for Life Laboratory, Department of Oncology-Pathology, Karolinska Institutet, Solna, Sweden; 3 Department of Physiological Science, School of Medicine, University of Barcelona (UB), L’Hospitalet de Llobregat, Barcelona, Spain; 4 Pancreas Regeneration: Pancreatic Progenitors and Their Niche Group, Regenerative Medicine Program, P-CMR[C], Institut d’Investigació Biomèdica de Bellvitge - IDIBELL, L’Hospitalet de Llobregat, Barcelona, Spain; 5 Center for Networked Biomedical Research on Bioengineering, Biomaterials and Nanomedicine (CIBER-BBN), Madrid, Spain; 6 Alberta Diabetes Institute, University of Alberta, Edmonton, Alberta Canada; 7 Department of Pharmacology and Cancer Biology, Duke University School of Medicine, Durham, NC USA; 8 Present Address: Department of Molecular and Structural Biochemistry, NC State University, Raleigh, NC USA

**Keywords:** Translation, Stem cells, Experimental organisms, Target identification, Metabolism

## Abstract

Regenerating pancreatic β-cells is a potential curative approach for diabetes. We previously identified the small molecule CID661578 as a potent inducer of β-cell regeneration, but its target and mechanism of action have remained unknown. We now screened 257 million yeast clones and determined that CID661578 targets MAP kinase-interacting serine/threonine kinase 2 (MNK2), an interaction we genetically validated in vivo. CID661578 increased β-cell neogenesis from ductal cells in zebrafish, neonatal pig islet aggregates and human pancreatic ductal organoids. Mechanistically, we found that CID661578 boosts protein synthesis and regeneration by blocking MNK2 from binding eIF4G in the translation initiation complex at the mRNA cap. Unexpectedly, this blocking activity augmented eIF4E phosphorylation depending on MNK1 and bolstered the interaction between eIF4E and eIF4G, which is necessary for both hypertranslation and β-cell regeneration. Taken together, our findings demonstrate a targetable role of MNK2-controlled translation in β-cell regeneration, a role that warrants further investigation in diabetes.

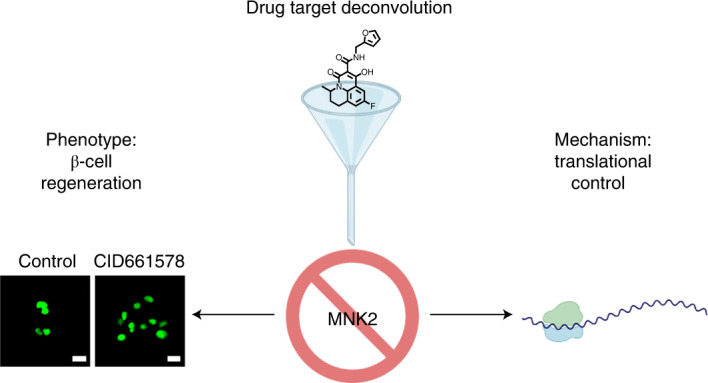

## Abstract (web summary)

MNK2 was identified as the target of a small molecule named CID661578 that can stimulate pancreatic β-cell generation in zebrafish, pig and human organoids. CID661578 prevents MNK2 from binding to eIF4G in the translation initiation complex.

## Main

Both type 1 and type 2 diabetes manifest with elevated circulating glucose levels caused by the deregulation of insulin signaling and/or the loss of functional insulin-producing β-cells^1^. Although daily insulin injection, lifestyle interventions and various drug treatments can manage the disease, there is currently no available cure. Therefore, stimulating endogenous β-cell regeneration is an attractive curative approach for diabetes; however, current efforts have failed to translate into a clinically approved drug.

Drug screening in vivo for chemicals stimulating β-cell regeneration has the potential to accelerate the drug discovery process as it is performed in a physiological, whole-organism setting. The zebrafish model has emerged as a powerful tool for performing unbiased, large-scale chemical/genetic screens that are directly coupled to phenotypic analyses. These types of screens have identified molecules that have already entered clinical development^2,3^, showcasing the translational potential of the approach. Metabolism, and in particular diabetes research, is an area in which chemical screens using zebrafish have grown in popularity. Such screens have already identified compounds that can stimulate β-cell proliferation^4–6^ and neogenesis from duct-residing pancreatic progenitors^7–9^, two of the main known mechanisms of endogenous β-cell regeneration. Moreover, zebrafish chemical screens for regulators of glucose metabolism have identified compounds with potential use as antidiabetic treatments^10–12^. Collectively, these studies have demonstrated the power of the zebrafish model for performing chemical screens to identify compounds that could be repurposed as antidiabetic drugs. However, many hits from phenotypic screens have unknown targets and mechanisms of action that create a bottleneck for further development but, if the targets are defined, can open up new research areas.

In this work, we aimed to identify the molecular mechanism of action of CID661578 (**1**), the most striking hit from a zebrafish chemical screen for stimulators of β-cell regeneration^4^. By performing a modified yeast two-hybrid screen suited for drug target deconvolution, we identified MAP kinase-interacting serine/threonine kinase 2 (MNK2) as the molecular target of CID661578 and validated this interaction in vivo. MNK2 participates in initiation of mRNA translation and has been postulated to modulate the process in a transcript-selective fashion^13^. Here, we show that kinase-independent blocking of MNK2 leads to bolstered protein synthesis in the pancreatic duct and that the effects are conserved across zebrafish, pig and organoid cultures of human pancreatic ducts. Overall, our results demonstrate a conserved pathway to stimulate β-cell neogenesis by boosting protein synthesis through targeting MNK2.

## Results

### Yeast chemical hybrid screen identifies MNK2 as the molecular target of CID661578

**Fig. 1:**
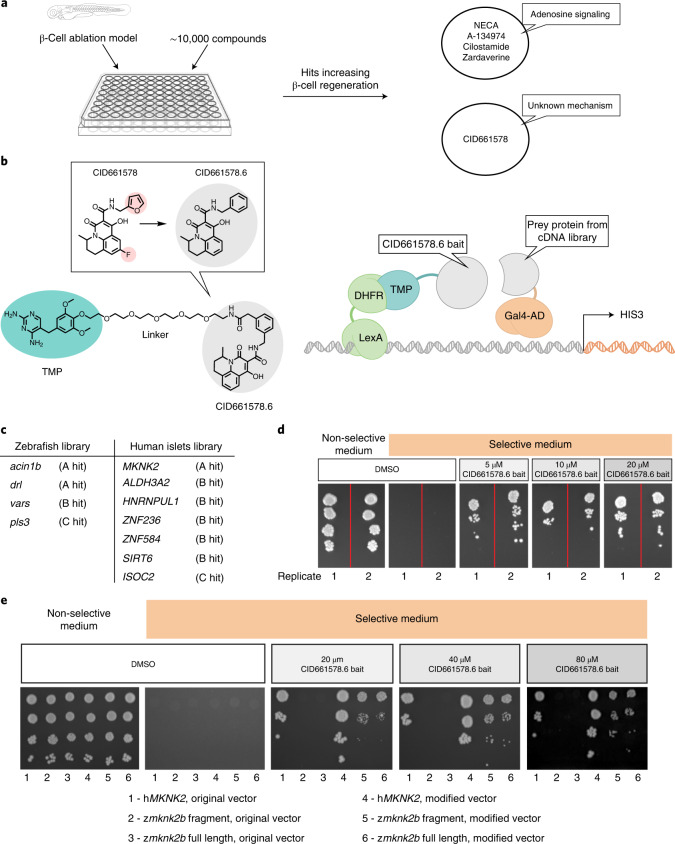
YChemH screen identifies MNK2 as the molecular target of CID661578. **a**, Schema for the screening of compounds increasing β-cell regeneration using a transgenic zebrafish model for β-cell ablation and approximately 10,000 compounds. The hits included four compounds affecting adenosine signaling and CID661578 with an unknown mechanism. **b**, Schematic showing the structures of CID661578 and the analog CID661578.6 along with the screening strategy (YChemH). The red circles highlight the structures that were altered in CID661578. Survival of yeast on selective histidine-free medium was the output of the screen for clones expressing interactors of the CID661578.6 bait; TMP, trimethoprim; AD, activation domain. **c**, Table summarizing the top hits of the YChemH screen from the two cDNA libraries. The A-classified hits (*drl* and *acin1b* from the zebrafish embryo library and *MKNK2* from the human islet library) have a higher probability of being true targets of CID661578.6 than B- and C-classified hits. **d**, Validation of the MNK2–CID661578.6 interaction with different concentrations of CID661578.6 bait and an MNK2-expressing yeast clone. DMSO demonstrates the sensitivity to the selective medium, and yeast clones did not survive in the selective histidine-free medium. The interaction between MNK2 and CID661578.6 promoted yeast survival, as illustrated by the multiple colonies at the four spots of inoculation (decreasing levels of inoculation from the top to the bottom). Each condition was tested in two replicates. **e**, Validation of the zebrafish Mnk2b–CID661578.6 interaction with different concentrations of CID661578.6 bait and two different DHFR hook vectors. Experiments using the original hook vector, N-LexA–DHFR-C, are listed as 1, 2 and 3. Experiments using the modified vector with the reverse order, N-DHFR–LexA-C, are listed as 4, 5 and 6. Both full-length zebrafish Mnk2b (3 and 6) and a fragment (2 and 4) corresponding to the original fragment of the human MNK2 identified in the screen were used. Human MNK2 was used as a positive control (1 and 4), and zebrafish Mnk2b only mediated binding when expressed by the hook vector with the reverse order (5 and 6) to the one used in the original screen (explaining why zebrafish Mnk2b did not show up as a hit in the original screen).

**Extended Data Fig. 1:**
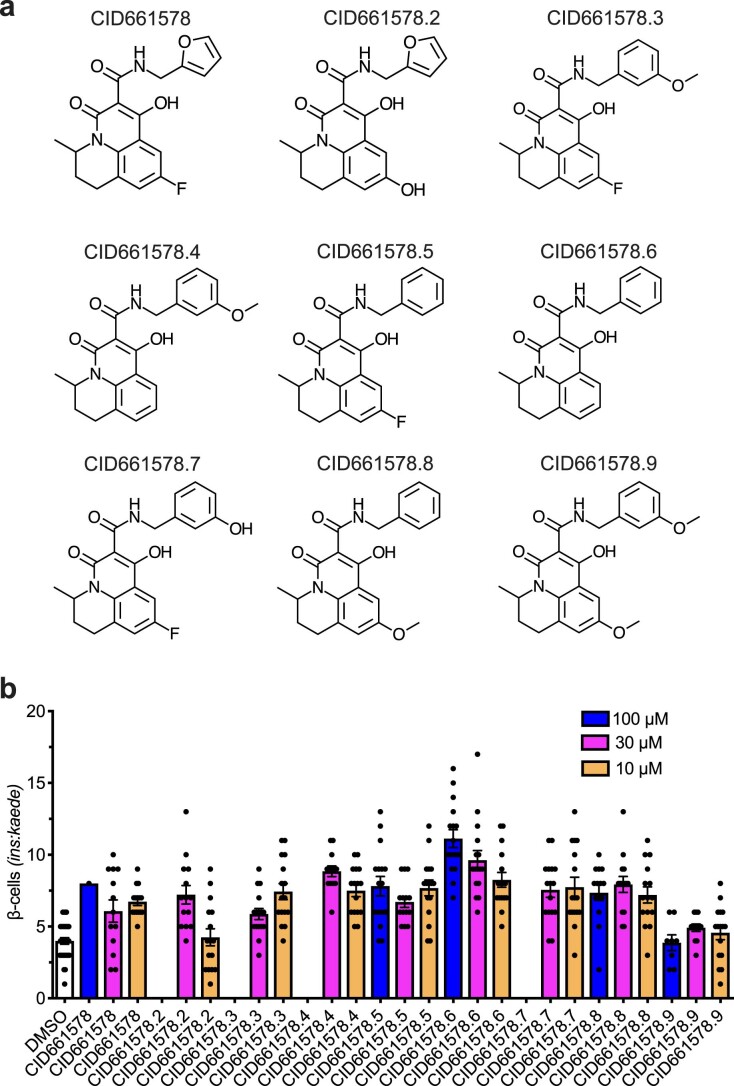
Effect of different analogues of CID661578 on β-cell regeneration. **a**, Chemical structures of the different analogues of CID661578 synthesized for the screen. **b**, Quantification of the number of regenerating β-cells in *Tg(ins:kaede);Tg(ins:CFP-NTR)* treated with the indicated analogue, tested in three different concentrations from 4-6 dpf after β-cell ablation. The absence of bars denote that the chemical treatment was toxic to the larvae. *n* = 32 (DMSO), *n* = 1 (CID661578-100 µM), *n* = 13 (CID661578-30 µM), *n* = 15 (CID661578-10 µM), *n* = 0 (CID661578.2-100 µM), *n* = 14 (CID661578.2-30 µM), *n* = 16 (CID661578.2-10 µM), *n* = 0 (CID661578.3-100 µM), *n* = 16 (CID661578.3-30 µM), *n* = 16 (CID661578.3-10 µM), *n* = 0 (CID661578.4-100 µM), *n* = 13 (CID661578.4-30 µM), *n* = 16 (CID661578.4-10 µM), *n* = 16 (CID661578.5-100 µM), *n* = 14 (CID661578.5-30 µM), *n* = 16 (CID661578.5-10 µM), *n* = 16 (CID661578.6-100 µM), *n* = 16 (CID661578.6-30 µM), *n* = 16 (CID661578.6-10 µM), *n* = 0 (CID661578.7-100 µM), *n* = 16 (CID661578.7-30 µM), *n* = 16 (CID661578.7-10 µM), *n* = 14 (CID661578.8-100 µM), *n* = 14 (CID661578.8-30 µM), *n* = 15 (CID661578.8-10 µM), *n* = 8 (CID661578.9-100 µM), *n* = 12 (CID661578.9-30 µM) and *n* = 16 (CID661578.9-10 µM). Data are presented as mean values ±SEM. Source data

In a previous large-scale chemical screen, we identified five small molecules that potently drove β-cell regeneration in zebrafish larvae^4^. Four of the five hit compounds converged on the adenosine pathway and stimulated β-cell proliferation, while the fifth hit compound, named CID661578, had no known molecular target or cellular mechanism of action (Fig. 1a). To identify the molecular target of CID661578, we used yeast chemical hybrid (YChemH) screening technology, which takes advantage of the classical yeast two-hybrid system for protein–protein interactions but enables screening for protein targets of small molecules. First, we generated a series of structural analogs of CID661578 to simplify the structure and identified one (termed CID661578.6 (**2**)) that exerted similar effects on β-cell regeneration as CID661578 (Extended Data Fig. 1). We used yeast clones with a construct that expressed a DNA-binding domain (LexA) coupled to the enzyme dihydrofolate reductase (DHFR) along with a GAL4 activation domain fused to each cDNA in the libraries. We screened two different cDNA libraries, one derived from human islets and another derived from zebrafish embryos, with a total of 135 million and 122 million clones, respectively. The rationale of this approach is that when yeast are incubated with the CID661578.6-derived bait (**3**), the attached trimethoprim will interact with DHFR on the DNA-binding site of LexA. Interaction between CID661578.6 and a protein fragment (that is, the prey) generated from the cDNA library will lead to yeast survival on the selective histidine-free medium (Fig. 1b). The most likely targets of CID661578 were classified as A hits, and less likely targets were classified as B hits, C hits and so forth, depending on the confidence in the interaction (Fig. 1c).

Next, we followed up on hits that had previously been shown to affect metabolism and could validate the interaction in an MNK2-expressing yeast clone with different concentrations of the CID661578.6-derived bait in the YChemH system (Fig. 1d). Subsequently, we assessed whether the zebrafish homolog of MNK2 (Mnk2b) could also bind CID661578.6 because it did not appear as a hit in the zebrafish cDNA library. To this end, we cloned the cDNA sequence of *mknk2b* (the genes encoding Mnk kinases are called *mknk*) in frame with the GAL4 activation domain and used two different DHFR hook vectors of the YChemH system. The YChemH assay results revealed that zebrafish Mnk2b only bound to CID661578.6 when the new, modified DHFR hook vector was used, while human MNK2 bound to CID661578.6 regardless of the vector conformation (Fig. 1e), explaining why Mnk2b did not show up as a hit in the original screen of the zebrafish cDNA library. In summary, through a series of in vitro experiments, we identified MNK2 as the likely molecular target of CID661578.

### CID661578.6 promotes β-cell regeneration of ductal origin

**Fig. 2:**
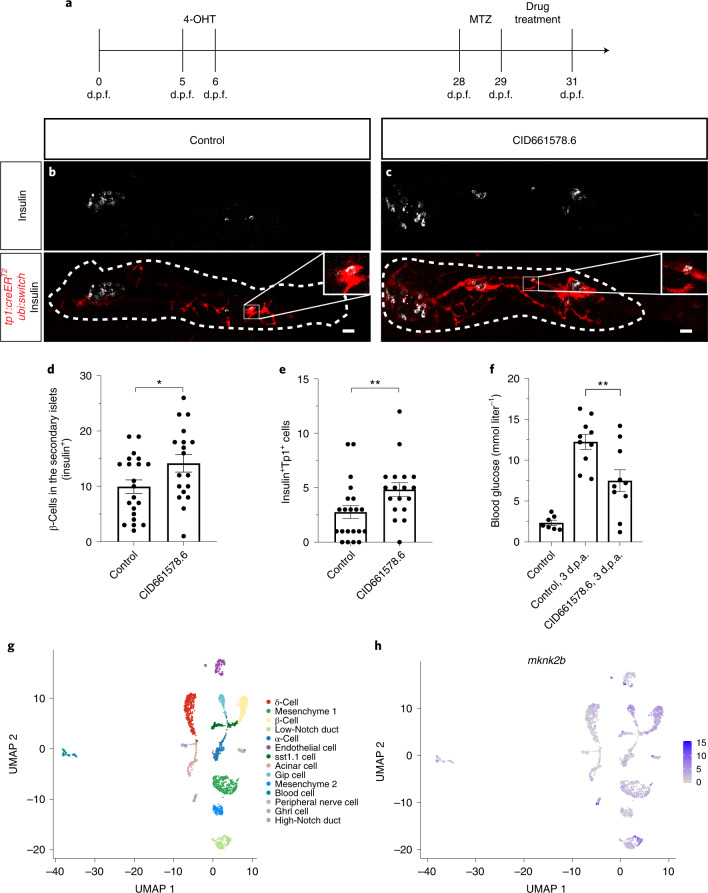
CID661578.6 increases β-cell regeneration from a pancreatic ductal origin and lowers glucose levels. **a**, Schematic of the lineage tracing experiment. Briefly, larvae were treated with 4-hydroxytamoxifen (4-OHT) for 24 h (5-6 d.p.f.) to induce recombination of the reporter. At 28 d.p.f., the fish were treated with MTZ for 24 h to ablate the β-cells, followed by 48 h of treatment with DMSO or CID661578.6. **b**–**e**, Representative images of *Tg(ubi:switch)*; *Tg(tp1:creER*^*T2*^*)*; *Tg(ins:flag-NTR)* fish treated with DMSO (**b**) or 2 µM CID661578.6 (**c**) and immunostained for insulin at 31 d.p.f.; scale bars, 20 µm. Quantifications of the number of β-cells in the secondary islets along the tail of the pancreas (**d**) as well as the number of β-cells derived from Notch-responsive cells (**e**) are shown; *n* = 21 (control) and *n* = 18 (CID661578.6) for **d**–**e**. An unpaired two-tailed Student’s *t*-test was used to assess significance for **d** (*P = 0.0393), and a two-tailed Mann–Whitney test was used for **e** (**P = 0.0087). Data are presented as mean values ± s.e.m. The experiment shown in **b** and **c** was repeated twice with similar results. **f**, Blood glucose was measured 3 d post-β-cell ablation (d.p.a.) in 4-month-old fish treated with DMSO or CID661578.6. Blood glucose levels in zebrafish without β-cell ablation were included as a basal-state reference; *n* = 7 (control), *n* = 10 (control, 3 d.p.a.), *n* = 10 (CID661578.6, 3 d.p.a.). A one-way ANOVA followed by Šidák’s multiple comparisons test was used to assess significance for **f** (**P = 0.0078). Data are presented as mean values ± s.e.m. **g**,**h**, UMAP plots showing the different cell types present in the adult zebrafish pancreas after reanalysis of published single-cell RNA-seq data (**g**) and expression of *mknk2b* (**h**) at various levels in the different clusters. Source data

**Extended Data Fig. 2:**
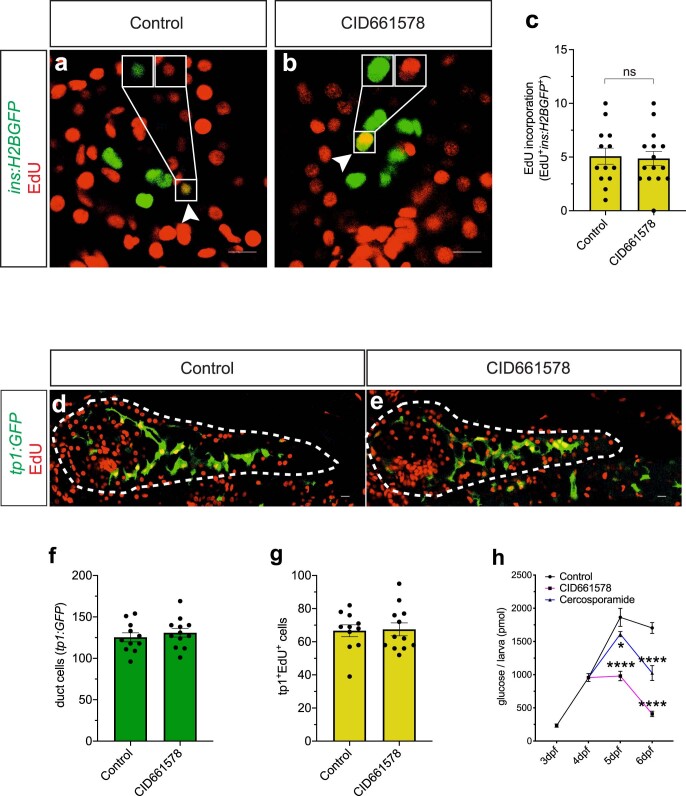
CID661578 does not induce β- or ductal-cell proliferation. **a-c**, Representative confocal images of *Tg(ins:H2BGFP);Tg(ins:flag-NTR)* larvae at 6 dpf after β-cell ablation (3-4 dpf) and incubation with EdU together with DMSO (**a**) or 5 µM CID661578 (**b**) during the regenerative period (4-6 dpf). β-cells that proliferated are marked by EdU and displayed as yellow overlap (arrowheads). The number of β-cells that proliferated was not altered by CID661578 (**c**). Scale bar, 10 µm. *n* = 13 (Control) and *n* = 15 (CID661578). Unpaired two-tailed Student’s *t* test was used to assess significance for (**c**) ns=not significant (*P* = 0.8340). Data are presented as mean values ±SEM. The experiment shown in (**a**-**b**) has been repeated two times with similar results. **d-g**, CID661578 treatment does not alter the number or the proliferation of notch-responsive ductal cells. Confocal images of DMSO- (**d**) or CID661578-treated (**e**) *Tg(tp1:GFP);Tg(ins:flag-NTR)* larvae at 6 dpf after β-cell ablation and incubation with EdU. White dashed lines outline the pancreata of the larvae. Scale bar, 10 µm. Quantification of the number of notch-responsive cells (**f**) and the number of proliferating, EdU^+^ notch-responsive ductal cells (**g**) per larva is shown. *n* = 11 (Control) and *n* = 12 (CID661578). Data are presented as mean values ±SEM. **h**, Glucose levels of *Tg(ins:flag-NTR)* larvae treated with DMSO, cercosporamide or CID661578 during the regenerative period (4-6 dpf). Four larvae were pooled for each replicate. *n* = 4 (all treatments – 3 dpf), *n* = 5 (all treatments – 4 dpf) *n* = 5 (all treatments – 5 dpf), *n* = 4 (Control and Cercosporamide – 6 dpf) and *n* = 4 (CID661578– 6 dpf). 2way ANOVA test followed by Tukey’s multiple comparisons test was used to assess significance for *P = 0.0430 (5 dpf Control vs Cercosporamide), ****P < 0.0001 (5 dpf Control vs CID661578), ****P < 0.0001 (6 dpf Control vs CID661578) and ****P < 0.0001 (6 dpf Control vs Cercosporamide). Data are presented as mean values ±SEM. Source data

**Extended Data Fig. 3:**
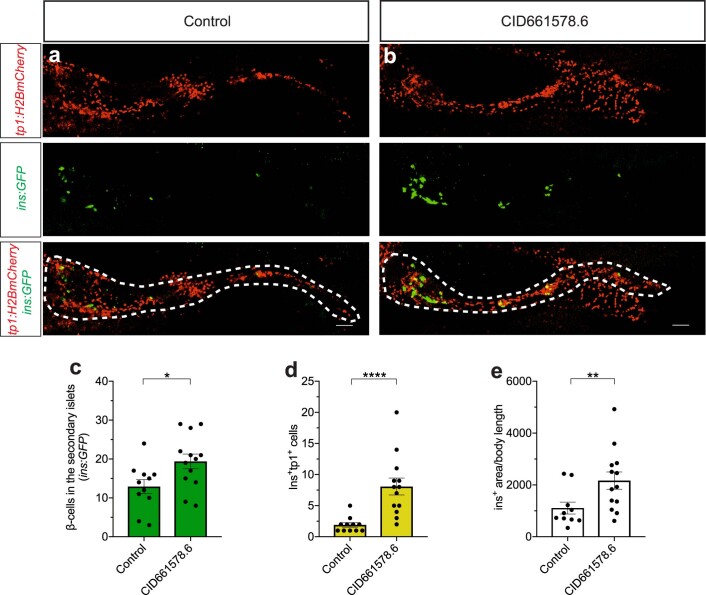
Validation of the notch-responsive cells as the source of the newly formed β-cells in juvenile fish. **a**-**e**, CID661578.6 promotes β-cell neogenesis in juvenile fish. Single-plane confocal images of *Tg(ins:GFP);Tg(tp1:H2BmCherry);Tg(ins:flag-NTR)* pancreata from 1-month-old zebrafish treated with DMSO (**a**) or CID661578.6 (**b**). Briefly, 1-month-old zebrafish were incubated with MTZ (1 mM) for 24 hours to ablate β-cells followed by chemical treatment for 2 days. Pancreata are outlined with white dashed lines. Quantification of β-cells in the secondary islets in the tail of the pancreas (**c**) shows an increase in β-cell regeneration, and quantification of the overlap between the notch-responsive cell tracer and β-cell marker demonstrated an increase in the number of β-cells derived from notch-responsive cells (**d**). CID661578.6 treatment also doubled the ins^+^ area normalized to the body length of the fish (μm^2^/mm) (**e**). Scale bar, 50 µm. (**c** and **d**), *n* = 11 (Control) and *n* = 13 (CID661578.6); (**e**), *n* = 10 (Control) and *n* = 13 (CID661578.6). Unpaired two-tailed Student’s *t* test was used to assess significance for (**c**) *P = 0.0229; two-tailed Mann-Whitney test was used for (**d**) ****P < 0.0001; two-tailed Mann-Whitney test was used for (**e**) **P = 0.0099. Data are presented as mean values ±SEM. Source data

To identify the cellular source of the newly formed β-cells, we first assessed their proliferation status. We used the β-cell ablation zebrafish model, *Tg(ins:flag-NTR)*, in which the enzyme nitroreductase (NTR) is expressed under the control of the β-cell-specific insulin promoter. When the prodrug metronidazole (MTZ) is administered, NTR converts it to a toxic byproduct, resulting in the specific ablation of β-cells^14,15^. We treated the zebrafish β-cell ablation model with CID661578 in the presence of EdU to label dividing cells, and quantification of EdU^+^ β-cells showed no alteration in proliferation status between the control and CID661578-treated groups (Extended Data Fig. 2a–c). Next, we performed lineage tracing of the pancreatic ductal cell population using the inducible *Tg(tp1:creER*^*T2*^*)* line (*tp1* is a Notch-responsive element characterizing the duct population) and the responder *Tg(ubi:switch)* line. We induced Cre-mediated recombination (5–6 days postfertilization (d.p.f.)) and let the larvae grow to the juvenile stage, followed by β-cell ablation and treatment with CID661578.6 (Fig. 2a). Quantification of β-cells along the tail of the pancreas (often referred to as secondary islets) revealed that treatment with CID661578.6 increased the number of regenerating β-cells derived from the ductal cell population (Fig. 2b–e). Moreover, we used a complementary lineage tracing approach to validate these findings, where double-transgenic *Tg(tp1:H2BmCherry)*; *Tg(ins:GFP)* zebrafish in which *tp1* drives the expression of the stable fluorescent protein H2BmCherry as a ductal cell tracer were used, and we confirmed ductal-derived β-cell regeneration (Extended Data Fig. 3a–e). Furthermore, we excluded the possibility that the chemical treatment altered the number and proliferation of the Notch-responsive ductal cells by assaying for EdU incorporation into *Tg(tp1:GFP)* zebrafish (Extended Data Fig. 2d–g). Thus, by using two different lineage tracing strategies, we identified ductal cells as the cellular source of the newly formed β-cells.

**Extended Data Fig. 4:**
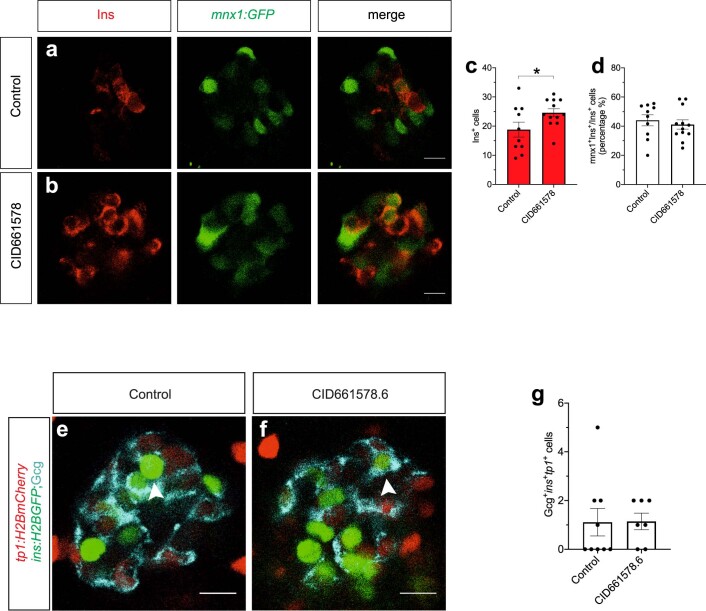
Newly-derived β-cells express *mnx1*, another marker for β-cell identity/maturation, and are devoid of glucagon expression. **a-d**, Single-plane confocal images of 6 dpf *Tg(mnx1:GFP);Tg(ins:flag-NTR)* larvae treated with DMSO (**a**) or CID661578 (**b**) (4-6 dpf) after β-cell ablation (3-4 dpf) and staining for insulin. Quantification results showed that CID661578 treatment increased the number of β-cells (**c**), but there was no difference in the percentage of *mnx1*:GFP^+^, insulin^+^ β-cells (**d**). Scale bar, 10 µm. For (**c** and **d**) *n* = 10 (Control) and *n* = 12 (CID661578). Unpaired two-tailed Student’s *t* test was used to assess significance for (**c**) *P = 0.0497. Data are presented as mean values ±SEM. **e-g**, Representative confocal images of 6 dpf *Tg(ins:H2BGFP);Tg(tp1:H2BmCherry);Tg(ins:flag-NTR)* larvae treated with DMSO (**e**) or CID661578.6 (**f**) (4-6 dpf) after β-cell ablation (3-4 dpf) and stained for glucagon. White arrowheads point to bihormonal cells (glucagon+, *ins:*H2BGFP+) derived from notch-responsive cells. Quantification of bihormonal cells derived from notch-responsive cells (**g**) showed no difference between treatments. Scale bar, 10 µm. *n* = 9 (Control) and *n* = 7 (CID661578.6). Data are presented as mean values ±SEM. Source data

We subsequently assessed glucose levels after ablation of β-cells and treatment of adult fish with CID661578.6 for 3 d. As expected, β-cell ablation caused an increase in blood glucose at 3 d after ablation in the control fish, whereas fish treated with CID661578.6 had significantly lower blood glucose than controls (Fig. 2f). Glucose levels were also reduced in zebrafish larvae following CID661578 or cercosporamide (**4**) (a previously described selective inhibitor of MNK2 (ref. ^16^)) treatment (Extended Data Fig. 2h). The newly formed β-cells also appeared mature in terms of *mnx1* expression (Extended Data Fig. 4a–d) as well as being devoid of glucagon and insulin coexpression (Extended Data Fig. 4e–g). Taken together, these results show that compounds interfering with Mnk2 lower glucose levels in both larval and older zebrafish.

**Extended Data Fig. 5:**
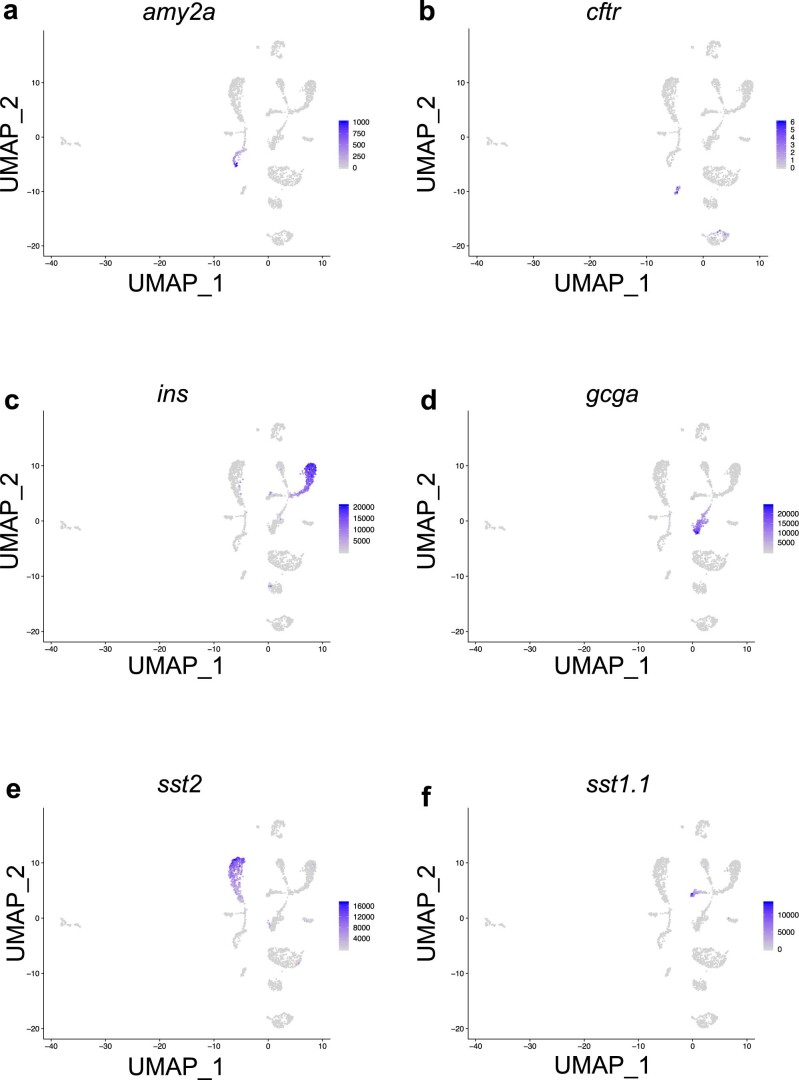
Gene expression of the primary pancreatic lineage genes in the single-cell RNA-Seq dataset. **a**-**f**, UMAPs showing the expression of *amy2a* (acinar cells) (**a**), *cftr* (ductal cells) (**b**), *ins* (β-cells) (**c**), *gcga* (α-cells) (**d**), *sst2* (δ-cells) (**e**) and *sst1.1* (**f**) in the dataset used to analyze *mknk2b* expression in adult zebrafish pancreata.

Next, we explored *mknk2b* expression in the adult zebrafish pancreas using a recently published single-cell RNA-sequencing (RNA-seq) dataset^17^ that contains all the major pancreatic cell types (Fig. 2g and Extended Data Fig. 5). The expression of *mknk2b* was not ductal specific, yet its highest expression was in a cluster of ductal cells (Fig. 2h). Taken together, these results showed that CID661578.6 increased the regeneration of functional β-cells in both larval and older zebrafish by promoting β-cell neogenesis from a ductal origin.

### CID661578 targets Mnk2b in vivo to drive β-cell regeneration

**Fig. 3:**
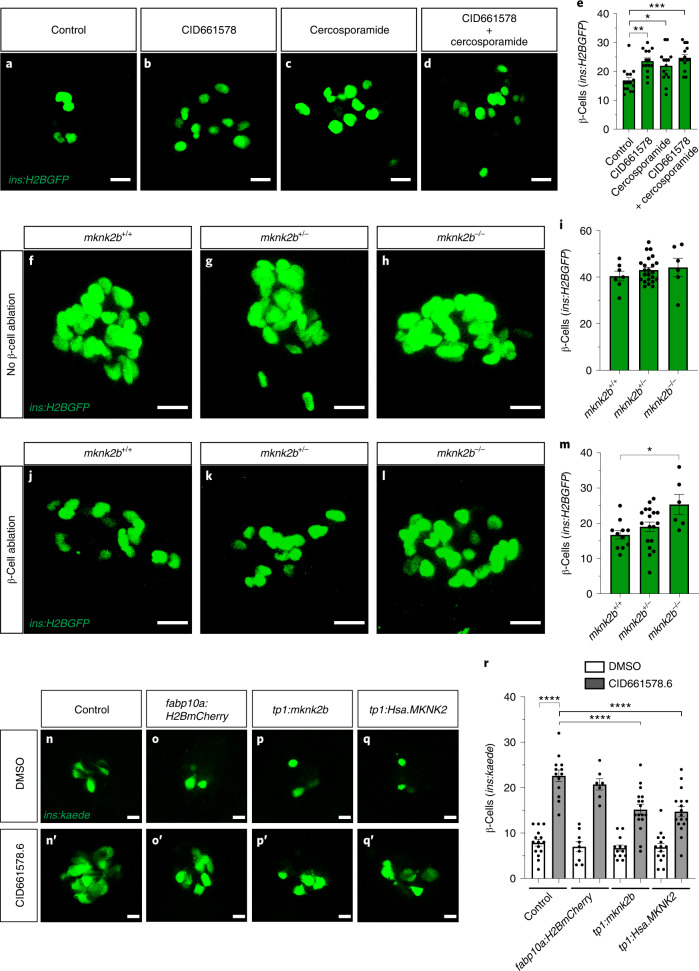
CID661578 targets Mnk2b in vivo to promote β-cell regeneration. **a**–**e**, Representative images of *Tg(ins:H2BGFP)*; *Tg(ins:flag-NTR)* larvae treated with MTZ from 3 to 4 d.p.f. to ablate β-cells, followed by treatment with DMSO (**a**), 10 µM CID661578 (**b**), 500 nM cercosporamide (**c**) or a combination of drugs thereof (**d**) for 2 d; scale bars, 10 µm. Quantification of the regenerated β-cells is shown in **e**; *n* = 15 (control), *n* = 14 (CID661578), *n* = 14 (cercosporamide) and *n* = 14 (CID661578 + cercosporamide). A Kruskal–Wallis test followed by Dunn’s multiple comparisons test was used to assess significance for **e** (**P = 0.0027, *P = 0.0280 and *** *P* = 0.0003). Data are presented as mean values ± s.e.m. **f**–**i**, Representative maximum projections of *mknk2b*^+/+^ (**f**), *mknk2*^+/–^ (**g**) and *mknk2b*^−/−^ (**h**) *Tg(ins:H2BGFP)*; *Tg(ins:flag-NTR)* 6 d.p.f. larvae. Quantification of the β-cell number in the basal state for all genotypes is shown in **i**; scale bars, 10 µm; *n* = 7 (*mknk2*^+/+^), *n* = 23 (*mknk2b*^+/–^) and *n* = 6 (*mknk2b*^–/–^). Data are presented as mean values ± s.e.m. **j**–**m**, Representative maximum projections of *mknk2b*^+/+^ (**j**), *mknk2b*^+/–^ (**k**) and *mknk2b*^–/–^ (**l**) *Tg(ins:H2BGFP)*; *Tg(ins:flag-NTR)* 6 d.p.f. larvae following 2 d of β-cell regeneration. Quantification of the β-cell number for all genotypes is shown in **m**; scale bars, 10 µm; *n* = 11 (*mknk2b*^+/+^), *n* = 18 (*mknk2b*^+/–^) and *n* = 6 (*mknk2b*^–/–^). A Kruskal–Wallis test followed by Dunn’s multiple comparisons test was used to assess significance for **m** (*P = 0.0198). Data are presented as mean values ± s.e.m. **n**–**r**, Single-plane confocal images of *Tg(ins:H2BGFP)*; *Tg(ins:flag-NTR)* larvae treated with DMSO (**n**–**q**) or CID661578.6 (**n′**–**q′**) that were uninjected (**n** and **n′**) or injected at the one-cell stage with control *fabp10a*:*H2BmCherry* (**o** and **o′**), *tp1*:*mknk2b* (**p** and **p′**) or *tp1*:*Hsa.MKNK2* (**q** and **q′**) vectors together with transposase mRNA to induce mosaic overexpression of the zebrafish Mnk2b or the human MNK2 in Notch-responsive cells. Quantification results revealed that overexpression of either *mknk2b* or *MKNK2* significantly blocked the effect of CID661578.6 on β-cell regeneration (**r**); scale bars, 10 µm; *n* = 15 (control + DMSO), *n* = 13 (control + CID661578.6), *n* = 9 (*fabp10a*:*H2BmCherry* + DMSO), *n* = 7 (*fabp10a*:*H2BmCherry* + CID661578.6), *n* = 13 (*tp1*:*mknk2b* + DMSO), *n* = 17 (*tp1*:*mknk2b* + CID661578.6), *n* = 14 (*tp1*:*Hsa.MKNK2* + DMSO) and *n* = 17 (*tp1*:*Hsa.MKNK2* + CID661578.6). A one-way ANOVA followed by Tukey’s multiple comparisons test was used to assess significance for **r** (****P < 0.0001 for control + DMSO versus control + CID661578.6, control + CID661578.6 versus *tp1*:*mknk2b* + CID661578.6 and control + CID661578.6 versus *tp1*:*Hsa.MKNK2* + CID661578.6). Data are presented as mean values ± s.e.m. Source data

**Extended Data Fig. 6:**
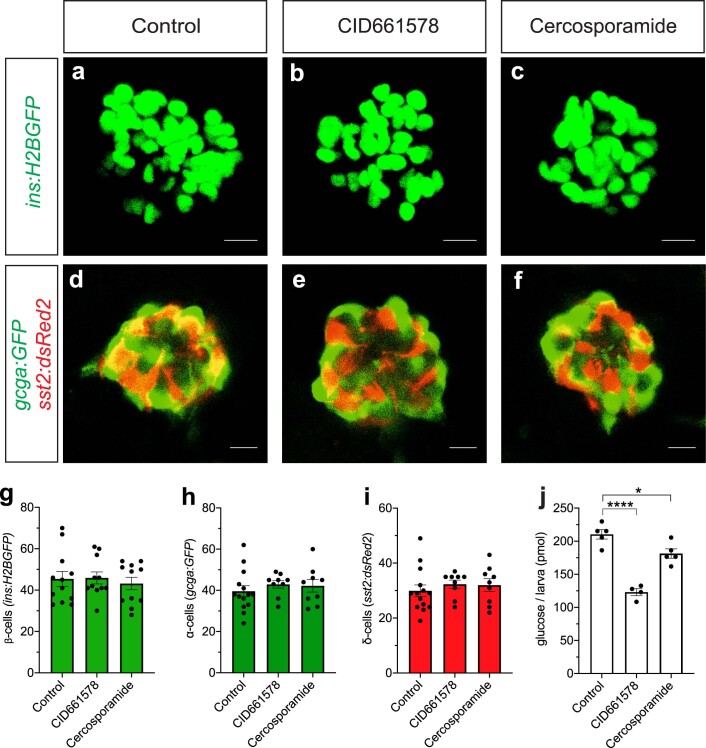
CID661578-treated zebrafish in the basal state exhibit no changes in endocrine cell numbers. **a**-**c,g**, Maximum projections confocal images of primary islets in *Tg(ins:H2BGFP)* larvae at 6 dpf after treatment with (**a**) DMSO, (**b**) CID661578 or (**c**) cercosporamide from 3-6 dpf. Quantification (**g**) of the total number of β-cells revealed no changes resulting from the treatments. Scale bar, 10 µm. *n* = 12 (Control), *n* = 11 (CID661578) and *n* = 11 (Cercosporamide). Data are presented as mean values ±SEM. **d-f,h-i**, Single-plane confocal images of primary islets in *Tg(gcga:GFP);Tg(sst2:dsRed2)* larvae at 6 dpf after treatment with (**d**) DMSO, (**e**) CID661578 or (**f**) cercosporamide from 3-6 dpf. Quantification of the total number of α-cells (**h**) and δ-cells (**i**) revealed no changes resulting from the treatments. Scale bar, 10 µm. For both (**h** and **i**), *n* = 14 (Control), *n* = 9 (CID661578) and *n* = 9 (Cercosporamide). Data are presented as mean values ±SEM. **j**, Glucose measurements in zebrafish larvae in the basal state treated with DMSO, CID661578 or cercosporamide for 1 day (3-4 dpf). Four larvae were pooled for each replicate. *n* = 5 (Control), *n* = 4 (CID661578) and *n* = 5 (Cercosporamide). One-way ANOVA test followed by Dunnett’s multiple comparisons test was used to assess significance for (**j**) ****P < 0.0001 (Control vs CID661578); *P = 0.0182 (Control vs Cercosporamide). Source data

Subsequently, we wanted to determine whether the in vivo engagement of Mnk2b is responsible for the observed phenotypes of CID661578 treatment. We treated zebrafish larvae with CID661578, cercosporamide or their combination from 4 to 6 d.p.f. during β-cell regeneration. We observed a dramatic increase in regenerating β-cells in the primary islets of zebrafish larvae following treatment with either chemical, but no additive effect was observed when they were combined (Fig. 3a–e). This result suggested that cercosporamide and CID661578 have similar effects on β-cell regeneration, and the absence of additive/synergistic effects indicated that they converge on a common molecular target/pathway. Further, we found that neither CID661578 nor cercosporamide treatment affected the development of any of the endocrine cell populations in zebrafish larvae, suggesting that the effects of this pathway are restricted to the regenerative state (Extended Data Fig. 6a–i).

**Extended Data Fig. 7:**
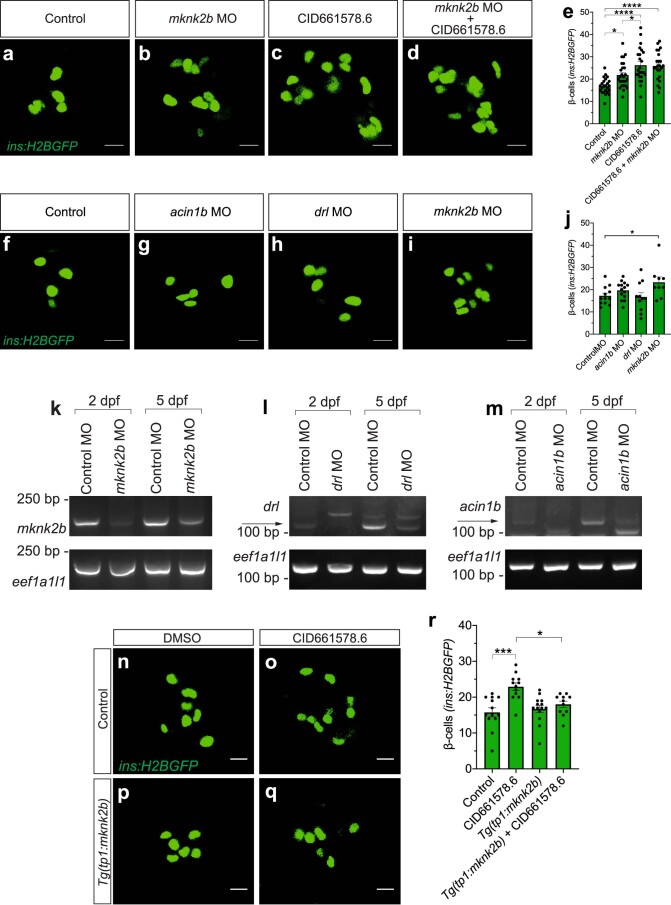
Morpholino-mediated knockdown of the A-hits from the yeast screen show that *mknk2b*, but not *acin1b* and *drl*, affects β-cell regeneration. **a**-**e**, *Tg(ins:H2BGFP);Tg(ins:flag-NTR)* embryos were injected at the 1-cell stage with a control morpholino (**a,c**) or a morpholino against *mknk2b* (**b,d**), β-cells ablated between 2-3 dpf, and treated with DMSO (**a**,**b**) or 2 µM CID661578.6 (**c**,**d**) for 2 days. Quantification of regenerated β-cells (**e**) revealed an increase upon *mknk2b* knockdown, similar to the chemical inhibition of Mnk2. No additive effect was observed with the combined *mknk2b* MO and CID661578.6 treatment. Scale bar, 10 µm. *n* = 24 (Control), *n* = 26 (*mknk2b* MO), *n* = 24 (CID661578.6) and *n* = 23 (*mknk2b* MO + CID661578.6). *P = 0.0210 (control vs *mknk2b* MO) and 0.0459 (*mknk2b* MO vs CID661578.6), ****P < 0.0001. Data in this graph are pooled from two independent experiments and are presented as mean values ±SEM. **f-j**, Confocal images of primary islets of *Tg(ins:H2BGFP);Tg(ins:flag-NTR)* at 5 dpf, after injection at the one-cell stage with control (**f**), *acin1b* (**g**), *drl* (**h**) or *mknk2b* (**i**) morpholinos. Following the ablation of β-cells, only *mknk2b* knockdown significantly increased β-cell regeneration (**j**). Scale bar, 10 µm. *n* = 12 (Control MO), *n* = 14 (*acin1b* MO), *n* = 11 (*drl* MO) and *n* = 9 (*mknk2b* MO). (**j**) *P = 0.0410. Data are presented as mean values ±SEM. **k-m**, Agarose gel images of RT-qPCR validating the knockdown of *mknk2b* (**k**), *drl* (**l**) and *acin1b* (**m**) at two stages (2 dpf and 5 dpf). Amplification of *eef1a1l1* was used as control. Arrows point to the band of the expected size. The experiment shown in (**k**-**m**) has been repeated twice with similar results. **n-r**, Single-plane confocal images of *Tg(ins:H2BGFP);Tg(ins:flag-NTR)* islets from control (**n** and **o**) or larvae overexpressing *mknk2b* in notch-responsive cells (**p** and **q**), treated with DMSO (**n** and **p**) or CID661578.6 (**o** and **q**). Quantification results (**r**) showed that the stable *Tg(tp1:mknk2b)* overexpression blocked the effect of CID661578.6 on β-cell regeneration, similar to that observed in the transient overexpression model (see Fig. 3). Scale bar, 10 µm. *n* = 13 (Control), *n* = 12 (CID661578.6), *n* = 14 (*Tg(tp1:mknk2b)*) and *n* = 11 (*Tg(tp1:mknk2b)*+CID661578.6). (**r**)***P = 0.0001 and *P = 0.0172. Data are presented as mean values ±SEM. Source data

We also tested if the effects of the chemicals targeting Mnk2 could be reproduced using genetic approaches. The *mknk2* gene is duplicated in the zebrafish genome, and an investigation of the expression of the two paralogs *mknk2a* and *mknk2b* in published RNA-seq data revealed that *mknk2b* is the predominantly expressed paralog in ductal cells^17^. Therefore, we generated a *mknk2b* full-body knockout zebrafish using CRISPR–Cas9 mutagenesis to target the N-terminal part of the protein. First, we observed physiological β-cell development in the homozygous mutants (Fig. 3f–i). However, β-cell regeneration was enhanced in the homozygous *mknk2b* mutants following β-cell ablation (Fig. 3j–m), suggesting that the absence/inhibition of Mnk2b is responsible for this phenotype. We reproduced the *mknk2b*-knockout phenotype using a splice-blocking morpholino to knockdown *mknk2b* (validated by quantitative PCR with reverse transcription (RT–qPCR) (Extended Data Fig. 7k)). Knockdown of *mknk2b* increased the number of β-cells, while no additive effect was observed from simultaneous *mknk2b* knockdown and CID661578.6 treatment (Extended Data Fig. 7a–e). In addition, morpholino knockdown of the other two A hits from the zebrafish library, *acin1b* and *drl*, did not increase β-cell regeneration, suggesting that these two genes are not responsible for the observed phenotypes (Extended Data Fig. 7f–m). These results further supported that β-cell regeneration increased in the absence of *mknk2b*.

Finally, we reasoned that because the knockout and knockdown of *mknk2b* had similar effects as CID661578.6 treatment, overexpression of the protein would sequester CID661578.6 and reduce β-cell regeneration. To this end, we cloned zebrafish *mknk2b* and human *MKNK2* and overexpressed them under the control of the *tp1* promoter in the β-cell ablation model, which was subsequently treated with CID661578.6. Overexpression of either *mknk2b* or *MKNK2* significantly decreased the effect of CID661578.6 on β-cell regeneration (Fig. 3n–r). These experiments using mosaic overexpression were also confirmed in a stable line overexpressing *mknk2b*, which showed an even stronger reversal of the CID661578.6 effect on β-cell regeneration (Extended Data Fig. 7n–r). Collectively, these data support Mnk2b as the molecular target of CID661578.6 in vivo and that Mnk2b can restrict β-cell neogenesis from a ductal origin.

### CID661578 boosts translation to increase β-cell regeneration

**Fig. 4:**
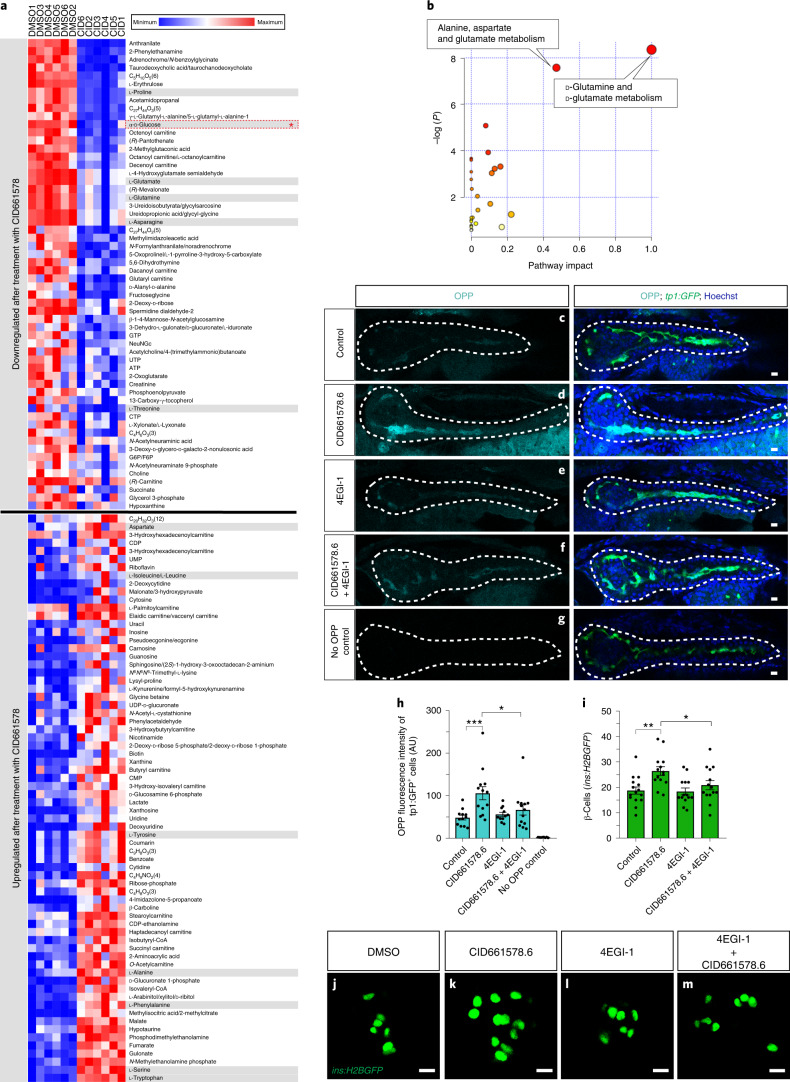
CID661578 boosts protein synthesis to increase β-cell regeneration. **a**, Heat map showing significantly downregulated and upregulated metabolites following treatment with CID661578 (*t*-test analyses). Pools of 10 wild-type larvae at 5 d.p.f. were used for each of the six independent biological replicates for DMSO (DMSO1–DMSO6) or CID661578 (CID1–CID6) treatment from 4 to 5 d.p.f. Gray shading highlights the amino acids regulated in the samples, and the red asterisk highlights the glucose metabolite. **b**, Pathway analysis assessing 81 characterized metabolic pathways in zebrafish using the significantly downregulated metabolites. Boxes show the most significantly affected pathways (false discovery rate < 0.05) following treatment with CID661578. **c**–**h**, Single-plane confocal images of *Tg(tp1:GFP)*; *Tg(ins:flag-NTR)* pancreata from 5 d.p.f. larvae incubated with OPP for 18 h to label protein synthesis during treatment with DMSO (**c**), CID661578.6 (**d**), 4EGI-1 (**e**) or CID661578.6 together with 4EGI-1 (**f**). Larvae that were not incubated with OPP but were developed to visualize the fluorophore (**g**) were used as controls to assess background staining. White dashed lines outline the pancreata of the larvae. Quantification of the OPP fluorescence intensity levels in the Notch-responsive cells is shown in **h**; scale bars, 10 µm; *n* = 12 (control), *n* = 13 (CID661578.6), *n* = 11 (4EGI-1), *n* = 13 (CID661578.6 + 4EGI-1) and *n* = 8 (no OPP control); AU, arbitrary units. A one-way ANOVA followed by Šidák’s multiple comparisons test was used to assess significance for **h** (***P = 0.0004 and *P = 0.0151). Data are presented as mean values ± s.e.m. **i**–**m**, Representative images of *Tg(ins:H2BGFP)*; *Tg(ins:flag-NTR)* larvae treated with DMSO (**j**), CID661578.6 (**k**), 4EGI-1 (**l**) or CID661578.6 together with 4EGI-1 (**m**) for 2 d following β-cell ablation. Quantification of the number of β-cells (**i**) showed that 4EGI-1 treatment could abolish the effect of CID661578.6 on β-cell regeneration; scale bars, 10 µm; *n* = 15 (control), *n* = 14 (CID661578.6), *n* = 14 (4EGI-1) and *n* = 14 (CID661578.6 + 4EGI-1). A one-way ANOVA followed by Šidák’s multiple comparisons test was used to assess significance for **i** (**P = 0.0022 and *P = 0.0341). Data are presented as mean values ± s.e.m. Source data

**Extended Data Fig. 8:**
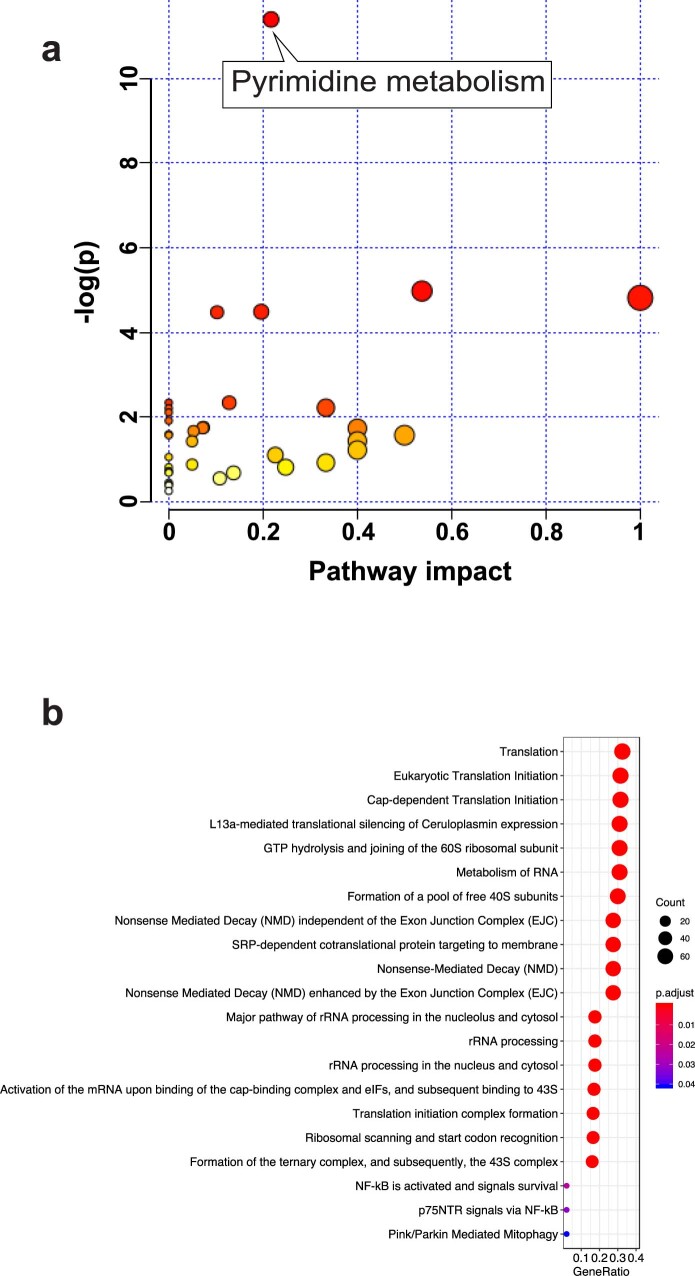
Enrichment analysis for the upregulated metabolites and differentially expressed genes in the single-cell RNA-Seq data. **a**, Pathway analysis, assessing 81 characterized metabolic pathways in zebrafish, using the significantly upregulated metabolites. Only pyrimidine metabolism was significantly enriched in this dataset (FDR < 0.05). **b**, Enrichment analysis for KEGG pathways in differentially expressed genes, that is, genes upregulated in ductal cells (‘low-notch’ cluster, see Fig. 2g) compared to all endocrine cells.

To better understand the molecular mechanism induced by CID661578, we treated zebrafish larvae with CID661578 for 24 h before global metabolomics characterization^18^. After creating a metabolite profile of CID661578-treated zebrafish, we identified differentially regulated metabolites (Fig. 4a). An interesting observation was that the levels of many amino acids were altered following CID661578 treatment. MNK2 interacts with a complex of eukaryotic translation initiation factors and thereby plays a role in protein synthesis^19^. Thus, the metabolomics data indicated possible changes in protein synthesis as a key effect of CID661578, consistent with the known role of MNK2. Moreover, α-d-glucose was significantly downregulated, an observation that we replicated using glucose measurements with an in vitro assay for both CID661578 and cercosporamide (Fig. 4a and Extended Data Fig. 6j). Metabolite set enrichment analysis of zebrafish-specific pathways showed that downregulated metabolites following CID661578 treatment were related to non-essential amino acid metabolism (Fig. 4b), while the pathways related to upregulated metabolites were less impacted and limited to changes in pyrimidine metabolism (Extended Data Fig. 8a). Further, enrichment analysis of the single-cell RNA-seq data used to examine the expression of *mknk2b* showed that genes enriched in the ductal cells have a role in mRNA translation (Extended Data Fig. 8b). Taken together, these results further strengthen the hypothesis that CID661578 affects global changes in protein synthesis and glucose metabolism, in agreement with protein synthesis being a highly energy-consuming process that should reduce nutrient levels, including glucose.

To further investigate the changes in protein synthesis in vivo, we measured the incorporation of *O*-propargyl-puromycin (OPP), a modified amino acid that is incorporated in proteins during translation and can be visualized with a Click-iT reaction. Following β-cell ablation, larvae were incubated with OPP for 20 h concomitant with treatments of CID661578.6, 4EGI-1 or their combination. 4EGI-1 inhibits the interaction between the translation initiation factors eIF4E and eIF4G, which together with MNK2 function within the translation initiation complex^20^. The rationale of this assay was to assess the outcome of altering the translation initiation complex composition during CID661578.6 treatment. OPP incorporation was predominantly observed in the ductal cells of the pancreas, indicating a high protein synthesis rate in this population. CID661578.6 treatment drastically increased the incorporation of OPP in the ductal cells (and the intestine), an effect abolished following cotreatment with the inhibitor 4EGI-1 (Fig. 4c–h). These data suggest that CID661578.6 boosts protein synthesis and that its effect is dependent on the translation initiation complex.

Encouraged by the observation that 4EGI-1 inhibited CID661578.6-induced protein synthesis, we examined whether 4EGI-1 could also inhibit the induced β-cell regeneration. To this end, zebrafish larvae were treated during the regenerative period with CID661578.6, 4EGI-1 or their combination. Interestingly, 4EGI-1 treatment was also sufficient to inhibit CID661578.6-induced β-cell regeneration (Fig. 4i–m). Thus, through a combination of metabolomics and protein synthesis measurements, we demonstrated that CID661578.6 induces protein synthesis in vivo and that the effect on both protein synthesis and β-cell regeneration is blocked by targeting the translation initiation complex that MNK2 is a part of.

### CID661578.6 modulates the translation initiation complex

**Fig. 5:**
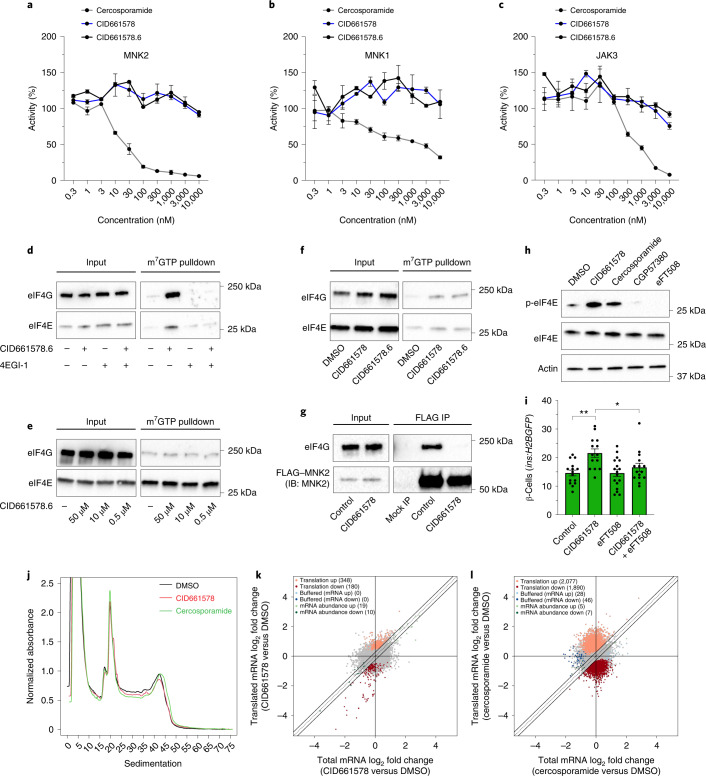
CID661578 increases the interaction between eIF4G and eIF4E and leads to translational changes, without affecting the kinase activity of MNK2. **a**–**c**, Dose–response of CID661578, CID661578.6 or cercosporamide on MNK2 (**a**), MNK1 (**b**) and JAK3 (**c**) kinase activity in vitro; *n* = 2 for each concentration tested. Data are presented as mean values ± s.e.m. **d**, Immunoblotting against eIF4G and eIF4E after an m^7^GTP pulldown assay in lysates of COLO 320HSR cells after 6-h treatment with DMSO, CID661578.6, 4EGI-1 or CID661578.6 together with 4EGI-1. For a loading control, 5% of the input was used. **e**, Immunoblotting against eIF4G and eIF4E after an m^7^GTP pulldown assay in rabbit reticulocytes treated with the indicated concentrations of CID661578.6. **f**, Immunoblotting against eIF4G and eIF4E after an m^7^GTP pulldown assay in lysates of PANC-1 cells treated with DMSO, CID661578 or CID661578.6 for 6 h. For a loading control, 1% of the input was used. **g**, Immunoblotting against eIF4G and FLAG–MNK2 after an immunoprecipitation (IP) assay with anti-FLAG in lysates of PANC-1 cells that were treated for 6 h with DMSO or CID661578. For a loading control, 1% of the input was used; IB, immunoblot. **h**, Immunoblotting against phospho-eIF4E (Ser 209; p-eIF4E), total eIF4E and actin in lysates of PANC-1 cells after 6-h treatment with DMSO, CID661578, cercosporamide, CGP57380 or eFT508. **i**, Quantification of the number of β-cells in 6 d.p.f. zebrafish larvae following β-cell ablation and treatment for 48 h with DMSO, CID661578, eFT508 or a combination of CID661578 and eFT508; *n* = 15 (control), *n* = 14 (CID661578), *n* = 17 (eFT508) and *n* = 15 (CID661578.6 + eFT508). A one-way ANOVA followed by Dunnett’s multiple comparisons test was used to assess significance for **i** (**P = 0.0014 (control versus CID661578) and *P = 0.0283 (CID661578 versus CID661579 + eFT508)). Data are presented as mean values ± s.e.m. Experiments in **d**–**h** were repeated at least two times. **j**, Representative polysome tracings from optimized sucrose gradients of PANC-1 cells treated with DMSO, CID661578 or cercosporamide. **k**,**l**, Scatter plots showing log_2_ fold changes for total mRNA (*x* axis) and polysome-associated mRNA (*y* axis) for the comparisons of CID661578 (**k**) and cercosporamide (**l**) to DMSO. Color codes indicate significantly affected mRNAs identified by anota2seq analysis. Source data

**Extended Data Fig. 9:**
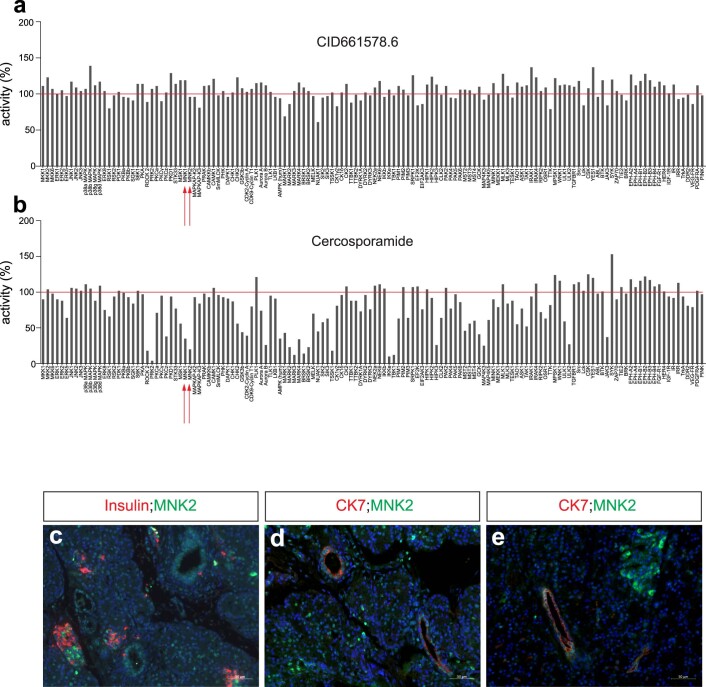
CID661578.6 does not potently affect the activity of any kinase in an in vitro screen; and MNK2 expression in the pig pancreas. **a**,**b**, An in vitro screen to assess the specificity of 1 µM CID661578.6 and 1 µM cercosporamide against a panel of 140 kinases. CID661578.6 did not alter the activity of any kinase by more than 50% (**a**), while cercosporamide inhibited the activity of several kinases, including MNK1 and MNK2 (**b**). Red arrows point to MNK1 and MNK2. **c-e**, Images of neonatal (**c**,**d**) and adult (**e**) pig pancreatic sections immunostained for MNK2, with insulin used as a marker of β-cells and CK7 as a marker of ductal cells. Scale bar, 50 µm. Similar staining results were obtained in sections from multiple pig pancreata. Source data

We sought to identify how interference with MNK2 affects translation initiation complex composition. First, we performed an in vitro characterization of the kinase activity of MNK2 in the presence of CID661578, CID661578.6 and the known MNK2 inhibitor cercosporamide. We decided to assess the inhibitory effect of the chemicals on the kinase activities of MNK2, MNK1 and JAK3, which all have been shown to be inhibited by cercosporamide^16^. We observed that the kinase activity of all three kinases remained unchanged after treatment with either CID661578 or CID661578.6, whereas cercosporamide potently inhibited MNK2 and partially inhibited MNK1 and JAK3 (Fig. 5a–c). Subsequently, we performed a kinome screen in which we assayed the kinase activity of 140 human kinases after treatment with CID661578.6 or cercosporamide. We did not observe any drastic changes in kinase activity with CID661578.6 treatment, whereas cercosporamide decreased the kinase activity of numerous kinases (Extended Data Fig. 9a,b). These results suggest that CID661578.6 does not affect protein synthesis by inhibiting the kinase activity of MNK2.

We then reasoned that CID661578.6 binding to MNK2 could alter the composition of the translation initiation complex. Given that inhibiting the interaction between translation initiation factors eIF4E and eIF4G was sufficient to block the effects of CID661578.6 in vivo, we hypothesized that CID661578.6 binding to MNK2 may enhance the interaction between eIF4E and eIF4G at the mRNA cap. To test our hypothesis, we treated the COLO 320HSR cell line with CID661578.6, 4EGI-1 or their combination. Subsequently, we pulled down the cap-binding protein eIF4E from cell lysates using beads with an immobilized m^7^GTP structure of the mRNA cap. Treatment with CID661578.6 stabilized the eIF4E–eIF4G interaction, an effect that could be reversed following treatment with the inhibitor 4EGI-1 (Fig. 5d). Subsequently, we performed the same m^7^GTP pulldown assay in vitro using rabbit reticulocytes. The advantage of using this in vitro system is that rabbits do not have an ortholog of *MKNK2*. CID661578.6 did not increase the eIF4E–eIF4G interaction in rabbit reticulocytes, indicating that the increase in the eIF4E–eIF4G interaction after CID661578.6 treatment is dependent on MNK2 (Fig. 5e). Lastly, we asked how the interaction between CID661578 and MNK2 could affect the recruitment of MNK2 to the translation initiation complex. For this experiment, we used the human pancreatic cancer cell line PANC-1, as it was more efficiently transfected than the COLO 320HSR cell line. We began by validating that both CID661578 and its analog CID661578.6 increased the eIF4G–eIF4E interaction in PANC-1 cells by using the m^7^GTP pulldown assay (Fig. 5f). Next, we transfected PANC-1 cells with a FLAG–MNK2 plasmid, added DMSO/CID661578 and performed immunoprecipitation of MNK2 using anti-FLAG. Immunoblotting against eIF4G (which is the protein that directly interacts with MNK2 in the complex) revealed that CID661578 treatment largely abolished the interaction between MNK2 and eIF4G (Fig. 5g). Finally, we assessed whether phosphorylation of eIF4E was affected by CID661578-induced changes in the translation initiation complex composition. Unexpectedly, we observed that phosphorylation of eIF4E was increased after treatment with CID661578 and cercosporamide (that is, selective interference of MNK2) but was nearly abolished with two broader inhibitors blocking both MNK1 and MNK2 (that is, CGP57380 and eFT508; Fig. 5h). To address whether increased eIF4E phosphorylation is important for β-cell regeneration, we cotreated fish with CID661578 and eFT508 and observed that eFT508 could inhibit the increase in β-cell numbers induced by CID661578 (Fig. 5i). Taken together, these data suggest that CID661578 binds to MNK2, preventing it from interacting with eIF4G, which can increase MNK1-dependent phosphorylation of eIF4E and bolsters the interaction between eIF4E, eIF4G and the mRNA, resulting in increased protein synthesis.

**Extended Data Fig. 10:**
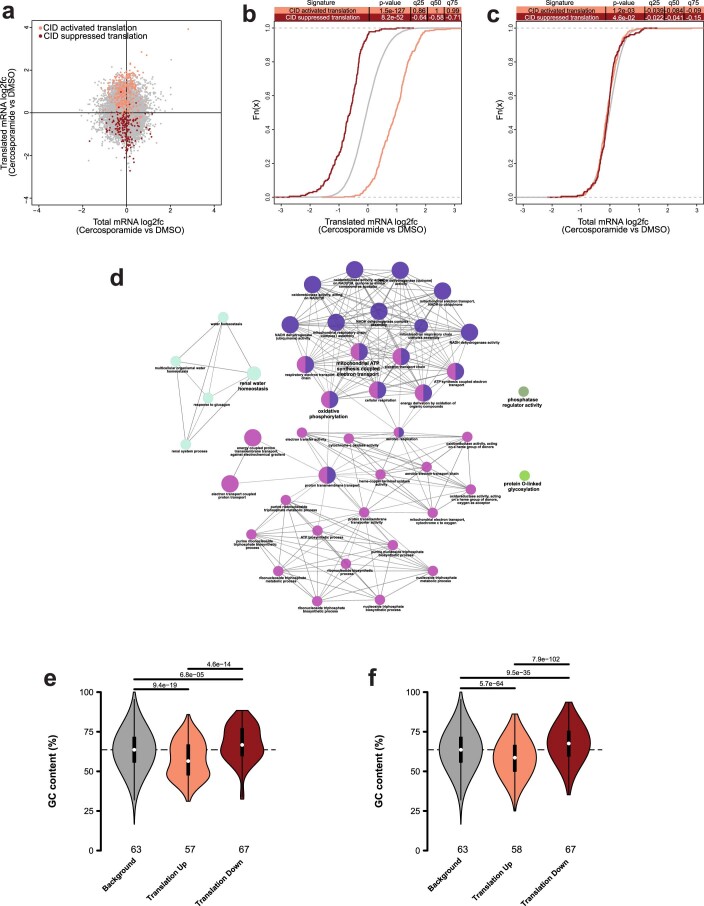
Polysome profiling expands on the effects and similarities between CID661578 and cercosporamide treatments on PANC-1 cells. **a**, Scatterplot indicating that genes translationally regulated by CID661578 are similarly regulated by treatment with cercosporamide. **b**,**c**, Empirical cumulative distributions of log2 fold changes for polysome-associated (translated) and total mRNA when comparing cercosporamide and DMSO treatments. Transcripts whose translation was suppressed (dark red) or activated (light red) by CID661578 differed from background (grey) and showed a similar directionality following cercosporamide treatment. P-values (Wilcoxon test) comparing each set to background mRNAs and shifts (log2) relative to background transcripts at multiple quartiles are also shown. **d**, Significantly enriched GO terms among proteins encoded by mRNAs that were hypotranslated upon both CID661578 and cercosporamide treatments relative to DMSO. **e,f**, Violin plots showing the 5’UTR GC percentage among transcripts translationally activated or suppressed following CID661578 (**e**) or cercosporamide (**f**) treatment. The background (grey; that is mRNAs not in regulated subsets) is also shown together with *P*-values from two-sided Wilcoxon Rank Sum test for the indicated comparisons. For (**e**): (background) *n* = 9853, minima:16,7;maxima:100;centre:63,6;upper bound:95,4;lower bound:32;25^th^ per:55,7;75^th^ per:71,6; (translation up) *n* = 319, minima:31,1;maxima:85,9;centre:56,5;upper bound:85,9;lower bound:31,1;25^th^ per:47,7;75^th^ per:66,8; (translation down) *n* = 127, minima:32,5;maxima:88,4;centre:66,7;upper bound:88,4;lower bound:33,9;25^th^ per:59,8;75^th^ per:77. For (**f**): (background) *n* = 9853, minima:16,7;maxima:100;centre:63,6;upper bound:95,4;lower bound:32;25^th^ per:55,7;75^th^ per:71,6; (translation up) *n* = 1850, minima:25;maxima:86,2;centre:58,6;upper bound:86,3;lower bound:25,3;25^th^ per:50;75^th^ per:66,5; (translation down) *n* = 1468, minima:35,2;maxima:93,7;centre:67,6;upper bound:93,7;lower bound:35,8;25^th^ per:59,6;75^th^ per:75,4. Source data

As alterations in both eIF4E phosphorylation and increased eIF4F–complex (that is eIF4E, eIF4G and eIF4A) formation affects mRNA translation in a transcript-selective fashion^21,22^, we sought to identify translationally regulated mRNAs following both CID661578 and cercosporamide treatments. To this end we performed polysome profiling of PANC-1 cells after chemical treatments and used RNA sequencing to quantify total mRNA and mRNA associated with more than three ribosomes (Fig. 5j). We then identified mRNA whose translational efficiency was modulated along with mRNAs with changed abundance and translationally buffered (Fig. 5k,l and Methods). We focused our analysis on transcripts with altered translational efficiencies predicted to affect protein levels and found that there was a highly significant overlap of hypo- and hypertranslated mRNAs between CID661578 and cercosporamide treatments (Extended Data Fig. 10a–c). Overall, we identified a total of 270 hypertranslated and 99 hypotranslated mRNAs that were shared between treatments (Supplementary Data 1–3). This further highlights that cercosporamide and CID661578 target overlapping molecular pathways in PANC-1 cells. Gene ontology (GO) analysis identified several GO terms enriched among proteins encoded by mRNAs that were hypotranslated in response to both compounds, with the most striking being mitochondrial-related processes (Extended Data Fig. 10d). By contrast, there were no significantly enriched pathways among shared hypertranslated mRNAs. Lastly, we examined the 5′ untranslated region (5′ UTR) sequences in search of features of translationally regulated mRNAs. Our analysis demonstrated differences in GC content among translationally regulated mRNAs following chemical treatments (Extended Data Fig. 10e,f), a feature underlying 5′ UTR structures. Therefore, both CID661578 and cercosporamide modulate mRNA translation in a selective fashion where the hypertranslated mRNAs had 5′ UTRs with low GC content and the hypotranslated mRNAs had 5′ UTRs with high GC content, consistent with previous studies on eIF4E-regulated translation^23,24^.

### CID661578-induced β-cell neogenesis translates to mammals

**Fig. 6:**
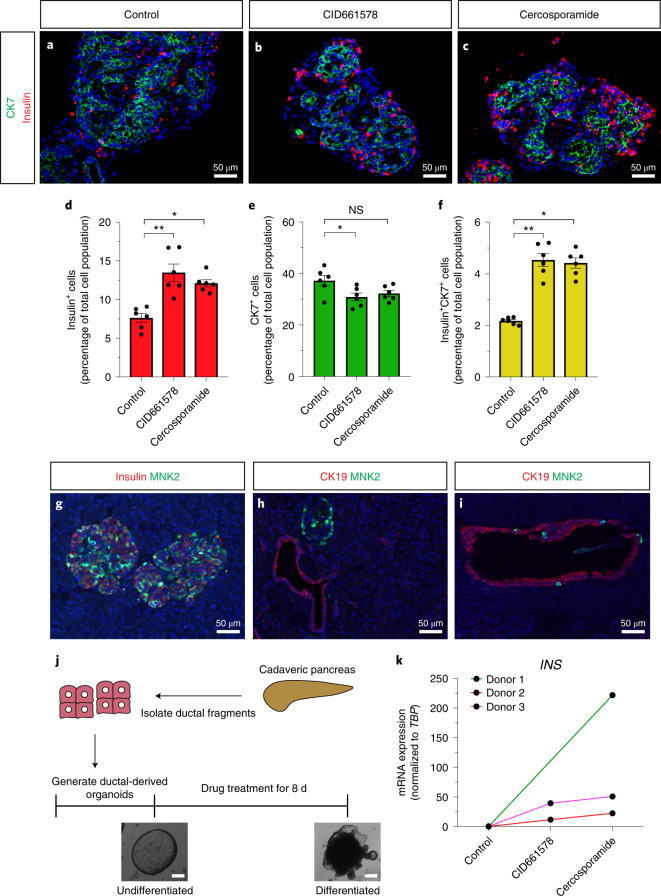
CID661578/cercosporamide treatment increases β-cell differentiation in ductal cells from neonatal pigs and human organoids. **a**–**f**, Images of neonatal pig islets treated with DMSO (**a**), CID661578 (**b**) or cercosporamide (**c**) and stained for insulin (red) and the ductal cell marker CK7 (green). Quantification results showed that treatment with either CID661578 or cercosporamide increased the number of insulin^+^ β-cells (**d**), decreased the number of CK7^+^ duct cells (**e**) and increased the number of double-positive (insulin^+^CK7^+^) cells (**f**); *n* = 6; **P = 0.0041 and *P = 0.0116 (**d**); *P = 0.0401 and *P* = 0.1671 (NS, not significant) (**e**); **P = 0.0034 and *P = 0.0137 (**f**). A Kruskal–Wallis test followed by Dunn’s multiple comparisons test was used to assess significance for **d**–**f**. Data are presented as mean values ± s.e.m. **g**–**i**, Images of human pancreatic sections from different donors stained for MNK2, with insulin used as a marker of β-cells and CK19 used to mark the pancreatic duct. Similar results have been reproduced in stainings from pancreatic sections of multiple human donors. **j**,**k**, Schema showing the procedure for generating and treating human ductal-derived organoids (**j**). Brightfield images of representative examples of human ductal-derived organoids before differentiation and after treatment with cercosporamide are shown; scale bar, 200 µm. *INS* mRNA expression is shown in **k** for three different organoid preparations (that is, from three different donors) for cercosporamide and two for CID661578. The experiment was reproducible in at least two different organoid preparations. Source data

To examine whether our findings were translatable to mammals, we took advantage of an in vitro culture system of neonatal pig islet aggregates. Pancreata from 3-d-old pigs were digested, and the islet aggregates were generated and cultured in vitro for 3 d before a 5-d treatment with CID661578 or cercosporamide. These islet aggregate preparations are highly enriched in intraislet ductal cells, making them an ideal model to study the effect of the assayed chemicals. MNK2 is expressed in the duct as well as in islets of juvenile and adult pigs (Extended Data Fig. 9c–e). Treatment with either CID661578 or cercosporamide increased the number of insulin^+^ cells in the islet aggregates (Fig. 6a–d). The number of CK7^+^ ductal cells decreased after treatment with CID661578, while the number of cells coexpressing insulin and CK7 increased following either treatment (Fig. 6e,f). These results indicated that the new β-cells also have a ductal origin in neonatal pig islets and showed that the increase of β-cells in the zebrafish could be translated to a mammalian model.

Lastly, we stained human pancreatic sections for MNK2 to assess its expression. We observed MNK2 expression in islets without prominent expression in β-cells (Fig. 6g). Interestingly, we observed a distinct strong expression of MNK2 in a sparse population of cells along the pancreatic ducts. The MNK2-expressing cells that were located just outside the luminal ductal lining most often did not coexpress MNK2 and CK19. However, we also observed MNK2^+^CK19^+^ ductal cells at other positions in the same ducts (Fig. 6h,i). To address whether CID661578 or cercosporamide can stimulate differentiation of human ductal cells toward β-cells, we generated ductal organoid cultures from healthy human donors (Fig. 6j). Encouragingly, treatment of the human organoid cultures with either CID661578 or cercosporamide increased the expression of *INS* mRNA compared to the DMSO-treated controls (Fig. 6k). In sum, taking into account the potent effect of MNK2-interfering drugs on β-cell differentiation in neonatal pig islets and human ductal organoids as well as the intriguing expression pattern of MNK2 in humans, further investigation of the translational potential of this class of drugs is warranted.

## Discussion

In the current study, we identified the molecular target of CID661578 as MNK2. The MNK2–CID661578 interaction potently induced β-cell regeneration from a pancreatic ductal cell origin and was sufficient to improve glucose control in both larval and adult diabetic zebrafish models. CID661578 was the only hit from our previous chemical screen that did not have a known target^4^. An important step in the drug discovery process is the identification of protein interactors and target engagement in vivo. Here, we used the YChemH system and identified MNK2 as one of the molecular targets of CID661578. Additionally, we used a combination of polysome profiling and in vitro biochemical experiments to shed light on the molecular mechanism of action of CID661578 in pancreatic ductal cells.

The neogenesis of β-cells from pancreatic ductal cells has been previously observed in the zebrafish model and has now been accepted as an endogenous path for β-cell regeneration^7,25–27^. However, whether a similar pancreatic progenitor population resides within the ductal cell compartment in adult mammalian models remains unclear. Different lineage tracing methods in mouse models have yielded various results regarding the contribution of ductal cells to β-cell neogenesis^8,28–38^. This controversy highlights the need to study ductal cells and their progenitor potential in the zebrafish model to identify new effectors and markers that could be translated to mammalian systems. Interestingly, CID661578 treatment also increased the formation of new β-cells from intraislet ductal cells in cultures of neonatal pig islet aggregates and stimulated *INS* expression in ductal-derived human organoids. These results demonstrated that the pathway could also be exploited to increase the differentiation of β-cells from a ductal source in mammalian systems.

We also observed that the Mnk2b–CID661578 interaction potently lowered glucose levels in zebrafish, suggested to be due to a combination of increased β-cell regeneration and protein synthesis (one of the most energy-consuming processes in the cell). This was supported by the fact that glucose lowering also occurred during homeostasis following chemical treatment, suggesting that increased glucose consumption is possibly the fuel for the increased protein synthesis. Interestingly, a recent report has linked analogs of cercosporamide to a glucose-lowering effect in mice^39^. Furthermore, *Mnk1*- and *Mnk2*-knockout mice exhibit beneficial metabolic outcomes when challenged with a high-fat diet^40,41^. These data are consistent with our observations and open new avenues for exploiting MNK2 in metabolic diseases.

During the course of our study, we performed metabolomics to assess the effects of CID661578 treatment in vivo, which highlighted global changes related to glucose metabolism and protein synthesis. We observed that the chemical treatment increased protein synthesis in vivo, an effect that was most profound in the ductal cell population. Hypertranslation as a mechanism that governs stem cell differentiation has recently been demonstrated for cell types belonging to a few different organs^42–45^. Our study expands this concept to the pancreas and to a regenerative setting in vivo, suggesting that targeting initiation of translation could represent a conserved process stimulating differentiation and regeneration in multiple systems.

MNKs belong to the MAPK interacting protein kinase family and were identified in screens for interactors of the ERK and p38 MAP kinases^46,47^. MNKs primarily phosphorylate eIF4E and thereby have a context-dependent role in protein synthesis^19^. However, although our data indicated that CID661578 treatment increased protein synthesis, it did not affect the kinase activity of MNK2 in vitro. Instead, we observed that CID661578 increased the interaction between mRNAs, the cap-binding protein eIF4E and the scaffold protein eIF4G, which also binds to MNK1/MNK2 (ref. ^13^), together with an increase in phospho-eIF4E levels. Notably, previous studies in, for example, cancer and immune cells using cercosporamide observed reduced phosphorylation of eIF4E^16,48^. This discrepancy could be attributed to differential activities and upstream regulation of the MNKs. MNK2 contributes to a basal level of phospho-eIF4E, while the activity of MNK1 can be potentiated by upstream signaling to increase phospho-eIF4E in the absence of MNK2 (ref. ^49^). Consistently, reduced expression of MNK2 was previously reported to be associated with increased levels of MNK1 (ref. ^50^). Nevertheless, our studies using pan-MNK inhibitors support that the increased phospho-eIF4E following CID661578 depends on hyperactive MNK1. Our interpretation is that β-cell regeneration is increased when MNK2 is inhibited but is unaffected when both MNK1 and MNK2 are inhibited. Therefore, deleting/interfering with the less efficient kinase might open up for the more efficient kinase, leading to a net increase in phospho-eIF4E. Additionally, we observed that CID661578 treatment drastically decreases the binding of MNK2 to eIF4G, resulting in increased interaction of eIF4E and eIF4G at the mRNA cap. Further, our polysome profiling analysis uncovered a total of 369 common translationally regulated mRNAs following treatment with CID661578 and cercosporamide. Coupled with the observed increase in phospho-eIF4E, this is one of the most extensive signatures of the effect of the phospho-eIF4E modification on the translatome described to date.

The zebrafish model has emerged as a powerful system for coupling large-scale screens with desired phenotypic outcomes in vivo. The ability to screen in vivo rather than using in vitro culture systems offers the advantage of performing chemical screening in a setting where all of the tissues are present and can interact. Here, we report the identification of Mnk2 as the molecular target of CID661578, the most striking hit from a chemical screen for drivers of β-cell regeneration. Our results identified a previously unknown role for Mnk2 in β-cell neogenesis from cells residing within the ductal cell compartment of the pancreas, thereby paving the way for an alternative path to stimulate β-cell neogenesis, and hence regeneration, for the management of diabetes.

## Methods

### Zebrafish transgenic lines and experimental procedures

Zebrafish experiments were conducted in compliance with local guidelines and our approved Swedish ethical permit for the usage of animals. Previously generated transgenic lines used in this study included *Tg(ins:flag-NTR)*^*s950*^, *Tg(ins:GFP)*^*zf5*^, *Tg(tp1:H2BmCherry)*^*s939*^, *Tg(mnx1:GFP)*^*ml2*^, *Tg(tp1:GFP)*^*um14*^, *Tg(gcga:GFP)*^*ia1*^, *Tg(sst2:dsRed2)*^*gz19*^, *Tg(ins:kaede)*^*s949*^, *Tg(ins:CFP-NTR)*^*s892*^, *Tg(tp1:creER*^*T2*^*)*^*s951*^ and *Tg(-3.5ubb:loxP-EGFP-loxP-mCherry)*^*cz1701*^ (referred to as *ubi:switch*). To avoid confusion, the co-integrated *Tg(ins:H2BGFP*; *ins:dsRed)*^*s960*^ line is referred to as *ins:dsRed* or *ins:H2BGFP* when only one fluorescent protein was visualized. We also independently integrated the *ins:H2BGFP* plasmid to generate *Tg(ins:H2BGFP)*^*KI112*^. As part of this work, we generated a new mutant termed *mknk2b*^*KI125*^ and an overexpressing zebrafish line termed *Tg(tp1:mknk2b)*^*KI113*^. The AB strain was used for experiments with wild-type zebrafish. The morpholinos targeted against *mknk2b*, *drl or acin1b* were synthesized by Gene Tools and had the following sequences: *mknk2b* MO (5′-AATACAATAACTTACCAGGTCGGGC-3′), *drl* MO (5′-AGAACAGACACCAACCTCTTTGTCC-3′) and *acin1b* MO (5′-AGTAATGATGTTCTTACTGTGACAT-3′). Four nanograms of the morpholinos was injected into the one-cell stage of zebrafish embryos (in parallel with a random morpholino control). For morpholino knockdown validation, RNA was extracted from a pool of 10 embryos using a Quick-RNA Microprep kit (ZYMO Research), followed by cDNA synthesis with a High Capacity cDNA reverse transcription kit (Applied Biosystems) and PCR amplification using DreamTaq PCR master mix (Thermo Fischer Scientific); the products of the PCR were run on a 1% agarose gel. The following primers were used for amplification: *mknk2b*, forward primer 5′-AGTTACCGGATTCCATCGCTCT-3′, reverse primer 5′-TTTGGAATCGGGGATGTCGAT-3′; *acin1b* forward primer 5′-CAGCTTTCTCCTCCTCGTGG-3′, reverse primer 5′-TCCTGGTCACTGAAGTCCAC-3′; *drl*, forward primer 5′-CGACTCACTCAAAACACAGAATC-3′, reverse primer 5′-ATGTGAATCTGAAGGTGCGC-3′; *eef1a1l1*, forward primer 5′-GTGCTGTGCTGATTGTTGCT-3′, reverse primer 5′-TGTATGCGCTGACTTCCTTG-3′. To generate zebrafish lines overexpressing zebrafish *mknk2b* and human *MKNK2*, cDNA of the genes was amplified from a pool of zebrafish at different developmental stages for *mknk2b* and from PANC-1 cDNA for *MKNK2*. The sequences were cloned into the middle entry vector pDON221 of the Gateway system, and three-way recombination was used to generate the final vector with the 5′ *tp1* promoter sequence and 3′ poly(A) sequence cloned into pDestTol2CG2. Mosaic overexpression was induced by injecting 20 pg of the vector together with 20 pg of transposase mRNA into one-cell-stage embryos. A stable transgenic line, named *Tg(tp1:mknk2b)*^*KI113*^, was generated for the constitutive overexpression of *mknk2b*.

For CRISPR–Cas9 mutagenesis, a suitable guide RNA sequence targeting the N-terminal part of the *mknk2b* gene was designed using the CHOPCHOP web tool (https://chopchop.cbu.uib.no), resulting in the sequence 5′-CAATAACTTACCAGGTCGGGCGG-3′. Then, IDT’s Alt-R CRISPR–Cas9 system was used to create the mutation. Briefly, equal volumes of the custom-made Alt-R CRISPR RNA (crRNA) with a sequence of 5′-CAAUAACUUACCAGGUCGGGGUUUUAGAGCUAUGCU-3′ was annealed with the universal Alt-R *trans*-activating crRNA (tracrRNA) sequence (IDT) by incubating the solution for 3 min at 95 °C and cooling at room temperature for 15 min to a final concentration of tracrRNA:crRNA of 10 µM. Then, the tracrRNA:crRNA was incubated with the same concentration of Cas9 protein (IDT) at 37 °C for 10 min, and 1 nl of the mixture was injected in the one-cell stage to generate *mknk2b*-mutant zebrafish. Mutagenesis was confirmed in pooled injected embryos after DNA extraction and qPCR followed by melt curve analysis using the following primers: forward primer 5′-AGGATCCCATCTCCTTGAATCT-3′ and reverse primer 5′-CACCCACAGGAAATAGCTTGAT-3′. An identified founder line that had germline transmission of a 4-base pair deletion (5′-CGAC-3′) at the end of exon 3 was used for all experiments using *mknk2b* mutants. Genotyping of mutants was done by qPCR with melt curve analysis using the QuantStudio V1.2.4 software.

Chemicals were added to the E3 medium for larvae or facility water for adult zebrafish to a final concentration of 2 or 10 µM CID661578 (as specified in the figure legends), 2 or 10 µM CID661578.6 (OnTarget Chemistry), 500 nM cercosporamide (Tocris Bioscience), 800 nM 4EGI-1 (Tocris Bioscience) and 10 µM eFT508 (MedChemExpress). EdU was added to the E3 medium to a final concentration of 5 mM together with HEPES (10 mM) and developed using the Click-iT EdU Alexa Fluor 647 kit (Thermo Fischer Scientific). OPP was added to E3 medium to a final concentration of 100 µM and developed using a Click-iT Plus OPP Alexa Fluor 647 Protein Synthesis Assay kit (Thermo Fischer Scientific). 4-Hydroxytamoxifen was added to the E3 medium to a final concentration of 5 µM for 24 h.

The ablation of β-cells in the *Tg(ins:flag-NTR)* or *Tg(ins:CFP-NTR)* line was performed by incubating the zebrafish for 24 h with 1 mM (juvenile and adult) or 10 mM (larvae) MTZ (Sigma-Aldrich) diluted in 1% DMSO (VWR) in facility water (juvenile and adult) or an E3 solution supplemented with 0.2 mM 1‐phenyl‐2‐thiourea (larvae; Acros Organics).

We estimated glycemia in larvae by measuring free glucose that had not been intracellularly phosphorylated by hexokinases using a Glucose Colorimetric/Fluorometric Assay kit (BioVision) in pools of four larvae for each time point and condition. Blood glucose measurements in adult zebrafish were performed using a standard glucometer (Freestyle, Abbott). Fish were fasted for 4 h, anesthetized in tricaine (Sigma-Aldrich) and decapitated for blood glucose measurements.

### Immunofluorescence and confocal analysis

Immunofluorescence staining and confocal analysis of larvae and 1-month-old fish were performed as previously described^27^. Primary antibodies were used against green fluorescent protein (GFP; to amplify the GFP signal, 1:500; Aves Labs, GFP-1020), glucagon (1:200; Sigma, G6254), insulin (1:100; custom made by Cambridge Research Biochemicals) and tdTomato (1:500; MYBioSource, MBS448092). For experiments in juveniles, the insulin:GFP^+^ area was measured on a flattened projection (average intensity). For the OPP intensity measurements, all larvae were imaged using the same parameters on a confocal microscope. Quantification was performed using the Fiji parameter (mean gray value). The mean gray value was measured from eight tp1:GFP^+^ cells around the islet from a single plane for each larva using the same area as a reference for each cell, and the average of the mean gray value of the eight cells was calculated. All images were acquired with LAS X (v3.5.5.19976) software. The contrast was adjusted for visualization purposes in some experiments, in which case the same adjustments were made for all displayed images from the same experiment. The original unmodified pictures were used for analysis.

### Chemical synthesis of CID661578 analogs

A detailed report that includes the steps used to synthesize all the analogs described in this study is provided in Supplementary Note 1.

### Yeast chemical hybrid screen

The YChemH screen is based on the same principle as the classical yeast two-hybrid system. Synthesis of the chemical probe for the screen was performed by coupling a terminal carboxylic acid and the trimethoprim-PEG5-NH2 building block to an amide group of CID661578.6 (OnTarget Chemistry). The chemical probe was used as the bait and screened against two cDNA libraries in yeast, one from zebrafish embryos (18–20 h after fertilization) and the other from human islets (Hybrigenics). Survival of the yeast on medium lacking histidine was used to identify positive clones in the screen. All histidine^+^ colonies were collected and screened for false positives, leading to 55 and 85 final positive clones in the zebrafish embryo and human islet libraries, respectively. The clones were sequenced to identify the corresponding prey, and a confidence score (A–E) was ascribed to each interaction based on two different levels of analysis. The confidence score was calculated as previously described^51^. Briefly, a local score considers the redundancy and independence of prey fragments together with the distribution of reading frames and stop codons in overlapping fragments. Then, a global score is calculated based on the interactions observed in all previous screens performed using the same library. The global score shows the probability of a hit being false positive. A hits were categorized as the most confident prey proteins, and E hits were the least probable. The full hit list report for both cDNA libraries is provided in Supplementary Note 2.

For the zebrafish Mnk2b binding experiments, full-length or truncated *mknk2b* (corresponding to the fragment of the human *MKNK2* identified in the original screen) was cloned into the prey vector. Two different hook vectors were used to validate the binding of zebrafish Mnk2b to the bait: 1-N-LexA-eDHFR-C (original hook vector) and 2-N-eDHFR-LexA-C (reverse order hook vector).

### Metabolomics

Metabolites were extracted from pools of 10 zebrafish larvae at 5 d.p.f. using a methanol-based extraction, and metabolite analysis was performed using liquid chromatography coupled with high-resolution mass spectrometry, as recently described in detail for zebrafish^27^. Xcalibur software was used for mass spectrometer data collection, and Sieve 2.2 was used for the chromatographic peak alignment. Differential regulation was examined based on *t*-test analysis in the Morpheus tool (https://software.broadinstitute.org/morpheus). Pathway analysis of the differentially regulated metabolites was performed using MetaboAnalyst 4.0 (ref. ^52^).

### Cell culture, immunofluorescence and m^7^GTP pulldown experiments

COLO 320HSR cells were obtained from ATCC and cultured with RPMI 1640 supplemented with GlutaMAX, penicillin/streptomycin (Pen/Strep) and 10% fetal bovine serum. PANC-1 cells were obtained from ATCC and cultured with DMEM supplemented with GlutaMAX, Pen/Strep and 10% fetal bovine serum. For m^7^GTP pulldown experiments, 24 h before treatment, cells were plated with DMSO, 40 µM CID661578.6, 40 µM 4EGI-1 or 40 µM cercosporamide for 6 h. The m^7^GTP pulldown was performed using similar amounts of protein lysates, which were quantified using a Pierce BCA Protein Assay kit (Thermo Fischer Scientific). Immobilized γ-aminophenyl-m^7^GTP (C_10_-spacer) beads were purchased from Jena Bioscience. For the assessment of eIF4E phosphorylation, PANC-1 cells were treated for 6 h with DMSO, 40 µM CID661578, 40 µM cercosporamide, 40 µM CGP57380 (Tocris Bioscience) or 40 µM eFT508 (MedChemExpress). Twenty-five microliters of immobilized γ-aminophenyl-m^7^GTP (C_10_-spacer) beads was incubated with the protein lysates for 1 h at 4 °C. Following incubation, the beads were washed three times with NET buffer (50 mM Tris-HCl (pH 7.4), 150 mM NaCl, 1 mM EDTA, 0.1% Triton X-100 and one tablet of protease inhibitors), and proteins were eluted from the beads with loading buffer (4× Laemmli sample buffer, Bio-Rad).

For the immunoprecipitation experiments, PANC-1 cells were plated in 10-cm dishes and transfected using the DharmaFECT Duo transfection reagent (Active Motif) with 2 µg of the MNK2–FLAG plasmid, obtained from Origene (NM_199054, RC216704). Cells were lysed with Pierce IP lysis buffer (87788), and 1 mg of protein was incubated for the immunoprecipitation with anti-FLAG (Sigma-Aldrich, 6 µg, F3165) overnight. As a negative control for the immunoprecipitation assay, we used the lysis buffer alone incubated overnight with anti-FLAG. The following day, the samples were incubated for 1 h with 30 µl of A/G magnetic beads (Thermo Fisher Scientific, 88803) and washed four times with NET-2 buffer (50 mM Tris-HCl (pH 7.4), 150 mM NaCl, 1 mM MgCl_2_, 0.5% Nonidet P-40 and one tablet of protease inhibitors), and proteins were eluted from the beads with loading buffer (4× Laemmli sample buffer, Bio-Rad).

For western blotting, after separating proteins on 4–15% gradient gels (Bio-Rad), proteins were transferred to membranes that were then blocked with 5% milk powder. Antibodies against the following proteins were used: eIF4G (1:2,000; Cell Signaling Technologies, 2498), eIF4E (1:2,000; Cell Signaling Technologies, 9742), MNK2 (1:1,000; Sigma-Aldrich, SAB2101483), β-actin (1:5,000; Sigma-Aldrich, A5441, clone AC-15), phospho-eIF4E (Ser 209; 1:1,000; Cell Signaling Technologies, 9741), anti-rabbit coupled to horseradish peroxidase (1:5,000; Thermo Fischer Scientific, 31460) and anti-mouse coupled to horseradish peroxidase (1:5,000; Jackson ImmunoResearch, AB_2307347).

For the reticulocyte m^7^GTP pulldown assay, untreated rabbit reticulocyte lysates (Promega) were incubated with the specified concentrations of CID661578.6 for 1 h at 30 °C followed by m^7^GTP pulldown and western blotting, as described above.

### Polysome profiling

To isolate fractions of efficiently translated mRNAs, polysome profiling, using an optimized sucrose gradient, was performed using a recently described optimized sucrose gradient^53^. Briefly, 4 × 10^6^ PANC-1 cells were seeded in 15-cm plates 24 h before treatment. Cells were treated with cercosporamide (5 μM), CID661578 (40 μM) or DMSO for 6 h and lysed in a hypotonic lysis buffer. Aliquots of cytosolic lysate were collected from each sample for isolation of total cytosolic RNA. The remaining lysates were layered onto optimized sucrose density gradients (5%:34%:55% (wt/vol)), and samples were centrifuged at 4 °C at 35,000 r.p.m. for 2 h followed by fractionation. Fractions containing mRNAs bound to more than three ribosomes were collected in TRIzol reagent (Thermo Fischer Scientific) and pooled, allowing for isolation of efficiently translated polysome-associated mRNA.

RNA extraction was performed using the TRIzol reagent protocol (Thermo Fischer Scientific) followed by additional purification using an RNAeasy MinElute Cleanup kit (Qiagen). RNA quality was assessed using a Bioanalyzer 2100 with an RNA 6000 Nano kit (Agilent). Smartseq2 sequencing libraries were prepared as previously described^54^ using 10 ng of mRNA as input. Libraries were prepared for total cytosolic and polysome-associated fractions from four biological replicates of cells treated with cercosporamide, CID661578 or DMSO. Libraries were pooled and sequenced on an Illumina NovaSeq 6000 platform using a 50-base pair paired-end setup.

RNA sequencing read quality was evaluated using MultiQC (1.7). Adapters and reads mapping to ribosomal RNA were removed using BBDuk (36.59) from the BBTools suite (http://jgi.doe.gov/data-and-tools/bb-tools/) before alignment to hg38 using HISAT2 (2.1.0) with default settings. Reads were summarized using RSubread (2.6.4) featureCounts with default settings and RefSeq gene definitions^55^. Genes with zero counts in at least one sample were removed, and the data were trimmed mean of the M values log_2_ normalized. Changes in mRNA abundance (that is, congruent modulation in total and polysome-associated mRNA; downstream of altered transcription or mRNA stability), translation efficiency (that is, changes in levels of polysome-associated mRNA not paralleled by corresponding alterations in total mRNA) and buffering (that is, changes in total mRNA level but not polysome association and therefore predicted to result in unchanged protein levels) between cells treated with cercosporamide or CID661578 and DMSO were assessed using the anota2seq (1.14.0) algorithm^56^. The following thresholds were applied within the anota2seqRun function: maxPAdj = 0.15; deltaP = log_2_ (1); deltaT = log_2_ (1); deltaPT = log_2_ (1.2); deltaTP = log_2_ (1.2); maxSlopeTranslation = 2; minSlopeTranslation = −1; minSlopeBuffering = −2; maxSlopeBuffering = 1. Replicate was included in the anota2seq model using the ‘batchVec’ parameter to account for batch effects. Genes were classified according to their mode of regulation (mRNA abundance or translation) using the anota2seqRegModes function.

GO analysis was performed using the ClueGO plug-in (2.5.8) within CytoScape (3.8.2; https://cytoscape.org). Analyses of 5′ UTRs were based on the RefSeq curated sequences. Differences in 5′ UTR length, GC content and length-corrected fold energy between mRNA in each regulatory mode were assessed using a two-sided Mann–Whitney test.

### Single-cell RNA-seq analysis

Single-cell RNA-seq data of adult zebrafish used for the analysis were downloaded from the Gene Expression Omnibus (GEO) under accession number GSE106121 and sample number GSM3032164 (ref. ^17^). The unique molecular identifier counts matrix was imported into R and processed using the Seurat R package version 3.5.1 (ref. ^57^). Low-quality cells with detected gene numbers less than 450 or higher than 2,200 along with mitochondrial genes were removed before downstream analysis. Subsequently, we performed principal component analysis and selected the top 21 significant principal components for dimensional reduction. A graph-based clustering method (Louvain) was used to cluster cells with a resolution of 0.5. Finally, we used the uniform manifold approximation and projection (UMAP; part of the Seurat 3.5.1 package algorithm) to display the relationships within and between different clusters. Enrichment analysis of differentially expressed genes was performed and visualized using the clusterProfiler package (3.10.1).

### Dose–response assessment and in vitro kinase screen

Dose–response assessments and the in vitro kinase screen were performed by the International Centre for Kinase Profiling. All kinase assays were performed using a Multidrop 384 instrument at room temperature in a total assay volume of 25.5 μl. DMSO controls or acid blanks and 15 μl of the enzyme mix containing enzyme and peptide/protein substrate in buffer were added to plates containing 0.5 μl of compounds. The compounds were preincubated in the presence of the enzyme and peptide/protein substrate for 5 min before initiation of the reaction by addition of 10 μl of ATP (final concentration selected for each kinase at 5, 20 or 50 μM). The reactions were incubated for 30 min at room temperature before termination by the addition of 5 μl of orthophosphoric acid. The assay plates were collected onto P81 Unifilter plates by a PerkinElmer Harvester and air dried. The dry Unifilter plates were then sealed after the addition of MicroScint O and analyzed in PerkinElmer Topcount scintillation counters.

### Pig islet aggregates and immunofluorescence experiments

All procedures involving pigs were performed according to the guidelines established by the Canadian Council on Animal Care. Donor pancreases were surgically removed from neonatal piglets of either sex (Swine Research and Technology Center, University of Alberta). Neonatal porcine islets were isolated and maintained in Ham’s-F10 tissue culture medium (Sigma-Aldrich), as previously described^58^. For clarification, because neonatal pigs do not contain intact mature islets structures, the term neonatal porcine islets refers to aggregates of endocrine and exocrine tissue generated in culture following digestion of the pancreas. At the third day of cultivation, the incubation medium was switched to Ham’s-F10 medium (without IBMX) supplemented with either 1 µM CID661578 (Sigma-Aldrich) or 1 µM cercosporamide (Tocris). The islets were treated for 5 d, and the medium was replaced with identical fresh medium every 48 h. Samples were collected from each condition, and immunohistochemical staining was performed as described previously^59^. Antibodies against the following proteins were used to stain the pig sections: insulin (1:5, DAKO, IR002), CK7 (3:100, DAKO, clone OV/TL 12/30) and MNK2 (1:200, Sigma-Aldrich, SAB2101483). Human pancreatic sections were stained using the same protocol as described for the porcine sections. All images were acquired with NIS-Elements (version 4.30) software. Human tissues were kindly provided by the Alberta Diabetes Institutes Islet Core, and ethical approval for the use of human samples was obtained from the University of Alberta’s Human Research Ethics Board, protocol PRO00001416. Informed written consent was provided at the institutions where the organs were collected.

### Human pancreatic ductal organoid culture

Human pancreatic exocrine tissue, obtained from the discarded fraction after human islet purifications using the Ricordi method from cadaveric organ donors with informed written consent, was processed to isolate ductal fragments and generate organoid cultures. Ethical approval for processing pancreatic samples from deidentified organ donors was granted by the Clinical Research Ethics Committee of Hospital de Bellvitge (PR030/22). Ductal fragments were embedded in GFR Matrigel and cultured in human organoid expansion medium^60^. After three to four passages, organoid expansion medium was replaced by a basic medium containing Advanced DMEM/F12, ITS-X (1×), heparin (0.1 mg ml^–1^), nicotinamide (0.1 mM), *N*-acetylcysteine (0.25 mM), FGF10 (0.1 µg ml^–1^), N2 supplement (1×) and B27 (1×), and organoids were treated with DMSO, CID661578 (50 µM) or cercosporamide (100 µM). The medium was renewed every other day, and the organoids were cultured under these conditions for 8 d. Following treatment, RNA was isolated from ductal organoids using the RNeasy minikit (Qiagen) followed by DNase I treatment (Invitrogen). The RNA was reverse transcribed with SuperScript III reverse transcriptase (Roche) and random hexamers, and qPCR was performed on a 7900 real-time PCR system (Applied Biosystems) using Power SYBR green (Applied Biosystems). Primers used for *TBP* included forward primer 5′-ATCCCTCCCCCATGACTCCCATG-3′ and reverse primer 5′-ATGATTACCGCAGGAAACCGC-3′, and primers used for *INS* included forward primer 5′-GCAGCCTTTGTGAACCAACA-3′ and reverse primer 5′-TTCCCCGCACACTAGGTAGAGA-3′.

### Statistical analysis

All data are presented as mean values ± s.e.m. Statistical analyses were performed using GraphPad Prism 8.0/9.0, except for the metabolomics analysis (see Metabolomics). All respective *P* values are presented in the figure legends, and *P* values ≤ 0.05 are considered significant. The used statistical tests are listed in the respective figure legends.

### Reporting summary

Further information on research design is available in the Nature Research Reporting Summary linked to this article.

## Online content

Any methods, additional references, Nature Research reporting summaries, source data, extended data, supplementary information, acknowledgements, peer review information; details of author contributions and competing interests; and statements of data and code availability are available at 10.1038/s41589-022-01047-x.

## Supplementary information

Supplementary Information Supplementary Notes 1 and 2.

Reporting Summary

Supplementary Data 1 Anota2seq results for cercosporamide versus DMSO.

Supplementary Data 2 Anota2seq results for CID661578 versus DMSO.

Supplementary Data 3 Commonly translationally regulated genes between CID661578 and cercosporamide.

## Data Availability

The raw metabolomics data were uploaded to Metabolomics Workbench with the study ID ST002119 (10.21228/M80D9F). The raw polysome profiling data were uploaded to GEO with the accession number GSE200477. Expression of *mknk2b* in zebrafish was assessed using data downloaded from GEO under the accession number GSE106121 and sample number GSM3032164. The rest of the data are included in the current manuscript. The datasets generated during the current study are available from the corresponding author on reasonable request. Source data are provided with this paper.

## Source data

Source Data Fig. 2 Statistical source data for Fig. 2.
Source Data Fig. 3 Statistical source data for Fig. 3.
Source Data Fig. 4 Statistical source data for Fig. 4.
Source Data Fig. 5 Statistical source data for Fig. 5.
Source Data Fig. 5 Uncropped raw western blot images for Fig. 5.
Source Data Fig. 6 Statistical source data for Fig. 6.
Source Data Extended Data Fig. 1 Statistical source data for Extended Data Fig. 1.
Source Data Extended Data Fig. 2 Statistical source data for Extended Data Fig. 2.
Source Data Extended Data Fig. 3 Statistical source data for Extended Data Fig. 3.
Source Data Extended Data Fig. 4 Statistical source data for Extended Data Fig. 4.
Source Data Extended Data Fig. 6 Statistical source data for Extended Data Fig. 6.
Source Data Extended Data Fig. 7 Statistical source data for Extended Data Fig. 7.
Source Data Extended Data Fig. 7 Uncropped raw agarose gel images for Extended Data Fig. 7.
Source Data Extended Data Fig. 9 Statistical source data for Extended Data Fig. 9.
Source Data Extended Data Fig. 10 Statistical source data for Extended Data Fig. 10.
