# Supplementary material for: MNK2 deficiency potentiates β-cell regeneration via translational regulation
Source: Nat Chem Biol. 2022 Jun 13;18(9):942–53. doi: 10.1038/s41589-022-01047-x (PMC7613404; doi:10.1038/s41589-022-01047-x)
Supplement: Supplementary file 1 — Supplementary Notes 1 and 2. [file 41589_2022_1047_MOESM1_ESM.pdf]

nature portfolio

https://doi.org/10.1038/s41589-022-01047-x

Supplementary information

MNK2 deficiency potentiates β-cell regeneration via translational regulation

In the format provided by the
authors and unedited

Supplementary Note 1

Compilation of Experimental Protocols of the Synthesis of 7-hydroxy-3-methyl-5-oxo-2,3-dihydro-1*H*,5*H*-pyrido[3,2,1-ij]quinoline-6-carboxylic acid Derivatives

Chemical synthesis information for CID661578.6 (**2**), CID661578.6-derived bait (**3**) and analogues in Extended Data Figure 1

Content

1. INTRODUCTION 3
2. GENERAL METHODS 4
3. SYNTHESIS OF AMIDES WITH THE 7-HYDROXY-3-METHYL-5-OXO-2,3-DIHYDRO-1*H*,5*H*-PYRIDO[3,2,1-IJ]QUINOLINE-6-CARBOXAMIDE CORES 4
3.1 QUINOLINE REDUCTION 4
3.2 CYCLIZATION 5
3.3 ESTER HYDROLYSIS 6
3.4 CONCOMINANT ETHER AND ESTER CLEAVAGE 7
3.5 AMIDE COUPLINGS 8
4. SYNTHESIS OF TRIMETHOPRIM-PEG$_6$-*N*-(3-(2-AMINO-2-OXOETHYL)BENZYL)-7-HYDROXY-3-METHYL-5-OXO-2,3-DIHYDRO-1*H*,5*H*-PYRIDO[3,2,1-IJ]QUINOLINE-6-CARBOXAMIDE 10
4.1 AMIDE COUPLING OF CORE MOLECULE WITH 3-(METHOXYCARBONYLMETHYL)BENZYLAMINE 10
4.2 ESTER HYDROLYSIS 11
4.3 AMIDE COUPLING WITH H_2N-PEG$_6$-TMP 11
4.4 CHROMATOGRAMS AND MASS-SPECTRA OF THE FINAL PRODUCT 12

1. Introduction

Karolinska Institutet's Department of Cell and Molecular Biology as represented by Assoc. Prof. Olov Andersson (the customer) tasked Recipharm with compiling experimental protocols for the synthesis of 7-hydroxy-3-methyl-5-oxo-2,3-dihydro-1*H*,5*H*-pyrido[3,2,1-ij]quinoline-6-carboxylic acid derivates.

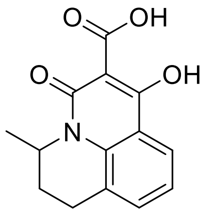

7-hydroxy-3-methyl-5-oxo-2,3-dihydro-1*H*,5*H*-pyrido[3,2,1-*ij*]quinoline-6-carboxylic acid core

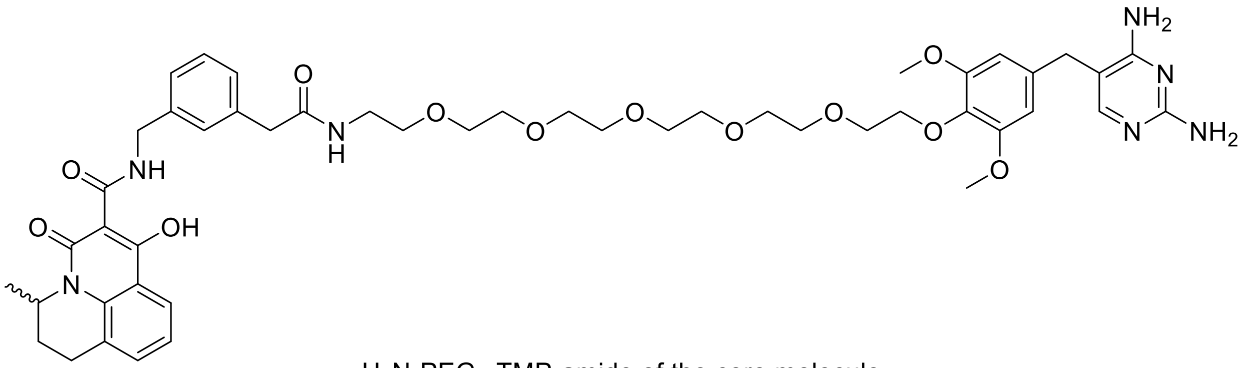

H_2N-PEG_6-TMP-amide of the core molecule

2. General Methods

All solvents were obtained from Chemtronica (Solna, Sweden) and were not purified or dried prior to use. H_2N-PEG_6-TMP was supplied by Hybrigenics SA (Evry, France). All other reagents were obtained from Sigma-Aldrich (Stockholm, Sweden). Reactions were monitored using an Agilent 1100 series Liquid Chromatograph/Mass Selective Detector (MSD) (Single Quadrupole) equipped with an electrospray interface, a UV diode array detector and an ACE3 C8 (3.0 x 50 mm) column with a gradient of acetonitrile (10→97%) in 0.1% aqueous 2,2,2-trifluroacetic acid over 3 min and a flow of 1 mL/min or an XBrdige C18 (3.0 x 50mm) column with a gradient of acetonitrile (5→97%) in 10mM aqueous ammonium bicarbonate. Purification by means of preparative liquid chromatography were performed using a Gilson HPLC System equipped with a model 119 UV/Vis detector, a series 306 binary pump, an FC204 fraction collector and an ACE3 C18-HL (250 x 21.2 mm) column with a gradient of acetonitrile in 0.1% aqueous 2,2,2-trifluroacetic acid over 10 min and a flow of 25 mL/min.

3. Synthesis of amides with the 7-Hydroxy-3-methyl-5-oxo-2,3-dihydro-1*H*,5*H*-pyrido[3,2,1-ij]quinoline-6-carboxamide cores

3.1 Quinoline reduction

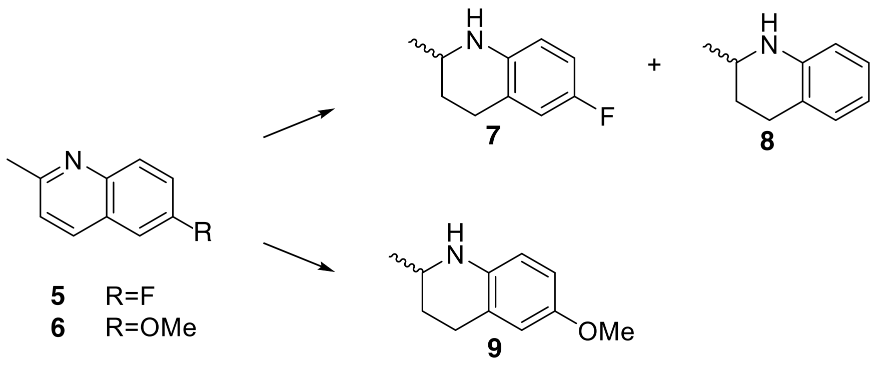

6-Fluoro-2-methyl-1,2,3,4-tetrahydroquinoline (7) and **2-methyl-1,2,3,4-tetrahydroquinoline (8)**

6-Fluoro-2-methylquinoline (**5**, 300mg, 1.86mmol, 1.0eq.) was weighed into a flame-dried 50mL round-bottom flask, followed by PtO_2 (42mg, 0.19mmol, 0.1eq.) and a stirbar. The flask was sealed with a septum, the atmosphere was evacuated and backfilled with nitrogen three times. MeOH (7.5mL) and TFA (0.14mL, 1.0eq.) were added by syringe and a hydrogen atmosphere was applied. The resulting suspension was stirred at room temperature overnight. After approximately 18 hours, the solids were removed with the help of a syringe filter and the latter was washed with MeOH (2.5mL). Incrementally and while stirring, aqueous NH_4HCO_3 (20mL, 50mM) was added to neutralize the acid, and the resulting suspension was extracted with EtOAc (3 x 20 mL). The organic layers were pooled, dried over sodium sulfate and the solvent was removed *in vacuo* to yield an oily residue, which was filtered through silica (heptane/EtOAc 4:1) to yield a mixture of **7** and **8** in a ratio of approximately 4:1 (153mg) as a light brown oil, which corresponds to a yield of approximately 51% when taking into account the different molecular weights of the products. The mixture of the two compounds was used in the subsequent step without further purification. MS/ESI positive ionization: m/z=166 $[M+H]^+$ (7) and m/z=148 $[M+H]^+$ (8).

6-Methoxy-2-methyl-1,2,3,4-tetrahydroquinoline (9)

An analogous procedure was used for the reduction of 300mg of 6-methoxy-2-methylquinoline (**6**) to **9**. The final filtration through silica was performed using DCM/MeOH/NEt_3 (19:1 + 0.1%) as the eluent. **9** was obtained as a light brown oil in 60% yield (183mg). The latter was used in the subsequent step without further purification. MS/ESI positive ionization: m/z=178 $[M+H]^+$.

3.2 Cyclization

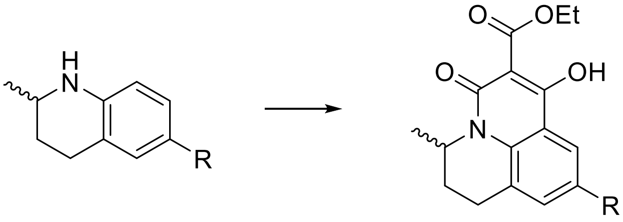

7 R=F
8 R=H
9 R=OMe

10 R=F
11 R=H
12 R=OMe

Ethyl 9-fluoro-7-hydroxy-3-methyl-5-oxo-2,3-dihydro-1H,5H-pyrido[3,2,1-ij]quinoline-6-carboxylate (10) and
ethyl 7-hydroxy-3-methyl-5-oxo-2,3-dihydro-1H,5H-pyrido[3,2,1-ij]quinoline-6-carboxylate (11)

An open, 15mL microwave tube was charged with triethyl methanetricarboxylate (0.21mL, 0.99mmol, 3.3eq.) and a stir bar. The vial was immersed into a steel block approximately halfway and heated to 225°C while stirring, causing the triester to gently reflux inside of the vial. A mixture of compounds **7** and **8** (approximately 4:1 ratio, 49mg, 0.30mmol, 1.0eq.) was added by micropipette. The resulting solution was stirred at 225°C for 30 minutes, whereupon it was allowed to attain room temperature. Aqueous Na_2CO_3 (10% w/w, 15mL) was added, the resulting mixture was heated to 80°C for 20 minutes and the solids were removed by filtration. Once the aqueous layer had obtained room temperature, activated charcoal (approx. 300mg) was added, the resulting suspension was stirred for 20 minutes, filtered and the solids were washed with hot water (approx. 50mL). While cooling on ice, the new aqueous layer was acidified with 4M aqueous HCl to precipitate a mixture of **10** and **11** in a ratio of approximately 4:1 (64mg) as an off-white solid, which corresponds to a yield of approximately 71% when taking into account the different molecular weights of the products. The mixture of the two compounds was used in the subsequent step without further purification. MS/ESI positive ionization: m/z=306 $[M+H]^+$ (10) and m/z=288 $[M+H]^+$ (11).

Ethyl 7-hydroxy-9-methoxy-3-methyl-5-oxo-2,3-dihydro-1H,5H-pyrido[3,2,1-ij]quinoline-6-carboxylate (12)

An analogous procedure was used for the conversion of 101mg of 6-methoxy-2-methyl-1,2,3,4-tetrahydroquinoline (**9**) to **12**, which was obtained as an off-white solid in 56% yield (102mg) after an additional recrystallization from acetonitrile. The product was used in the subsequent step without further purification. MS/ESI positive ionization: m/z=318 $[M+H]^+$.

3.3 Ester hydrolysis

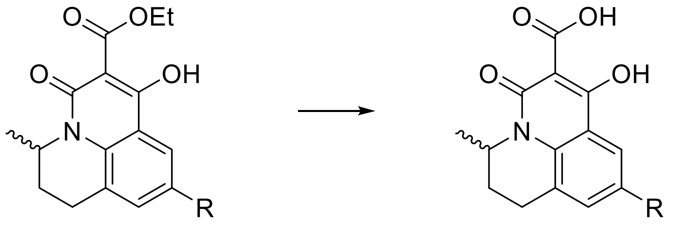

10 R=F
11 R=H
12 R=OMe

13 R=F
14 R=H
15 R=OMe

9-Fluoro-7-hydroxy-3-methyl-5-oxo-2,3-dihydro-1H,5H-pyrido[3,2,1-ij]quinoline-6-carboxylic acid (13) and
7-hydroxy-3-methyl-5-oxo-2,3-dihydro-1H,5H-pyrido[3,2,1-ij]quinoline-6-carboxylic acid (14)
A 5mL roundbottom flask was charged with a mixture of compounds **10** and **11** (approximately 4:1 ratio, 64mg, 0.21mmol), followed by a stir bar, glacial AcOH (2mL) and concentrated aqueous HCl (0.2mL, 35% w/w). A reflux condenser was installed and while stirring, the mixture was heated to 60°C, where it was kept overnight. After approximately 16 hours, the mixture was allowed cooled on ice for 20 minutes, and the solids were collected by filtration. The solids were washed with 1M HCl (1mL) and thereafter dried under fine vacuum overnight to obtain **13** and **14** in a ratio of approximately 4:1 (54 mg) as an off white solid, which corresponds to a yield of approximately 94% when taking into account the different molecular weights of the products. The mixture of the two compounds was used in the subsequent step without further purification. MS/ESI positive ionization: m/z=278 $[M+H]^+$ (13) and m/z=260 $[M+H]^+$ (14).

7-Hydroxy-9-methoxy-3-methyl-5-oxo-2,3-dihydro-1*H*,5*H*-pyrido[3,2,1-ij]quinoline-6-carboxylic acid (15)
An analogous procedure was used for the conversion of 102mg of ethyl 7-hydroxy-9-methoxy-3-methyl-5-oxo-2,3-dihydro-1H,5H-pyrido[3,2,1-ij]quinoline-6-carboxylate (**12**) to **15**, which was obtained as an off-white solid in 97% yield (90mg). The product was used in the subsequent step without further purification. MS/ESI positive ionization: m/z=290 $[M+H]^+$.

3.4 Concominant ether and ester cleavage

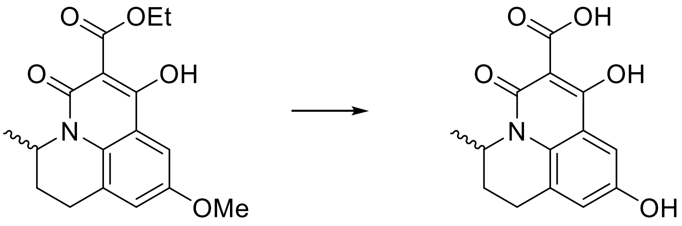

12 16

7,9-Dihydroxy-3-methyl-5-oxo-2,3-dihydro-1*H*,5*H*-pyrido[3,2,1-ij]quinoline-6-carboxylic acid (16)

Ethyl 7-hydroxy-9-methoxy-3-methyl-5-oxo-2,3-dihydro-1H,5H-pyrido[3,2,1-ij]quinoline-6-carboxylate (**12**, 50mg, 0.16mmol) was dissolved in hydrogen bromide in acetic acid (33% w/w, 5mL) inside of a 50mL roundbottom flask and the resulting solution was heated to reflux for 12 hours. The reflux condenser was then removed to allow for the evaporation of hydrogen bromide,[1] the volume of the resulting mixture was reduced to about 1mL using a rotoary evaporator and water (3mL) and MeCN (9mL) were added to yield a slightly brown solution. The latter was slowly concentrated in vacuo, until the desired product began to precipitate. The mixture was cooled on ice for one hour and the solids were collected by filtration. **16** was obtained in 55% yield (24mg) as a faintly brown solid. The product was used in the subsequent step without further purification. MS/ESI positive ionization: m/z=276 $[M+H]^+$.

[1] Due to the extreme corrosiveness of hydrogen bromide vapors, they were sucked off using a suction pump, where the gas was automatically diluted with water.

3.5 Amide Couplings

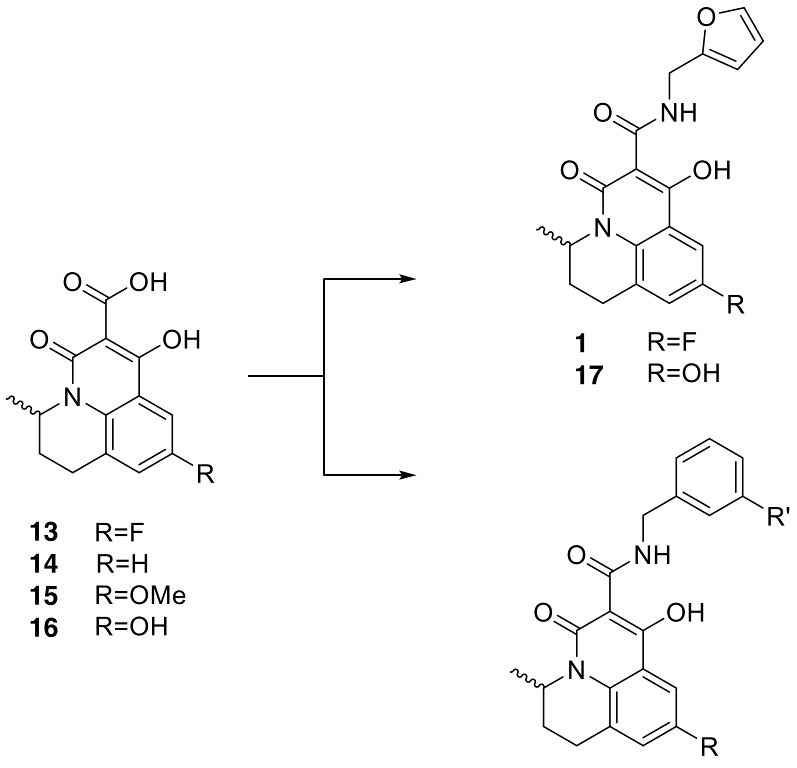

1 R=F
17 R=OH

13 R=F
14 R=H
15 R=OMe
16 R=OH

18 R=F, R'=OMe
19 R=H, R'=OMe
20 R=F, R'=H
2 R=H, R'=H
21 R=OMe, R'=OMe
22 R=OMe, R'=H
23 R=F, R'=OH

Amide couplings were performed in ethyl acetate, using propylphosphonic anhydride (T3P®) as the coupling reagent and triethylamine as a base. The synthesis of compounds **18** and **19** is exemplary.

9-Fluoro-7-hydroxy-*N*-(3-methoxybenzyl)-3-methyl-5-oxo-2,3-dihydro-1H,5H-pyrido[3,2,1-ij]quinoline-6-carboxamide (18) and
7-hydroxy-*N*-(3-methoxybenzyl)-3-methyl-5-oxo-2,3-dihydro-1H,5H-pyrido[3,2,1-ij]quinoline-6-carboxamide (19)

A 4mL vial was charged with a mixture of compounds **13** and **14** (approximately 4:1 ratio, 30mg, 0.11mmol). Added were EtOAc (0.9mL), followed by a stirbar and triethylamine (18µL, 0.12mmol, 1.1eq.). A solution of (3-methoxybenzyl)amine (40mg, 0.32mmol, 3.0eq.) in EtOAc (0.1mL) was added and finally, T3P-solution (≥50%w/w in EtOAc, 130µL, 0.22mmol, 2.0eq.) was added by micropipette. The resulting solution was stirred at room temperature over night. The resulting suspension was diluted with EtOAc (9mL), washed with water (5mL), and concentrated to dryness. The semi-solid residue was taken up in MeCN (3mL), aqueous TFA was added (0.5%, 1mL) and the resulting yellow solution was purified by preparative HPLC (60→80% MeCN in 0.5% aq. TFA, C18 column, ACE) to yield: **18** in 29% yield (17mg) and **19** in 10% yield (4mg), each as an off-white solid. MS/ESI positive ionization: m/z= 397 $[M+H]^+$ (18) and m/z=379 $[M+H]^+$ (19).

9-Fluoro-*N*-(furan-2-ylmethyl)-7-hydroxy-3-methyl-5-oxo-2,3-dihydro-1H,5H-pyrido[3,2,1-ij]quinoline-6-carboxamide (1)
20mg of **13** (contaminated with approx. 20% of 14) were converted to **1** by T3P-mediated amide coupling with furfurylamine. **1** was obtained in 34% yield (26mg) as an off-white solid. MS/ESI positive ionization: m/z= 357 $[M+H]^+$.

***N*-(furan-2-ylmethyl)-7,9-dihydroxy-3-methyl-5-oxo-2,3-dihydro-1*H*,5*H*-pyrido[3,2,1-ij]quinoline-6-carboxamide (17)**
107mg of **16** were converted to **17** by T3P-mediated amide coupling with furfurylamine. The reaction was noticably less clean owing perhaps to instability of the phenol-group in the presence of phosphonic anhydride. **17** was obtained in 5% yield (7mg) as an off-white solid. MS/ESI positive ionization: m/z= 355 $[M+H]^+$.

***N*-benzyl-9-fluoro-7-hydroxy-3-methyl-5-oxo-2,3-dihydro-1*H*,5*H*-pyrido[3,2,1-ij]quinoline-6-carboxamide (20)** and
***N*-benzyl-7-hydroxy-3-methyl-5-oxo-2,3-dihydro-1*H*,5*H*-pyrido[3,2,1-ij]quinoline-6-carboxamide (2)**
35mg of **13** and **14** were converted to **20** and **2** by T3P-mediated amide coupling with benzylamine. **20** was obtained in 21% yield (10mg) and **2** was obtained in 10% yield (5mg), each as an off-white solid. MS/ESI positive ionization: m/z= 367 $[M+H]^+$ (20) and m/z=349 $[M+H]^+$ (2).

7-Hydroxy-9-methoxy-*N*-(3-methoxybenzyl)-3-methyl-5-oxo-2,3-dihydro-1*H*,5*H*-pyrido[3,2,1-ij]quinoline-6-carboxamide (21)
44mg of **15** were converted to **21** by T3P-mediated amide coupling with (3-methoxybenzyl)amine. **21** was obtained in 60% yield (9mg) as an off-white solid. MS/ESI positive ionization: m/z=409 $[M+H]^+$.

***N*-benzyl-7-hydroxy-9-methoxy-3-methyl-5-oxo-2,3-dihydro-1*H*,5*H*-pyrido[3,2,1-ij]quinoline-6-carboxamide (22)**
47mg of **15** were converted to **22** by T3P-mediated amide coupling with benzylamine. **22** was obtained in 35% yield (21mg) as an off-white solid. MS/ESI positive ionization: m/z=379 $[M+H]^+$.

9-Fluoro-7-hydroxy-*N*-(3-hydroxybenzyl)-3-methyl-5-oxo-2,3-dihydro-1*H*,5*H*-pyrido[3,2,1-ij]quinoline-6-carboxamide (23)
40mg of **13** (contaminated with approx. 20% of 14) were converted to **23** by T3P-mediated amide coupling with (3-hydroxybenzyl)amine. The reaction was noticably less clean owing perhaps to instability of the phenol-group in the presence of phosphonic anhydride. A total of ten molar equivalents of amine were added, instead of the usual three. **23** was obtained in 5% yield (3mg) as an yellow syrup. MS/ESI positive ionization: m/z=383 $[M+H]^+$.

4. Synthesis of trimethoprim-PEG$_6$-*N*-(3-(2-amino-2-oxoethyl)benzyl)-7-hydroxy-3-methyl-5-oxo-2,3-dihydro-1*H*,5*H*-pyrido[3,2,1-ij]quinoline-6-carboxamide

4.1 Amide coupling of core molecule with 3-(Methoxycarbonylmethyl)benzylamine

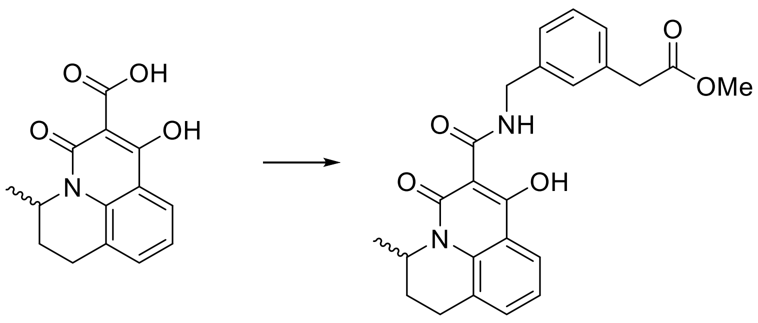

14 **24**

Methyl 2-(3-((7-hydroxy-3-methyl-5-oxo-2,3-dihydro-1H,5H-pyrido[3,2,1-ij]quinoline-6-carboxamido)methyl)phenyl)acetate (24)

A 100mL roundbottom flask was charged with **14** (503mg, 1.94mmol). Added were EtOAc (19mL), followed by a stirbar and triethylamine (292µL, 2.14mmol, 1.1eq.). A mixture consisting of 3-(methoxycarbonylmethyl)benzylamine hydrochloride (1.26g, 5.8mmol, 3.0eq.) and EtOAc (6mL) was added and finally, T3P-solution (≥50%w/w in EtOAc, 2.3mL, 3.88mmol, 2.0eq.) was added via syringe. The resulting solution was stirred at room temperature over night. The resulting suspension was diluted with EtOAc (25mL), washed with water (25mL), and concentrated to dryness. The residue was taken up in DMF (10mL) and dropwise, 0.1M HCl (10mL) was added to force the precipitation of product. The resulting suspension was cooled on ice for one hour, the solids were filtered and dried in vacuo overnight to yield **24** in 61% yield (498mg) as an off-white solid. The material was used in the subsequent step without further purification. MS/ESI positive ionization: m/z=421 $[M+H]^+$.

4.2 Ester hydrolysis

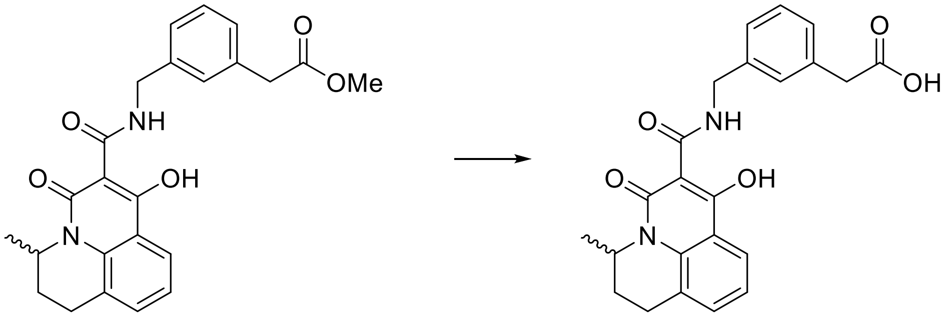

24 **25**

2-(3-((7-hydroxy-3-methyl-5-oxo-2,3-dihydro-1*H*,5*H*-pyrido[3,2,1-ij]quinoline-6 carboxamido)methyl)phenyl)acetic acid (25)

A 100mL roundbottom flask was charged with **24** (469mg, 1.11mmol) and MeCN was added (50 mL). The resulting solution was stirred at room temperature while 1M aq. NaOH (5mL) was added. The resulting mixture was stirred overnight, whereupon it was concentrated to approximately 10% of its initial volume in vacuo. Slowly and while stirring, 1M aq. HCl (10 mL)§ was added and the resulting aqueous phase was extracted with DCM (5 x 25 mL). The combined organic phases were dried over $MgSO_4$, filtered and the solvent was removed in vacuo to yield **25** as a colorless foam in 89% yield (401mg). MS/ESI positive ionization: m/z=407 $[M+H]^+$.

4.3 Amide coupling with H_2N-PEG_6-TMP

25

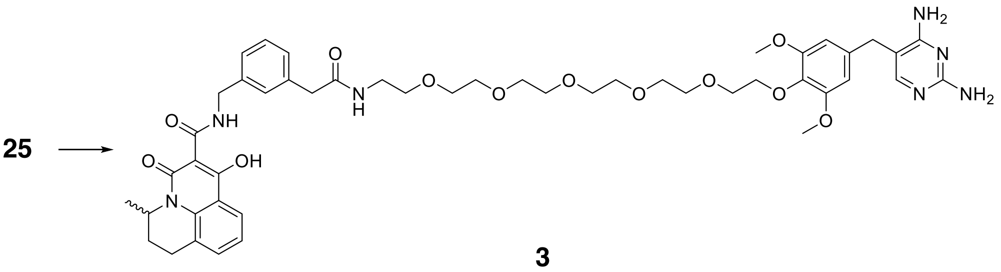

3

***N*-(3-(20-(4-((2,4-diaminopyrimidin-5-yl)methyl)-2,6-dimethoxyphenoxy)-2-oxo-6,9,12,15,18-pentaoxa-3-azaicosyl)benzyl)-7-hydroxy-3-methyl-5-oxo-2,3-dihydro-1*H*,5*H*-pyrido[3,2,1-ij]quinoline-6-carboxamide (3)**

Compound **25** (31mg, 0.08mmol, 1.0eq.) and H_2N-PEG_6-TMP (45mg, 0.08mmol, 1.1eq.) were weighed into a 4mL vial. DMF (0.75mL) and NEt_3 (43µL, 0.31mmol, 4.1eq.) were added and the resulting mixture was agitated to yield a solution. Using a micropipette, T3P-solution (≥50%w/w in EtOAc, 50µL, 0.17mmol, 2.2eq.) was added and the resulting solution was stirred overnight. A drop of MeOH was added to quench any T3P-residues, the solvent was removed by repeated coevaporation with toluene to yield a yellow gum, and the latter was dispersed over SiO_2 (approx. 0.7g). The SiO_2-plug was washed with MeOH in DCM (0→3%) and the product was eluted by repeated washed with MeOH/DCM 1:1. The residue obtained after evaporation of the solvent was purified by preparative HPLC (30→50% MeCN in 50 mM aq. NH_4HCO_3 over 10 minutes, XBridge C18 column) to yield **3** in 57% yield (40mg) as a yellow gum. Analysis by HPLC revealed, that the product was 97.3% pure. MS/ESI negative ionization: m/z=926 $[M-H]^-$.

4.4 Chromatograms and mass-spectra of the final product

Detection using DAD at 305±90nm (top panel), 254±5nm (second panel from top) and 214±5nM (bottom panel). Third panel from top: TIC (see mass spectrum on next page).

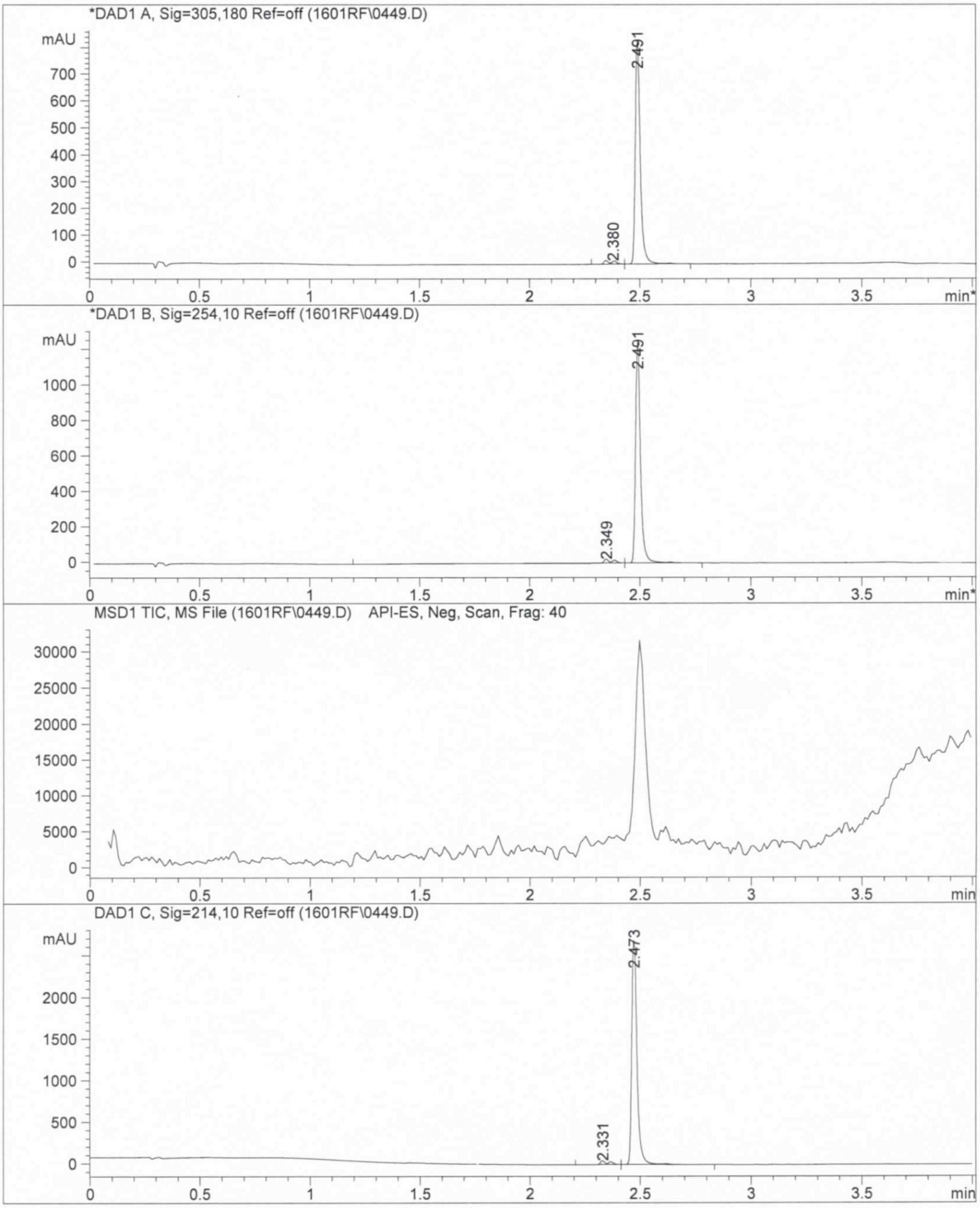

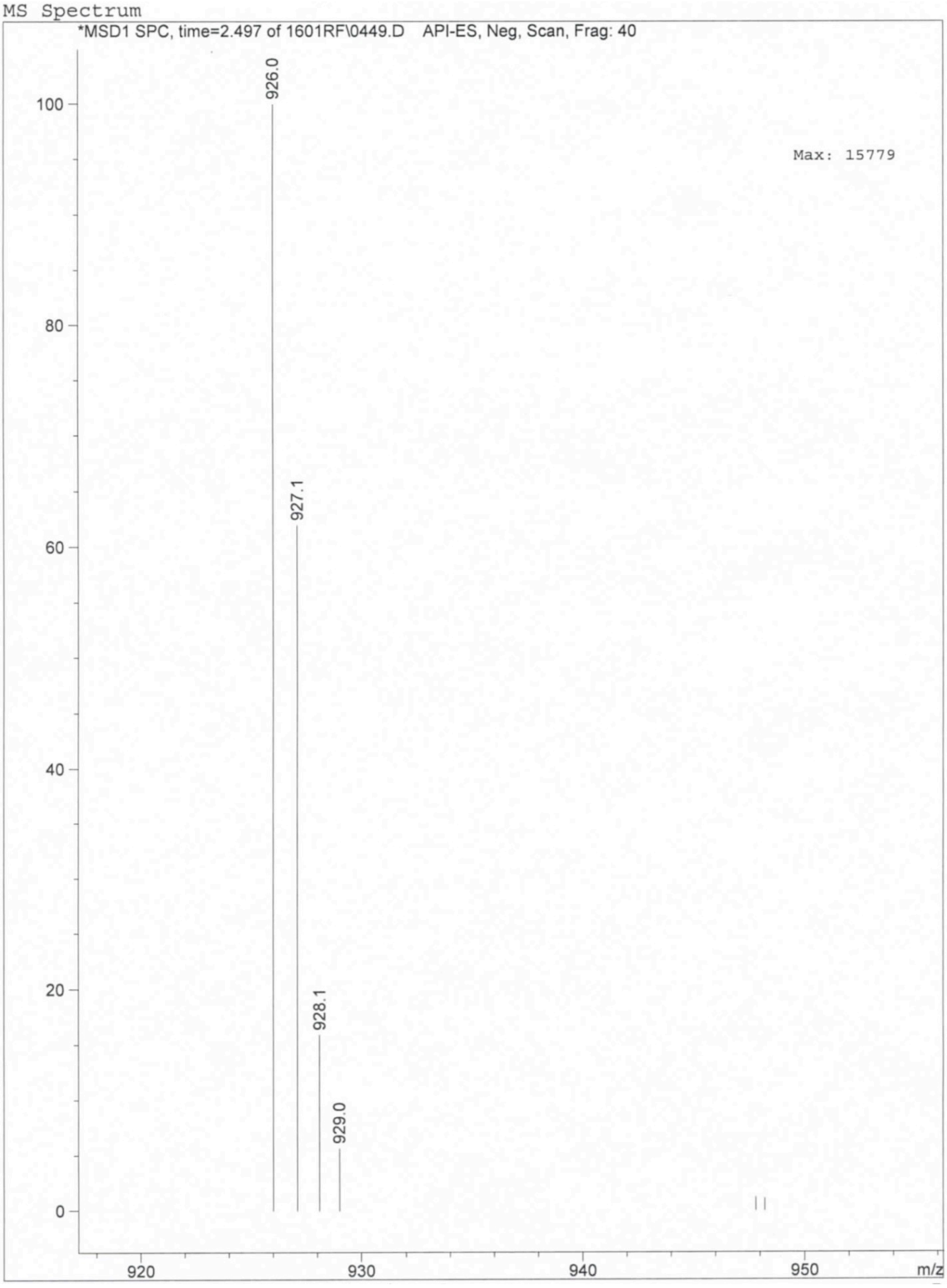

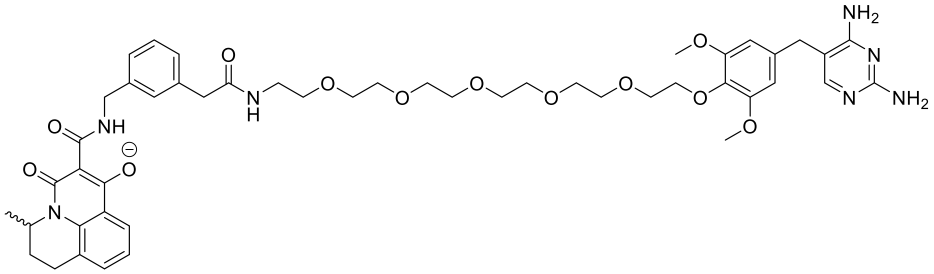

m/z (predicted): 926 (100.0%), 927 (51.9%), 928 (13.2%), 927 (2.6%), 929 (1.3%)

OnTarget Chemistry

Certificate of analysis

Date:
2015-07-17

Batch: OLA3RWH6, -8
Amount: 255.0 mg

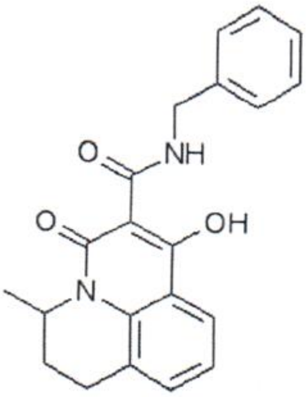

Compound: OLA3-f1

Chemical formula: $C_{21}H_{20}N_2O_3$

Molecular weight: 348.41 g/mol

Reference: *Chem. Heterocycl. Compd.*, **2006**, *42*, 1208.

Test/Method	Specification	Results
Visual appearance	-	White crystals
HPLC purity acidic<br>ACE C8, CF_3COOH (pH2), 3 min gradient, UV detection at 254 nm	>95%	>99.9%
HPLC purity basic<br>XBridge C18, NH_4HCO_3 (pH10), 3 min gradient, UV detection at 254 nm		>99.9%
MS / ESI, positive ionization	Confirms sum formula	$[M+H]^+$ = 349, 350
^1H NMR, 400 MHz, $CDCl_3$	Confirms structure	Confirms structure

**Responsible for synthesis:**	.... Rafael Hartmann, M.Sc.
**Analytical Review:**	.... Wei Berts, Ph.D.

OnTarget Chemistry AB
Virdings allé 32b
75450 Uppsala
Telefon: +46(0)76-614 8739

Org.nr: 556761-5439

E-post:
info@ontargetchemistry.com
Webb:
www.ontargetchemistry.com

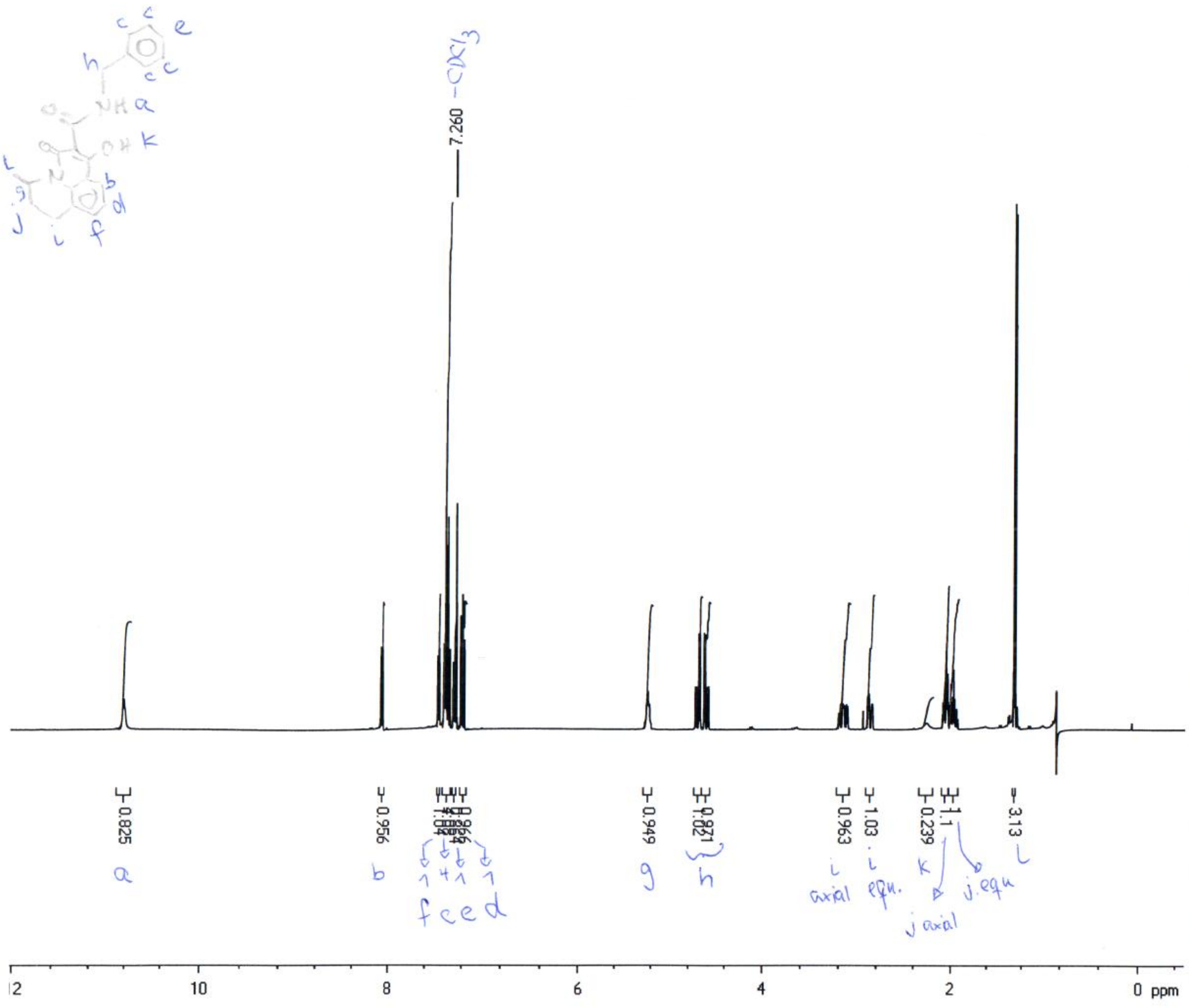

All files:
OLA3RWH6_PROTON_W01.fid

Active data:
SAMPLE
date: Jul
solvent: cdcl3
file: /home/walkup/vnmrsys/Automation/auto_20150716_01/enterQ.macdir/loc6_003/current
ACQUISITION
sw: 6397.95265515
at: 2.5608192
np: 32768
fb: 3600
bs: 4
ss: 2
d1: 4
d2: 0
nt: 32
ct: 32
TRANSMITTER
tn: H1
sfrq: 399.857922554
tof: 450.07115298
tpwr: 60
pw: 5.45
p1: 0
DECOUPLER
dn: C13
dof: 0
dm: nnn
decwave: g
dpwr: 49
dmf: 18500
PRESATURATION
satmode: n
wet: n
SPECIAL
temp: 25
gain: 30
spin: 20
hst: 0.00800000037998
pw90: 10.9
alfa: 10
FLAGS
il: n
in: n
dp: y
[illegible]

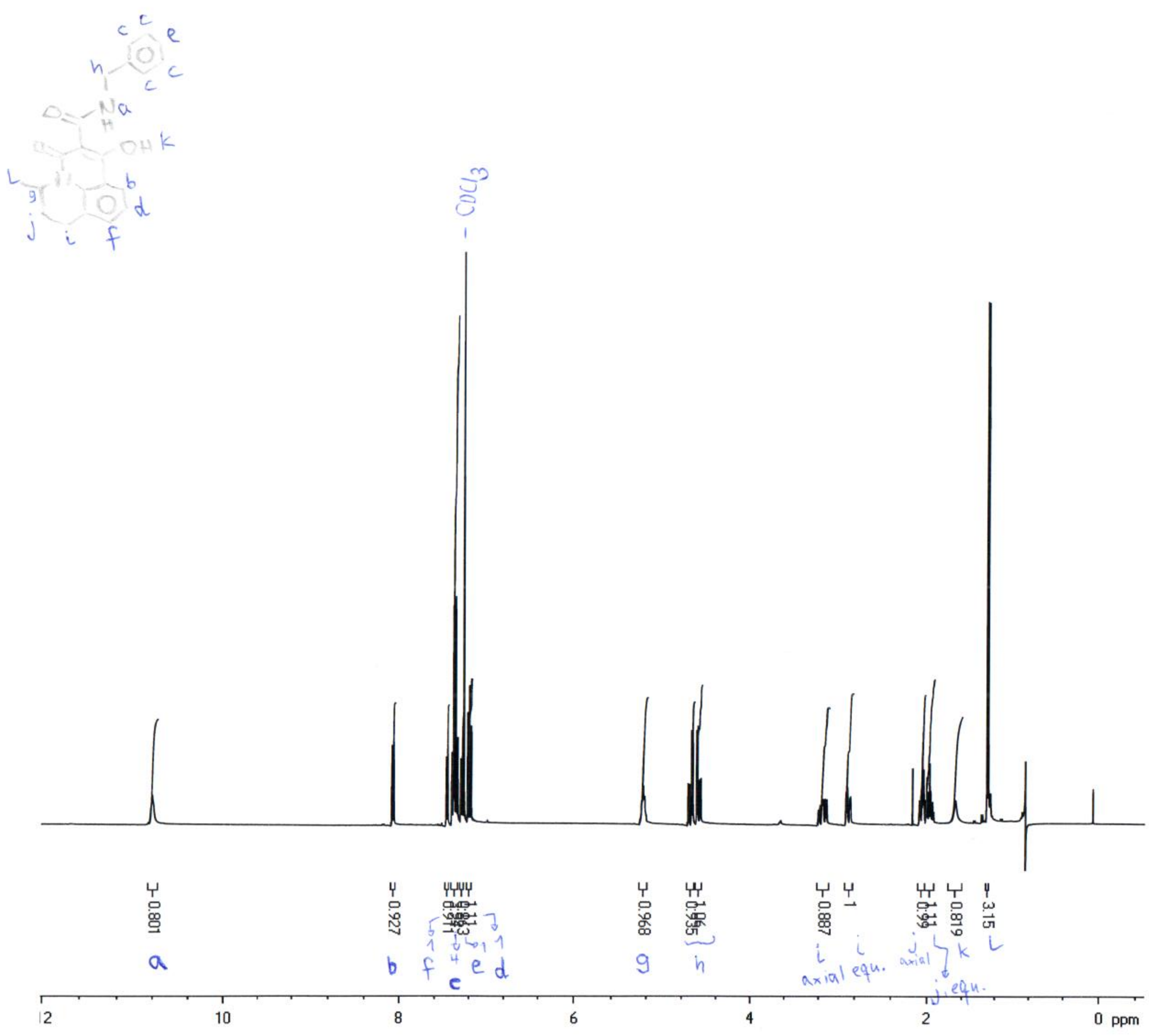

All files:
OLA3RWH8

Active data:
File: Z:/media/Server/Uppdrag/Olof A/OLA3 NMRs/OLA3RWH8
Pulse program: s2pul
Data type: spectrum (real)
Number of points: 16384
Spectrum width: 6397.95 Hz
Applied processing:
ft + aph + ph 148.081;108

```
=====================================================================
Injection Date   : 2015-07-17 08:23:33          Seq. Line :   2
Sample Name      : OLA3-f1-purity                Location : Vial 70
Acq. Operator    : Effie_rf                           Inj :   1
Acq. Instrument  : Instrument 1                Inj Volume : Inj prog
Sequence File    : C:\HPCHEM\1\SEQUENCE\USETHIS.S
Method           : C:\HPCHEM\1\METHODS\STANDARD\SX1097X3.M
Last changed     : 2015-05-14 20:36:45  by Effie_rf
STANDARD METHOD FOR REGISTRATION INTO CHEMSPEC (2'nd)
10-97% acetonitrile in 3 min, XTerra C18, 3.5u, 50x3.0, 1 ml/min, 215-395, 254 nm, A: 10mM
NH4HCO3(pHca10), B: MeCN
```

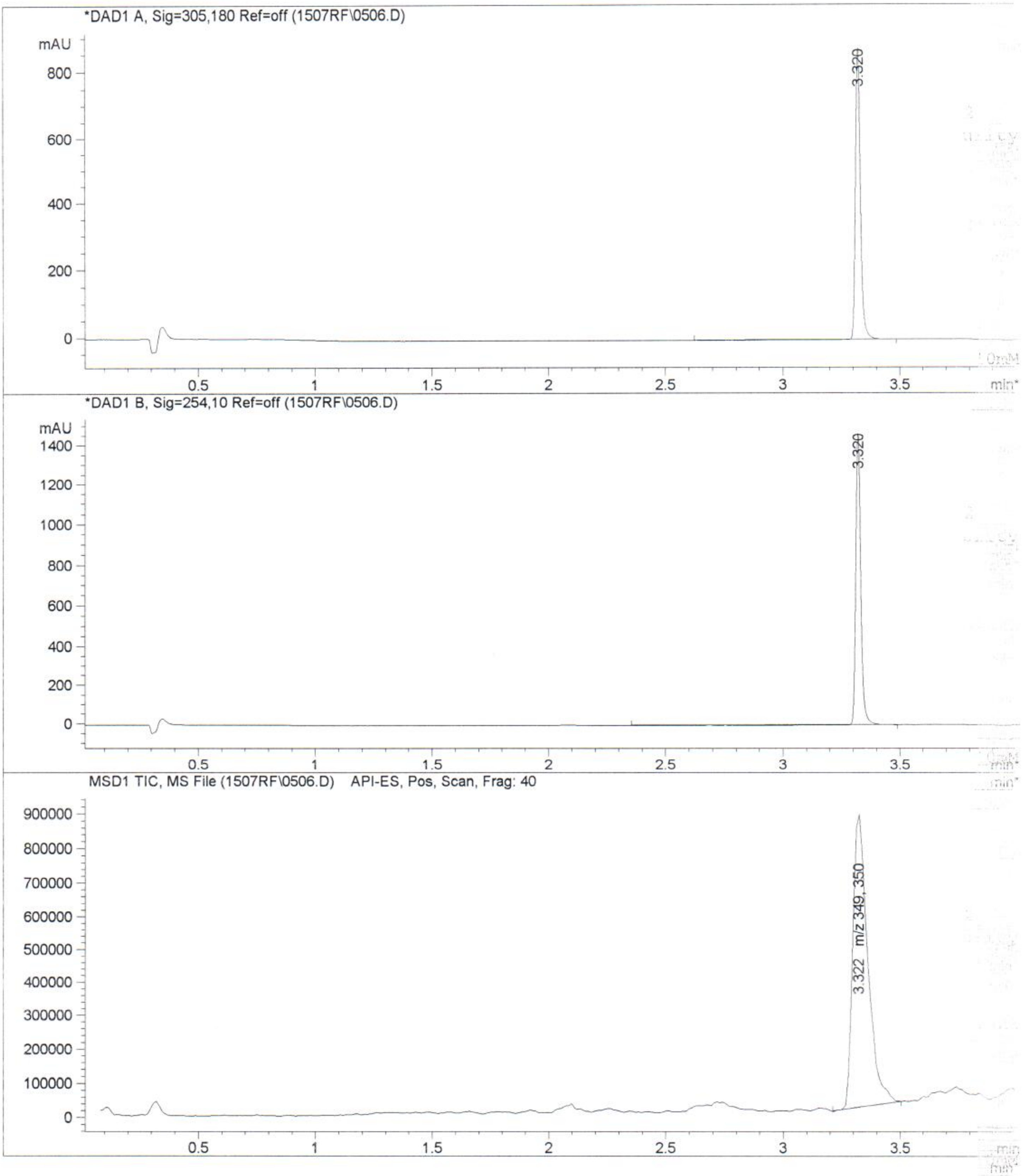

===

Area Percent Report

===

Sorted By : Signal
Multiplier : 1.0000
Dilution : 1.0000
Use Multiplier & Dilution Factor with ISTDs

Signal 1: DAD1 A, Sig=305,180 Ref=off
Signal has been modified after loading from rawdata file!

Peak #	RetTime [min]	Type	Width [min]	Area [mAU*s]	Height [mAU]	Area %
1	3.320	PB	0.0555	1266.83887	758.65112	100.0000
Totals :				1266.83887	758.65112	

Signal 2: DAD1 B, Sig=254,10 Ref=off
Signal has been modified after loading from rawdata file!

Peak #	RetTime [min]	Type	Width [min]	Area [mAU*s]	Height [mAU]	Area %
1	3.320	BBA	0.0638	2168.07495	1280.53235	100.0000
Totals :				2168.07495	1280.53235	

Signal 3: MSD1 TIC, MS File

Peak #	RetTime [min]	Type	Width [min]	Area	Height	Area %
1	3.322	BB	0.0662	3.88909e6	8.75169e5	100.0000
Totals :				3.88909e6	8.75169e5	

===

*** End of Report ***

Data File C:\HPCHEM\1\DATA\1507RF\0505.D Purity basic blank Sample Name: OLA3-f1-blank

===

```
Injection Date   : 2015-07-17 08:16:56          Seq. Line :   1
Sample Name      : OLA3-f1-blank                 Location : Vial 69
Acq. Operator    : Effie_rf                           Inj :   1
Acq. Instrument  : Instrument 1               Inj Volume : Inj prog
Sequence File    : C:\HPCHEM\1\SEQUENCE\USETHIS.S
Method           : C:\HPCHEM\1\METHODS\STANDARD\SX1097X3.M
Last changed     : 2015-05-14 20:36:45  by Effie_rf
STANDARD METHOD FOR REGISTRATION INTO CHEMSPEC (2'nd)
10-97% acetonitrile in 3 min, XTerra C18, 3.5u, 50x3.0, 1 ml/min, 215-395, 254 nm, A: 10mM
NH4HCO3(pHca10), B: MeCN
```

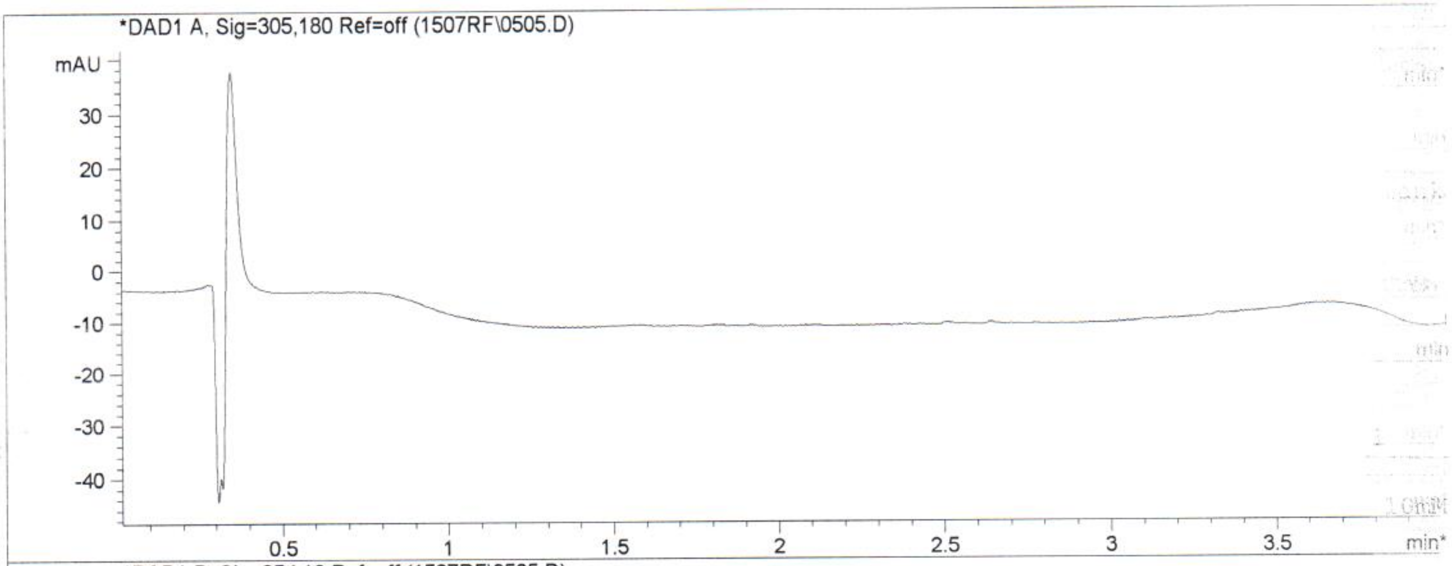

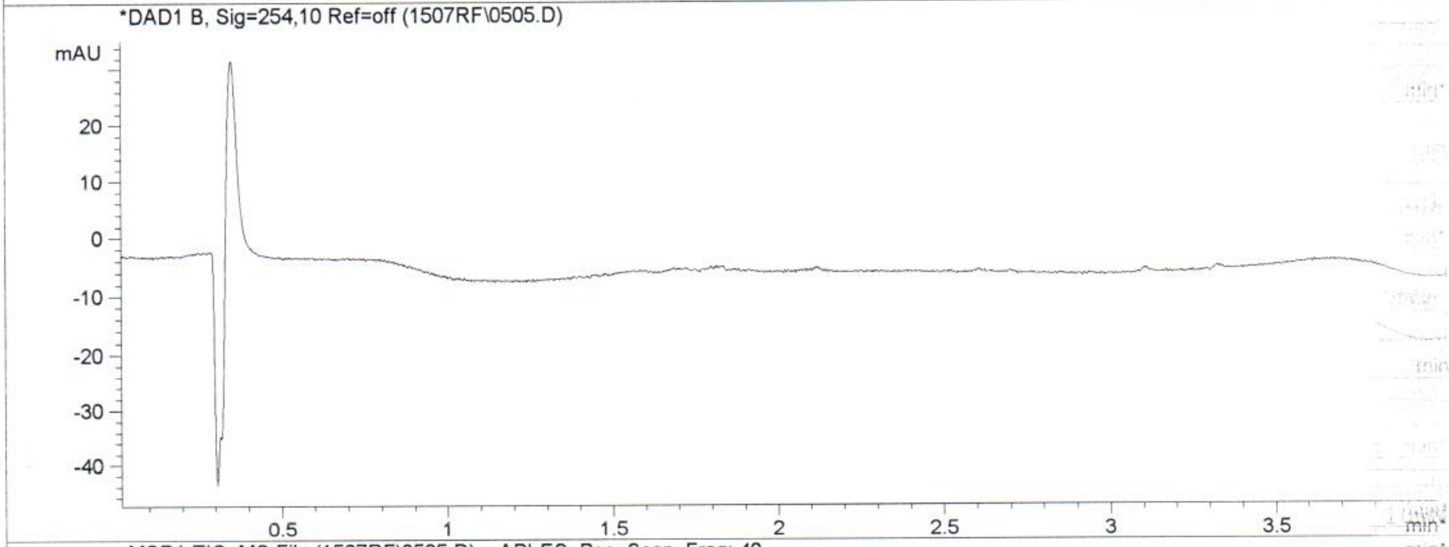

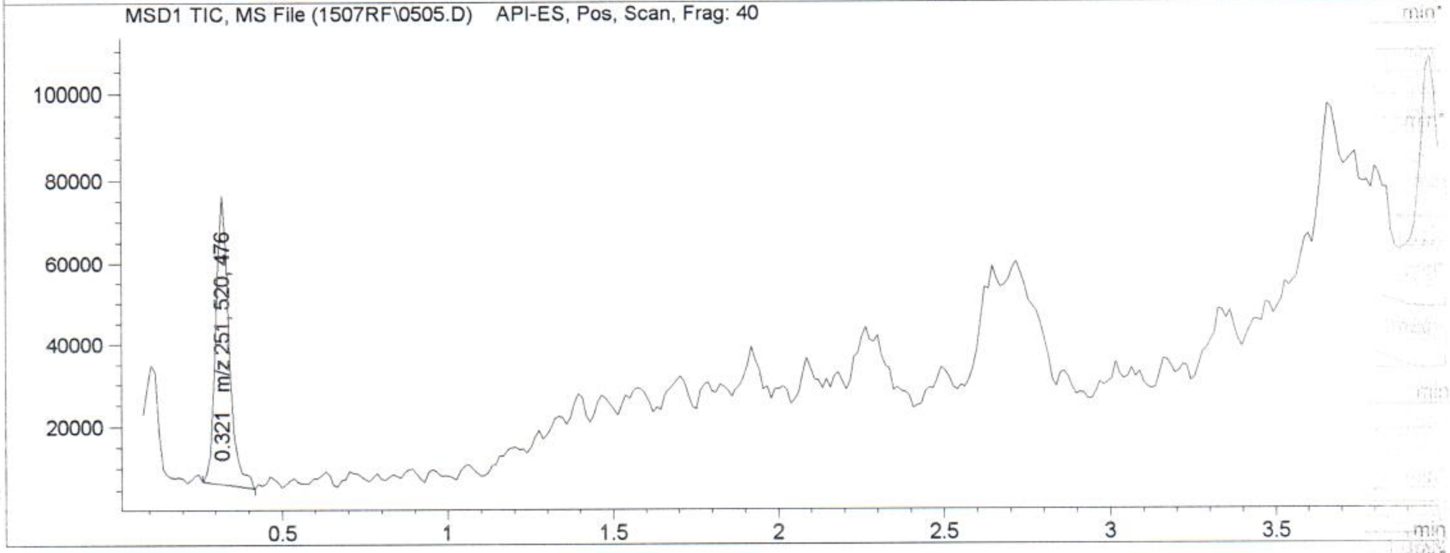

===

Area Percent Report

===

Sorted By : Signal
Multiplier : 1.0000
Dilution : 1.0000
Use Multiplier & Dilution Factor with ISTDs

Signal 1: DAD1 A, Sig=305,180 Ref=off
Signal has been modified after loading from rawdata file!

Signal 2: DAD1 B, Sig=254,10 Ref=off
Signal has been modified after loading from rawdata file!

Signal 3: MSD1 TIC, MS File

Peak #	RetTime [min]	Type	Width [min]	Area	Height	Area %
1	0.321	BB	0.0427	1.92084e5	7.00760e4	100.0000

Totals : 1.92084e5 7.00760e4

===

*** End of Report ***

===

Injection Date : 2015-07-17 08:46:46 Seq. Line : 2
Sample Name : OLA3-f1-purity Location : Vial 70
Acq. Operator : Effie_rf Inj : 1
Acq. Instrument : Instrument 1 Inj Volume : Inj prog
Sequence File : C:\HPCHEM\1\SEQUENCE\USETHIS.S
Method : C:\HPCHEM\1\METHODS\STANDARD\ST1097A3.M
Last changed : 2015-06-01 09:19:34 by Effie_rf
STANDARD METHOD FOR REGISTRATION INTO CHEMSPEC
ACE 3 C8 50x3.0 mm, 10-97% acetonitrile in 3 min, 1 ml/min
215-395 & 254 nm

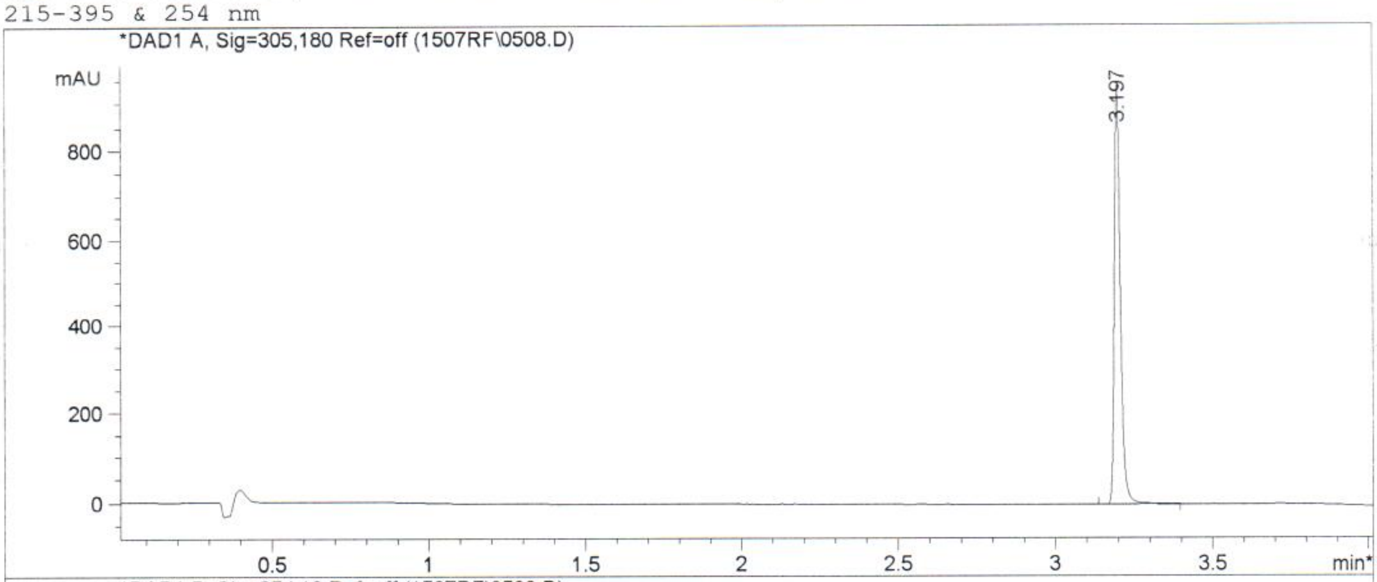

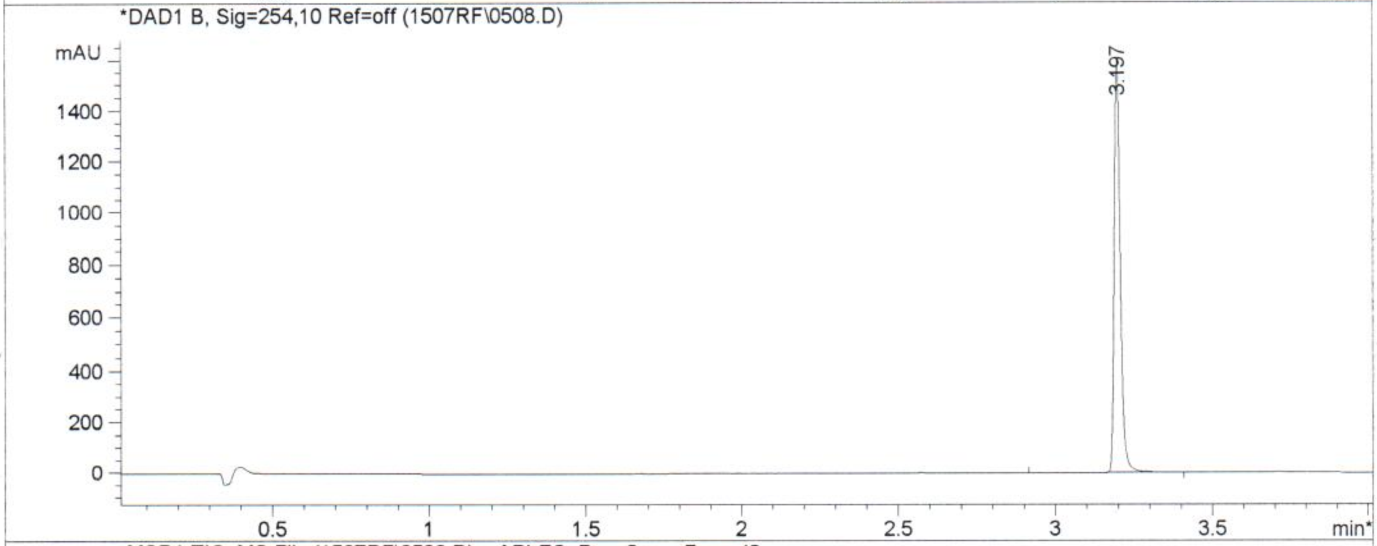

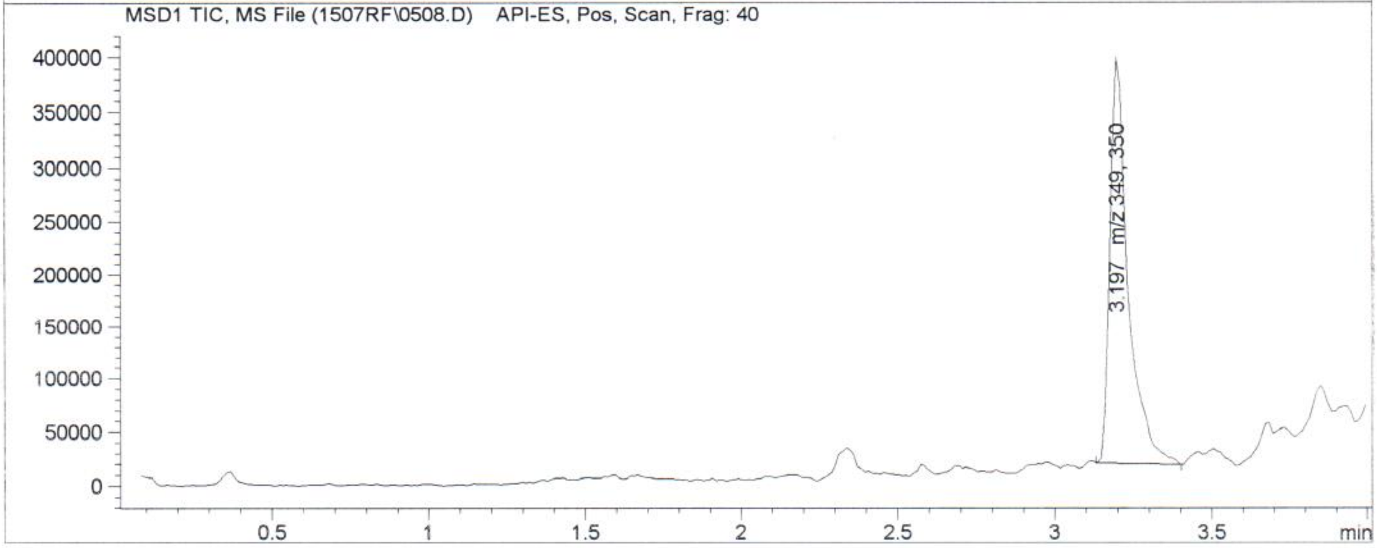

Area Percent Report

Sorted By : Signal
Multiplier : 1.0000
Dilution : 1.0000
Use Multiplier & Dilution Factor with ISTDs

Signal 1: DAD1 A, Sig=305,180 Ref=off
Signal has been modified after loading from rawdata file!

Peak #	RetTime [min]	Type	Width [min]	Area [mAU*s]	Height [mAU]	Area %
1	3.197	PBA	0.0293	1275.48010	858.55267	100.0000
Totals :				1275.48010	858.55267	

Signal 2: DAD1 B, Sig=254,10 Ref=off
Signal has been modified after loading from rawdata file!

Peak #	RetTime [min]	Type	Width [min]	Area [mAU*s]	Height [mAU]	Area %
1	3.197	BBA	0.0295	2182.70093	1455.14075	100.0000
Totals :				2182.70093	1455.14075	

Signal 3: MSD1 TIC, MS File

Peak #	RetTime [min]	Type	Width [min]	Area	Height	Area %
1	3.197	BB	0.0573	1.52962e6	3.82860e5	100.0000
Totals :				1.52962e6	3.82860e5	

*** End of Report ***

Purity ~~basic~~ acidic
blank

===

Injection Date : 2015-07-17 08:39:54 Seq. Line : 1
Sample Name : OLA3-f1-blank Location : Vial 69
Acq. Operator : Effie_rf Inj : 1
Acq. Instrument : Instrument 1 Inj Volume : Inj prog
Sequence File : C:\HPCHEM\1\SEQUENCE\USETHIS.S
Method : C:\HPCHEM\1\METHODS\STANDARD\ST1097A3.M
Last changed : 2015-06-01 09:19:34 by Effie_rf
STANDARD METHOD FOR REGISTRATION INTO CHEMSPEC
ACE 3 C8 50x3.0 mm, 10-97% acetonitrile in 3 min, 1 ml/min
215-395 & 254 nm

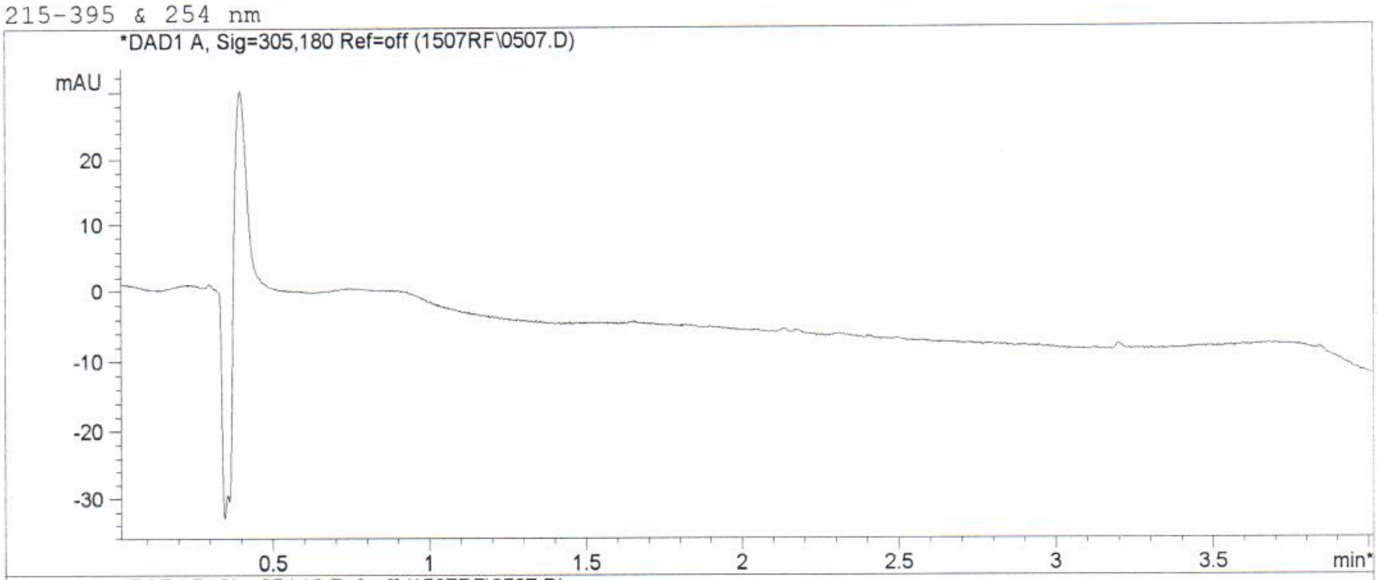

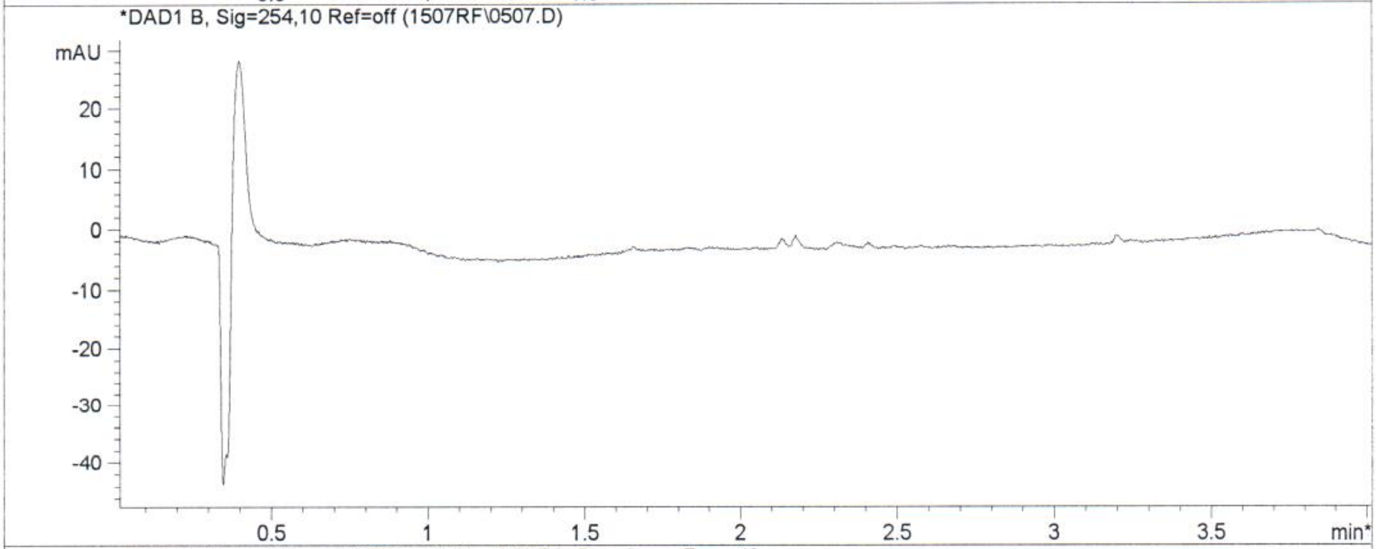

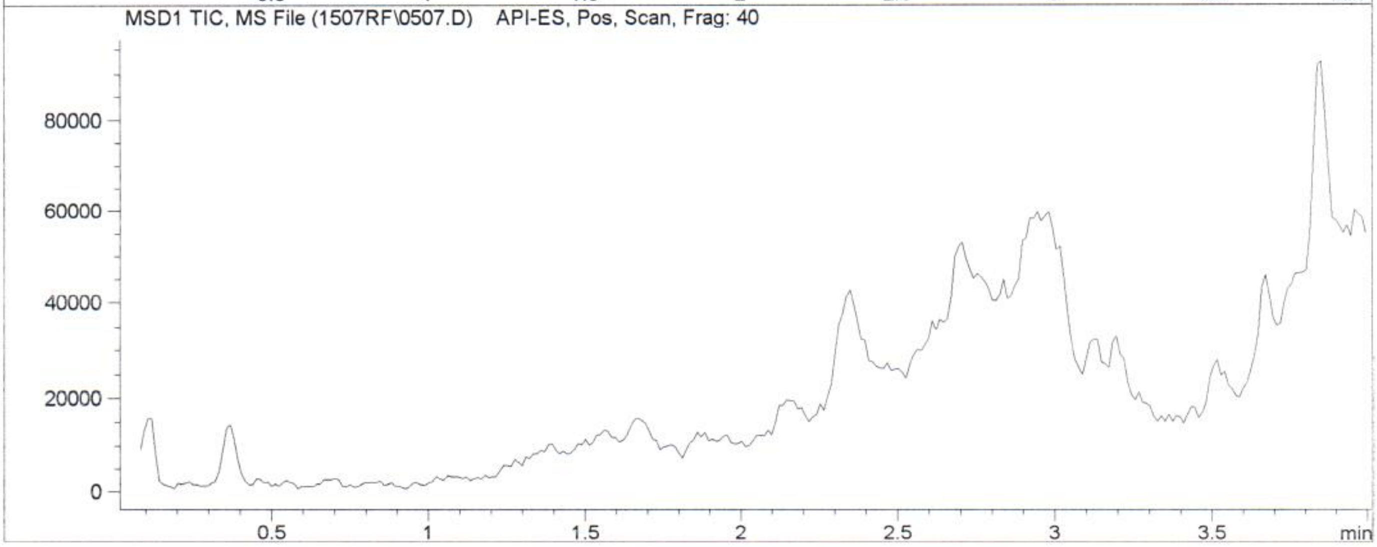

===

Area Percent Report

===

Sorted By : Signal
Multiplier : 1.0000
Dilution : 1.0000
Use Multiplier & Dilution Factor with ISTDs

No peaks found

===

*** End of Report ***

Supplementary Note 2

HYBRIGENICS SERVICES

Results Summary

YChemH SCREEN
TMP-PEG5 - OCT140805 / HBX129659
# vs Human Islets Langerhans_RP1

Mon, Nov 21, 2016 - 04:31 PM

Screen Parameters

Nature	cDNA
Reference Bait	TMP-PEG5 - OCT140805 / HBX129659 ; hgx4366v1
Prey Library	Human Islets Langerhans_RP1
Vector(s)	pB409 (N-LexA-eDHFR-C)
Processed Clones	76 (pB409_A)
Analyzed Interactions	135 millions (pB409_A)
3AT Concentration	0.0 mM (pB409_A)

Global PBS®

Global PBS (for Interactions represented in the Screen)		Nb	%
A	Very high confidence in the interaction	1	3.8%
B	High confidence in the interaction	5	19.2%
C	Good confidence in the interaction	1	3.8%
D	Moderate confidence in the interaction<br>This category is the most difficult to interpret because it mixes two classes of interactions :<br>- False-positive interactions<br>- Interactions hardly detectable by the Y2H technique (low representation of the mRNA in the library, prey folding, prey toxicity in yeast)	15	57.7%
E	Interactions involving highly connected prey domains, warning of non-specific interaction. The threshold for high connectivity is 6. They can be classified in different categories:<br>- Prey proteins that are known to be highly connected due to their biological function<br>- Proteins with a prey interacting domain that contains a known protein interaction motif or a biochemically promiscuous motif	4	15.4%
F	Experimentally proven technical artifacts	0	0.0%
Non Appliable			
N/A	The PBS is a score that is automatically computed through algorithms and cannot be attributed for the following reasons :<br>- All the fragments of the same reference CDS are antisense<br>- The 5p sequence is missing<br>- All the fragments of the same reference CDS are either all OOF1 or all OOF2<br>- All the fragments of the same reference CDS lie in the 5' or 3' UTR		

HYBRIGENICS CONFIDENTIAL - 1079262 - Karolinska Institute

Prey Fragment Analysis

Symbols	Means
	The fragment contains the full length CDS
	Fragment is fully in 5' UTR
	Fragment is fully in 3' UTR
✖	Fragment contains at least one In Frame STOP codon
[NR]	Fragment was found to be non relevant (poor quality, high N density)
IF<br>OOF1<br>OOF2	With regard to the theoretical frame of each corresponding CDS (GeneBank),fragments are cloned in frame (IF) if they are in the same frame as Gal4AD.In general, polypeptides synthesized from OOF fragments are not considered of biological interest, unless found together with another frame. However, some of the proteins expressed from an OOF fragment can be translated in the correct frame,due to the existence of natural frame-shift events during translation in yeast
??	Unidentified frame when :<br>- The clone sequence is antisense<br>- The 5p sequence is missing
N	Antisense
Start...Stop	Position of the 5p and 3p prey fragment ends, relative to the position of the ATG start codon (A=0)

Clone Name	Type Seq	Gene Name (Best Match)	Start..Stop (nt)	Frame	Sense	%Id 5p	%Id 3p	PBS
pB409_A-163	5p/3p	Homo sapiens - ALDH3A2	-161..1362	OOF2		94.6	98.1	B
pB409_A-3	5p/3p	Homo sapiens - ALDH3A2	-160..1362	IF		97.3	98.9	B
pB409_A-41	5p/3p	Homo sapiens - ALDH3A2	-160..1362	IF		99.1	98.4	B
pB409_A-187	5p/3p	Homo sapiens - ALDH3A2	-160..1362	IF		97.6	94.5	B
pB409_A-6	5p/3p	Homo sapiens - ALDH3A2	-160..1362	IF		100.0	95.2	B
pB409_A-200	5p/3p	Homo sapiens - ALDH3A2	-160..1362	IF		92.9	94.7	B
pB409_A-80	5p/3p	Homo sapiens - ALDH3A2	-160..1362	IF		89.4	97.4	B
pB409_A-182	5p/3p	Homo sapiens - ALDH3A2	-160..1362	IF		97.1	88.5	B
pB409_A-2	5p/3p	Homo sapiens - ALDH3A2	-160..1362	IF		72.8	93.5	B
pB409_A-201	5p/3p	Homo sapiens - ALDH3A2	-29..1384	IF		99.9	97.9	B
pB409_A-90	5p/3p	Homo sapiens - CBARA1	114..1404	IF		97.5	97.6	D
pB409_A-35	5p/3p	Homo sapiens - CBARA1	114..1404	IF		99.2	97.8	D
pB409_A-106	5p/3p	Homo sapiens - DNM1L var1	-70..280	IF		100.0	100.0	D
pB409_A-98	5p	Homo sapiens - DNMT3A	1887	IF		97.4		D
pB409_A-49	5p/3p	Homo sapiens - DOT1L	3342..2915	??	N	98.3	98.3	N/A
pB409_A-149	5p/3p	Homo sapiens - DZIP1	1859..2471	OOF2		99.5	99.5	N/A
pB409_A-190	5p/3p	Homo sapiens - EPS8L2	135..1404	IF		99.7	98.9	D
pB409_A-19	5p/3p	Homo sapiens - FIZ1	213..1654 ✖	IF		93.9	95.3	D
pB409_A-136	5p/3p	Homo sapiens - FN1	5712..6196	IF		100.0	100.0	D
pB409_A-162	5p/3p	Homo sapiens - FN1	5712..6196	IF		99.8	100.0	D
pB409_A-24	5p/3p	Homo sapiens - FN1	5712..6196	IF		100.0	100.0	D

HYBRIGENICS CONFIDENTIAL - 1079262 - Karolinska Institute

Clone Name	Type Seq	Gene Name (Best Match)	Start..Stop (nt)	Frame	Sense	%Id 5p	%Id 3p	PBS
pB409_A-160	5p/3p	Homo sapiens - FN1	5712..6196	IF		99.8	100.0	D
pB409_A-38	5p	Homo sapiens - HEY-L	99..477	IF		100.0		E
pB409_A-184	5p	Homo sapiens - HLA-A	481..382	??	N	100.0		N/A
pB409_A-129	5p/3p	Homo sapiens - HMG20A	21..594	IF		99.7	100.0	D
pB409_A-66	5p/3p	Homo sapiens - HNRNPUL1	-97..1582	IF		99.2	94.8	B
pB409_A-154	5p/3p	Homo sapiens - HNRNPUL1	1518..2105	IF		94.2	99.8	B
pB409_A-199	5p/3p	Homo sapiens - HNRNPUL1	1530..2228	IF		93.0	97.7	B
pB409_A-144	5p/3p	Homo sapiens - HNRPU	73..252	OOF1		99.4	100.0	N/A
pB409_A-110	5p/3p	Homo sapiens - ICMT	3969..4376	IF		99.5	100.0	N/A
pB409_A-88	5p/3p	Homo sapiens - ISOC2	-85..772	IF		96.5	98.2	C
pB409_A-73	5p/3p	Homo sapiens - ISOC2	-82..679	IF		96.9	98.1	C
pB409_A-26	5p/3p	Homo sapiens - KDM2A	42..569	IF		100.0	99.8	D
pB409_A-101	5p	Homo sapiens - MKNK2	72	IF		92.9		A
pB409_A-170	5p/3p	Homo sapiens - MKNK2	105..1303	IF		97.0	100.0	A
pB409_A-76	5p/3p	Homo sapiens - MKNK2	117..1299	IF		96.3	97.4	A
pB409_A-171	5p/3p	Homo sapiens - MKNK2	117..1299	IF		96.5	96.2	A
pB409_A-70	5p/3p	Homo sapiens - MKNK2	204..1301	IF		98.3	97.4	A
pB409_A-11	5p/3p	Homo sapiens - MKNK2	204..1301	IF		98.9	97.6	A
pB409_A-147	5p/3p	Homo sapiens - MTRNR2L2	-832..-396	IF		95.5	96.1	N/A
pB409_A-13	5p/3p	Homo sapiens - PDE4D	1152..2116	IF		96.6	98.2	D
pB409_A-94	5p/3p	Homo sapiens - PDE4D	1152..2116	IF		97.4	98.7	D
pB409_A-68	5p/3p	Homo sapiens - PDE4D	1152..2116	IF		96.2	98.0	D
pB409_A-16	5p/3p	Homo sapiens - PECI	-73..922	IF		96.6	92.6	D
pB409_A-14	5p/3p	Homo sapiens - PECI	-73..922	IF		97.2	96.5	D
pB409_A-95	5p/3p	Human - PECR	-55..926	IF		99.7	98.1	D
pB409_A-137	5p/3p	Human - PECR	-55..926	IF		98.0	96.4	D
pB409_A-208	5p/3p	Homo sapiens - PRDM15	3135..4083	IF		98.9	98.1	E
pB409_A-104	5p	Homo sapiens - RBBP5	260	OOF2		96.6		N/A
pB409_A-153	3p	Homo sapiens - SEC31A	3584..3945	OOF2			99.4	N/A
pB409_A-206	5p/3p	Homo sapiens - SIRT6	6..821	IF		98.0	98.2	B
pB409_A-194	5p/3p	Homo sapiens - SIRT6	9..1034	IF		95.8	97.6	B
pB409_A-169	5p/3p	Homo sapiens - SIRT6	42..920	IF		94.7	96.9	B
pB409_A-183	5p/3p	Homo sapiens - SIRT6	42..920	IF		97.7	98.1	B
pB409_A-46	3p	Homo sapiens - SMURF1	..1361	??			80.3	N/A
pB409_A-22	5p/3p	Homo sapiens - SMURF1	-310..1439	IF		98.2	93.7	E
pB409_A-134	5p/3p	Homo sapiens - SMURF1	-310..1439	IF		85.3	98.0	E
pB409_A-138	5p/3p	Homo sapiens - SMURF1	-310..1439	IF		99.9	88.9	E

Clone Name	Type Seq	Gene Name (Best Match)	Start..Stop (nt)	Frame	Sense	%Id 5p	%Id 3p	PBS
pB409_A-28	5p	Homo sapiens - SMURF1	-26	OOF2		92.7		E
pB409_A-127	5p/3p	Homo sapiens - SNRNP200	5133..5259	IF		71.8	97.4	D
pB409_A-10	5p/3p	Homo sapiens - TMED4	298..689 ✗	OOF1		97.4	99.5	N/A
pB409_A-108	5p/3p	Homo sapiens - ZFX	1275..1868	IF		99.5	99.8	D
pB409_A-93	3p	Homo sapiens - ZNF219	..1632	??			94.6	E
pB409_A-65	5p/3p	Homo sapiens - ZNF219	675..1626	IF		99.6	95.9	E
pB409_A-12	3p	Homo sapiens - ZNF236	..3387	??			97.8	B
pB409_A-161	5p/3p	Homo sapiens - ZNF236	1890..3387	IF		97.5	98.3	B
pB409_A-135	5p/3p	Homo sapiens - ZNF236	1890..3387	IF		97.7	96.1	B
pB409_A-29	5p/3p	Homo sapiens - ZNF236	2694..3810	IF		97.2	97.4	B
pB409_A-139	5p/3p	Homo sapiens - ZNF236	2694..3810	IF		95.8	98.0	B
pB409_A-84	5p/3p	Homo sapiens - ZNF236	2694..3810	IF		99.1	99.2	B
pB409_A-213	5p/3p	Homo sapiens - ZNF267	888..1336	IF		100.0	99.6	D
pB409_A-99	5p	Homo sapiens - ZNF584	147	IF		95.6		B
pB409_A-79	5p/3p	Homo sapiens - ZNF584	147..1686 ✗	IF		85.0	98.3	B
pB409_A-198	5p/3p	Homo sapiens - ZNF584	513..1233	IF		95.8	97.1	B
pB409_A-195	5p/3p	Homo sapiens - ZNF584	513..1233	IF		95.7	98.1	B
pB409_A-85	5p/3p	Homo sapiens - GenMatch	-1..87 ✗	IF		100.0	100.0	D

Results Summary

YChemH SCREEN
TMP-PEG5 - OCT140805 / HBX129659
# vs Zebrafish Embryo_RP1 (18-20 hpf)

Mon, Nov 21, 2016 - 10:47 AM

Screen Parameters

Nature	cDNA
Reference Bait	TMP-PEG5 - OCT140805 / HBX129659 ; hgx4366v1
Prey Library	Zebrafish Embryo_RP1 (18-20 hpf)
Vector(s)	pB409 (N-LexA-eDHFR-C)
Processed Clones	54 (pB409_A)
Analyzed Interactions	122 millions (pB409_A)
3AT Concentration	0.0 mM (pB409_A)

Global PBS®

Global PBS (for Interactions represented in the Screen)		Nb	%
A	Very high confidence in the interaction	2	9.5%
B	High confidence in the interaction	1	4.8%
C	Good confidence in the interaction	1	4.8%
D	Moderate confidence in the interaction<br>This category is the most difficult to interpret because it mixes two classes of interactions :<br>- False-positive interactions<br>- Interactions hardly detectable by the Y2H technique (low representation of the mRNA in the library, prey folding, prey toxicity in yeast)	17	81.0%
E	Interactions involving highly connected prey domains, warning of non-specific interaction. The threshold for high connectivity is 6. They can be classified in different categories:<br>- Prey proteins that are known to be highly connected due to their biological function<br>- Proteins with a prey interacting domain that contains a known protein interaction motif or a biochemically promiscuous motif	0	0.0%
F	Experimentally proven technical artifacts	0	0.0%
Non Appliable			
N/A	The PBS is a score that is automatically computed through algorithms and cannot be attributed for the following reasons :<br>- All the fragments of the same reference CDS are antisense<br>- The 5p sequence is missing<br>- All the fragments of the same reference CDS are either all OOF1 or all OOF2<br>- All the fragments of the same reference CDS lie in the 5' or 3' UTR		

HYBRIGENICS CONFIDENTIAL - 1067989 - Karolinska Institute

Prey Fragment Analysis

Symbols	Means
	The fragment contains the full length CDS
	Fragment is fully in 5' UTR
	Fragment is fully in 3' UTR
✖	Fragment contains at least one In Frame STOP codon
[NR]	Fragment was found to be non relevant (poor quality, high N density)
IF<br>OOF1<br>OOF2	With regard to the theoretical frame of each corresponding CDS (GeneBank),fragments are cloned in frame (IF) if they are in the same frame as Gal4AD.In general, polypeptides synthesized from OOF fragments are not considered of biological interest, unless found together with another frame. However, some of the proteins expressed from an OOF fragment can be translated in the correct frame,due to the existence of natural frame-shift events during translation in yeast
??	Unidentified frame when :<br>- The clone sequence is antisense<br>- The 5p sequence is missing
N	Antisense
Start...Stop	Position of the 5p and 3p prey fragment ends, relative to the position of the ATG start codon (A=0)

Clone Name	Type Seq	Gene Name (Best Match)	Start..Stop (nt)	Frame	Sense	%Id 5p	%Id 3p	PBS
pB409_A-39	5p/3p	Danio rerio - LOC100333857	30..865 ✖	IF		96.3	95.9	D
pB409_A-130	5p/3p	Danio rerio - LOC100333857	30..865 ✖	IF		97.5	96.9	D
pB409_A-52	5p/3p	Danio rerio - LOC103909511	492..985	IF		100.0	99.2	D
pB409_A-103	3p	Danio rerio - LOC108183936	..1049	??			98.3	D
pB409_A-232	5p/3p	Danio rerio - LOC108183936	591..1049	IF		98.9	98.9	D
pB409_A-335	5p/3p	Danio rerio - LOC559111	33..1260	IF		95.9	96.7	D
pB409_A-238	5p/3p	Danio rerio - LOC560099	3126..4216	IF		94.9	85.7	D
pB409_A-360	5p/3p	Danio rerio - LOC567317	-1..874	IF		95.0	91.6	D
pB409_A-209	5p/3p	Danio rerio - acin1b	2568..3337	IF		96.2	97.0	A
pB409_A-233	5p/3p	Danio rerio - acin1b	2592..3316	IF		98.5	97.4	A
pB409_A-284	5p/3p	Danio rerio - acin1b	2592..3316	IF		99.0	97.5	A
pB409_A-243	5p/3p	Danio rerio - acin1b	2655..3232	IF		99.7	98.3	A
pB409_A-208	5p/3p	Danio rerio - acin1b	2676..3288	IF		99.5	98.5	A
pB409_A-236	5p/3p	Danio rerio - acin1b	2676..3288	IF		99.5	99.0	A
pB409_A-169	5p/3p	Danio rerio - acin1b	2676..3288	IF		99.5	98.2	A
pB409_A-222	5p/3p	Danio rerio - acin1b	2682..3488	IF		96.6	97.6	A
pB409_A-289	5p/3p	Danio rerio - acin1b	2682..3488	IF		96.0	96.9	A
pB409_A-213	5p/3p	Danio rerio - acin1b	2730..3553	IF		93.7	98.4	A

Clone Name	Type Seq	Gene Name (Best Match)	Start..Stop (nt)	Frame	Sense	%Id 5p	%Id 3p	PBS
pB409_A-318	5p/3p	Danio rerio - acin1b	2730..3553	IF		97.7	92.3	A
pB409_A-171	5p/3p	Danio rerio - ctslb	525..26	??	N	99.8	99.8	N/A
pB409_A-278	5p/3p	Danio rerio - drl	426..1316 ✖	IF		95.8	96.2	A
pB409_A-80	5p/3p	Danio rerio - drl	588..1154	IF		99.6	98.1	A
pB409_A-74	5p/3p	Danio rerio - drl	588..1154	IF		99.8	99.6	A
pB409_A-353	5p/3p	Danio rerio - drl	615..1085	IF		98.5	98.3	A
pB409_A-345	5p	Danio rerio - drl	615	IF		95.0		A
pB409_A-287	5p/3p	Danio rerio - drl	615..1085	IF		98.5	98.5	A
pB409_A-152	5p	Danio rerio - drl	639	IF		88.2		A
pB409_A-122	5p/3p	Danio rerio - drl	639..1338 ✖	IF		96.6	95.3	A
pB409_A-253	5p/3p	Danio rerio - drl	639..1338 ✖	IF		97.9	95.3	A
pB409_A-151	3p	Danio rerio - exoc6b	..2288	??			100.0	D
pB409_A-20	5p/3p	Danio rerio - exoc6b	459..2288	IF		98.9	98.1	D
pB409_A-64	5p/3p	Danio rerio - exoc6b	459..2288	IF		99.2	97.1	D
pB409_A-7	5p/3p	Danio rerio - exoc6b	459..2288	IF		90.0	97.8	D
pB409_A-167	5p/3p	Danio rerio - exoc6b	459..2288	IF		99.0	98.4	D
pB409_A-239	5p/3p	Danio rerio - exoc6b	459..2288	IF		99.0	99.8	D
pB409_A-172	5p	Danio rerio - kdm2ab	1164	IF		99.7		D
pB409_A-177	5p/3p	Danio rerio - kmt2ba	4086..5053	IF		96.5	100.0	D
pB409_A-49	5p/3p	Danio rerio - osbpl3b	1215..3110 ✖	IF		99.7	99.7	D
pB409_A-13	5p/3p	Danio rerio - pax6b	312..903	IF		100.0	100.0	D
pB409_A-92	5p/3p	Danio rerio - pif1	15..434	IF		98.8	98.8	D
pB409_A-113	5p/3p	Danio rerio - pls3	138..1358	IF		93.4	98.1	C
pB409_A-42	5p/3p	Danio rerio - pls3	297..1538	IF		97.7	97.5	C
pB409_A-294	5p/3p	Danio rerio - ppp2r5cb	1691..337	??	N	98.2	97.2	N/A
pB409_A-73	5p/3p	Danio rerio - prdm15	1602..2573	IF		97.2	96.4	D
pB409_A-293	5p/3p	Danio rerio - safb	1113..2102	IF		99.1	97.5	D
pB409_A-336	5p/3p	Danio rerio - si:ch211-212k18.5	1932..2691	IF		99.7	99.2	D
pB409_A-51	5p/3p	Danio rerio - stil	75..1132	IF		97.1	97.8	D
pB409_A-124	5p/3p	Danio rerio - stil	75..1132	IF		94.1	97.6	D
pB409_A-140	5p/3p	Danio rerio - vars	2700..3268	IF		99.5	99.3	B
pB409_A-137	5p/3p	Danio rerio - vars	2700..3268	IF		99.5	99.3	B
pB409_A-295	5p/3p	Danio rerio - vars	2700..3268	IF		99.5	99.5	B
pB409_A-240	5p/3p	Danio rerio - vars	2799..3328	IF		99.4	99.4	B
pB409_A-248	5p/3p	Danio rerio - zgc:64002	12..835 ✖	IF		97.7	94.3	D

Clone Name	Type Seq	Gene Name (Best Match)	Start..Stop (nt)		Frame	Sense	%Id 5p	%Id 3p	PBS
pB409_A-286	5p/3p	Danio rerio - zgc:64002	12..835	✖	IF		99.4	98.9	D
