# Supplementary material for: MNK2 deficiency potentiates β-cell regeneration via translational regulation
Source: Nat Chem Biol. 2022 Jun 13;18(9):942–53. doi: 10.1038/s41589-022-01047-x (PMC7613404; doi:10.1038/s41589-022-01047-x)
Supplement: Supplementary file 2 — Reporting Summary [file 41589_2022_1047_MOESM2_ESM.pdf]

nature research

Corresponding author(s): Dr. Olov Andersson

Last updated by author(s): Apr 8, 2022

Reporting Summary

Nature Research wishes to improve the reproducibility of the work that we publish. This form provides structure for consistency and transparency in reporting. For further information on Nature Research policies, see our Editorial Policies and the Editorial Policy Checklist.

Statistics

For all statistical analyses, confirm that the following items are present in the figure legend, table legend, main text, or Methods section.

n/a	Confirmed	
☐	☒	The exact sample size (n) for each experimental group/condition, given as a discrete number and unit of measurement
☐	☒	A statement on whether measurements were taken from distinct samples or whether the same sample was measured repeatedly
☐	☒	The statistical test(s) used AND whether they are one- or two-sided<br>*Only common tests should be described solely by name; describe more complex techniques in the Methods section.*
☒	☐	A description of all covariates tested
☒	☐	A description of any assumptions or corrections, such as tests of normality and adjustment for multiple comparisons
☐	☒	A full description of the statistical parameters including central tendency (e.g. means) or other basic estimates (e.g. regression coefficient) AND variation (e.g. standard deviation) or associated estimates of uncertainty (e.g. confidence intervals)
☐	☒	For null hypothesis testing, the test statistic (e.g. F, t, r) with confidence intervals, effect sizes, degrees of freedom and P value noted<br>*Give P values as exact values whenever suitable.*
☒	☐	For Bayesian analysis, information on the choice of priors and Markov chain Monte Carlo settings
☒	☐	For hierarchical and complex designs, identification of the appropriate level for tests and full reporting of outcomes
☒	☐	Estimates of effect sizes (e.g. Cohen's d, Pearson's r), indicating how they were calculated

Our web collection on statistics for biologists contains articles on many of the points above.

Software and code

Policy information about availability of computer code

Data collection

LAS X v3.5.5.19976 (for confocal microscopy), NIS-Elements 4.30 (for pig islet and human stainings image acquisition), Xcalibur 2 (for mass spectrometer data collection), QuantStudio Software V1.2.4 (for qPCR data collection and analysis), Abbott glucometer (for adult zebrafish glucose measurements).

Data analysis

Fiji/ImageJ (for image analysis - version 2.0.0-rc-65/1.51w), Excel (version 16.16.27), GraphPad PRISM 8.0&9.0, RStudio 1.4.1717, R package Seurat 3.5.1, R clusterProfiler package (3.10.1), QuantStudio Software V1.2.4 (for qPCR data analysis), MultiQC (version 1.7 - polysome-seq quality check), BBDuk from the BBTools suite (version 36.59 - remove ribosomal RNA reads of the polysome-seq), HISAT2 (version 2.1 - genome alignment of polysome-seq data), RSubread (version 2.6.4 - Summarize reads of the polysome-seq data), R package anota2seq (version 1.14.0 - analysis of polysome-seq data), ClueGO plug-in (version 2.5.8) within CytoScape (version 3.8.2 - gene ontology analysis), Sieve 2.2 was used for chromatographic alignment and peak integration. Morpheus tool from Broad Institute and Metaboanalyst 4.0 was used to analyze the metabolomics data.

For manuscripts utilizing custom algorithms or software that are central to the research but not yet described in published literature, software must be made available to editors and reviewers. We strongly encourage code deposition in a community repository (e.g. GitHub). See the Nature Research guidelines for submitting code & software for further information.

Data

Policy information about availability of data

All manuscripts must include a data availability statement. This statement should provide the following information, where applicable:

- Accession codes, unique identifiers, or web links for publicly available datasets
- A list of figures that have associated raw data
- A description of any restrictions on data availability

The raw data of the metabolomics study is uploaded to Metabolomics Workbench with the study ID ST002119 (http://dx.doi.org/10.21228/M80D9F). The raw data of the polysome profiling is uploaded to GEO with the accession number GSE200477. Expression of mknk2b in zebrafish was assessed using data downloaded from GEO under the accession number GSE106121 and sample number GSM3032164.The rest of the data are included in the current manuscript. The datasets generated during the current study are available from the corresponding author on reasonable request. Source data are provided with this paper.

Field-specific reporting

Please select the one below that is the best fit for your research. If you are not sure, read the appropriate sections before making your selection.

☒ Life sciences ☐ Behavioural & social sciences ☐ Ecological, evolutionary & environmental sciences

For a reference copy of the document with all sections, see nature.com/documents/nr-reporting-summary-flat.pdf

Life sciences study design

All studies must disclose on these points even when the disclosure is negative.

Sample size	No predetermination of sample size was made. We considered a sufficient final sample size when statistical value for group comparison was reached.<br>All in vivo biological experiments in the present manuscript have been repeated at least two times. Each biological replicate included more than seven, embryo or adult, zebrafish. Experiments with pig islets were performed at least two times.<br>All in vitro experiments containing cell lines were repeated at least two times. The kinome screen was performed once. During the course of the study the YCHEMH experiments were repeated multiple times. The polysome-seq data were performed with biological replicates. the human ductal organoid experiments were replicated with tissue from 2 human donors (CID661578 treatment) and 3 human donors (cercosporamide treatment).
Data exclusions	No data were excluded from the final analysis.
Replication	Experiments have been repeated at least two times, with the majority of them having more than three biological replicates. All experiments are reproducible.
Randomization	Zebrafish embryos, pig islet preparations and human duct-derived organoids were randomly assigned to the respective treatment groups, prior to treatment<br>Adult zebrafish used for the experiments were randomly assigned to treatment groups.<br>Regarding cell culture experiments, cells were randomly assigned to treatment groups after plating.
Blinding	Investigators were blinded during the identification of the activity of the analogue CID661578.6 (Supplementary figure 1) and for all the experiments with the mknk2b mutant zebrafish line.<br>In all other experiments investigators were not blinded. When the experimental design required allocation to specific groups or treatments no blinding was required. When different treatment groups were involved the data collection was unbiased and the different groups were blinded for the analysis of these datasets.

Reporting for specific materials, systems and methods

We require information from authors about some types of materials, experimental systems and methods used in many studies. Here, indicate whether each material, system or method listed is relevant to your study. If you are not sure if a list item applies to your research, read the appropriate section before selecting a response.

Materials & experimental systems

n/a	Involved in the study	
☐	☒	Antibodies
☐	☒	Eukaryotic cell lines
☒	☐	Palaeontology and archaeology
☐	☒	Animals and other organisms
☐	☒	Human research participants
☒	☐	Clinical data
☒	☐	Dual use research of concern

Methods

n/a	Involved in the study	
☒	☐	ChIP-seq
☒	☐	Flow cytometry
☒	☐	MRI-based neuroimaging

Antibodies

Antibodies used	For the zebrafish work: #1 - chicken anti-GFP (Aves Labs - GFP-1020, 1:500), #2 - mouse anti-glucagon (1:200; Sigma G6254), #3 - rabbit anti-insulin (1:100; custom made by Cambridge Research Biochemicals), #4 goat anti-tdTomato (1:500; MYBioSource MBS448092).<br><br>For pig islet and human sections stainings: #1 - guinea-pig anti-insulin (1:5; DAKO, code# IR002), #2 - mouse anti-CK7 (3:100; DAKO, clone OV/TL 12/30),#3 - rabbit anti-MNK2 (1:200, Sigma-Aldrich, SAB2101483), #4 - mouse anti-CK19 (1:50, M088801-2, Clone RCK108, Agilent).<br><br>For the cell lines and in vitro reticulocyte western blot experiments: #1 - rabbit anti-EIF4G (1:2000; Cell Signaling Technologies 2498), #2 rabbit anti-EIF4E (1:2000; Cell Signaling Technologies 9742), #3 rabbit anti-MNK2 (Sigma-Aldrich SAB2101483, 1:1000), #4 mouse anti-b-ACTIN (1:5000; Sigma-Aldrich A5441 clone AC-15), #5 anti-rabbit coupled to HRP (1:5000; Thermo Fischer Scientific – 31460), #6 anti-mouse coupled to HRP (1:5000; Jackson ImmunoResearch AB_2307347) #7 mouse anti-FLAG antibody (1:2000, Sigma-Aldrich, F3165), #8 phospho-eIF4E (SER209) (1:1000; Cell Signaling Technologies 9741).
Validation	For the zebrafish stainings: antibody #1 has been cited 1055 times in the literature (e.g https://doi.org/10.15252/embj.201592903), antibody #2 antibody has been cited 264 times in the literature (e.g zebrafish example: doi:10.2337/db16-1587) #3 has been validated using transgenic lines fluorescently labelling the mature beta-cells in zebrafish (e.g see here for validation:DOI: https://doi.org/10.7554/eLife.65758) and antibody #4 has been (e.g DOI: https://doi.org/10.7554/eLife.65758).<br><br>For antibodies used in the pig aggregates and human section stainings, the antibodies were used in previous studies (Hassouna et al; Transplantation; 2018)<br><br>All the antibodies used for the western blot experiments have been widely used and cited in numerous previous studies.#1EIF4G antibody has been validated for western blot with genetic knockdown in mammalian cells (e.g see here as an example https://doi.org/10.3390/ijms20071580). #2 EIF4E antibody has been validated numerous times for western blot with genetic knockdowns in human cell lines in published studies (e.g see here doi: 10.1158/1535-7163.MCT-07-2357). #4 actin antibody has been cited more than 8000 times for western blot applications in mammalian systems (e.g recently here DOI: 10.7554/eLife.74650). #7 anti-FLAG antibody has been cited numerous times for immunoprecipitation experiments (e,g https://doi.org/10.1038/s41467-021-22861-2) and we also validated in Figure 5g where we can see enrichment of the flag tagged protein after immunoprecipitation compared to the input control. #8 p-EIF4E antibody for western blot has been validated by genetic manipulation of MNK kinase (e.g see here: DOI: 10.1126/scisignal.2002466). The #3 MKNK2 antibody was validated by overexpression of MKNK2 protein in COLO320 followed by western blot as part of this study.

Eukaryotic cell lines

Policy information about cell lines

Cell line source(s)	COLO 320HSR and PANC-1 cells were purchased from ATCC.
Authentication	Cells were verified by ATCC using STR profiling and the morphology of the cells was monitored for any changes during experiments.
Mycoplasma contamination	Cell cultures were checked for mycoplasma contamination frequently and no mycoplasma infection was identified during the experiments
Commonly misidentified lines<br>(See ICLAC register)	None of the cell lines used are listed in the ICLAC database

Animals and other organisms

Policy information about studies involving animals; ARRIVE guidelines recommended for reporting animal research

Laboratory animals	For zebrafish experiments either the AB or the TL background strain was used. The transgenic lines used were: Tg(ins:flag-NTR),Tg(ins:GFP), Tg(tp1:H2BmCherry), Tg(tp1:GFP), Tg(gcga:GFP), Tg(ins:CFP-NTR), Tg(ins:Kaede), Tg(ins:H2BGFP), Tg(sst2:dsRed2), Tg(tp1:Cre-ERT2), Tg(-3.5ubb:loxP-EGFP-loxP-mCherry), Tg(tp1:Hsa.MKNK2), Tg(tp1:mknk2b) and mknk2b-4bp deletion mutant line.<br><br>Three ages of zebrafish were used for the experiments: Larvae (up to 6 dpf) - juvenile (1 month old) - adult (4 months old)<br>Sex is not possible to be determined at the larvae and juvenile stage. For the adult experiments the majority of the zebrafish were males. This was done due to availability of males/females in these clutches and not due to a consciously taken decision to include more males.<br><br>Wild type 3 days old neonatal pigs of either sex were sacrificed to obtain the islet cultures for experiments.
Wild animals	The study did not involve wild animals
Field-collected samples	The study did not involve samples collected from the field
Ethics oversight	Work with zebrafish was approved and performed following the guidelines of Stockholms djurförsöksetiska nämnd under an approved ethical permit.<br>Work with neonatal pig islets was performed under the guidelines from Canadian Council on Animal Care.

Note that full information on the approval of the study protocol must also be provided in the manuscript.

Human research participants

Policy information about studies involving human research participants

Population characteristics	General information of the organ donors used for the immunostainings in Fig. g-i: Donor 1: Female, Age:33, Height:1.6(m), Weight: 70(kg), BMI:27.3, Cold Ischemia Time (h): 12. Donor 2: Female, Age:60, Height:1.7(m), Weight: 75.1(kg), BMI:25.9, Cold Ischemia Time (h): 13.5. Donor 3: Male, Age:47, Height:1.61(m), Weight: 106.9(kg), BMI:41.2, Cold Ischemia Time (h): 15.3. No information about chronic medication was available for these donors.<br><br>For the human donors whose pancreas was used to generate the ductal organoids in Fig 6j-k: Donor 1:Male, Age:45 BMI:27,70, Cause of death: Stroke. Donor 2: Male, Age:48, BMI:24,77, Cause of death: Anoxia after cardiac arrest. Donor 3: Male, Age: 58, BMI:26,23, Cause of death: Anoxia after cardiac arrest. No information on chronic medication was available for donors 1&2. Donor 3 had ashtma and was medicated with a bronchodilator when needed, but no other chronic medication was reported.
Recruitment	Human tissues were kindly provided by the organ donors to the Alberta Diabetes Institutes Islet Core and the Hospital de Bellvitge respectively. No preselection of donor/tissue material was done and the selection was based on availability of the human cadaveric material at the time of both studies.
Ethics oversight	1- Ethical approval for the use of human samples for staining was obtained from the University of Alberta's Human Research Ethics Board protocol PRO00001416.<br><br>2- Ethical approval for processing pancreatic samples from deidentified organ donors was granted by the Clinical Research Ethics Committee of Hospital de Bellvitge (PR030/22) for the generation of the human ductal-cerived organoids. Informed consent was obtained for use of the cadaveric material for research purposes.

Note that full information on the approval of the study protocol must also be provided in the manuscript.
