# Supplementary material for: MNK2 deficiency potentiates β-cell regeneration via translational regulation
Source: Nat Chem Biol. 2022 Jun 13;18(9):942–53. doi: 10.1038/s41589-022-01047-x (PMC7613404; doi:10.1038/s41589-022-01047-x)

Figure 5d
(same membrane low exposure)

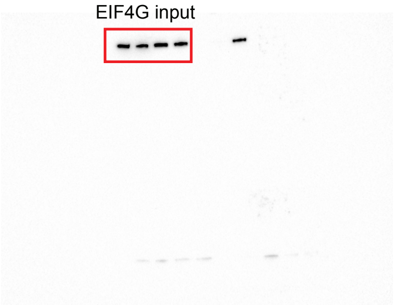

Figure 5d
(same membrane high exposure)

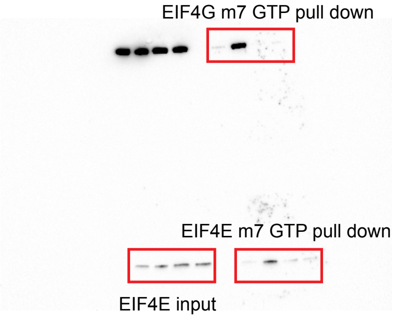

Figure 5d
(colorimetric image for ladder)

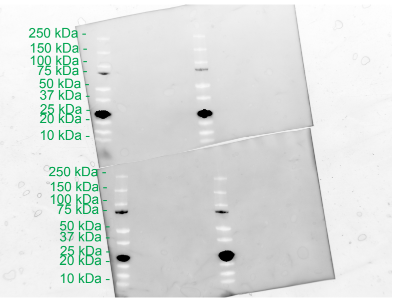

Figure 5e

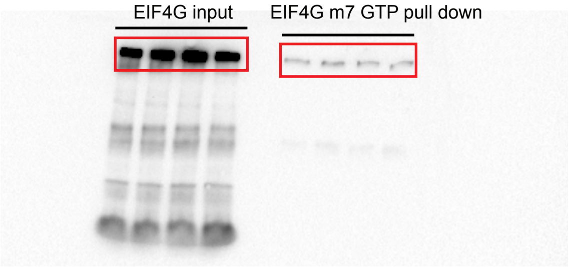

Figure 5e
(colorimetric image for ladder)

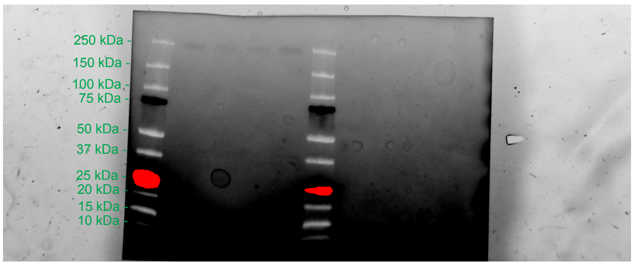

Figure 5e
(same membrane as above, stripped and reblotted for EIF4E)

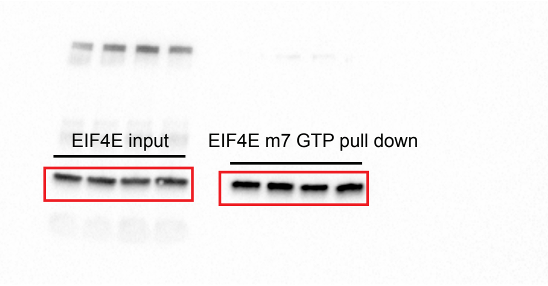

Figure 5e
(colorimetric image for ladder)

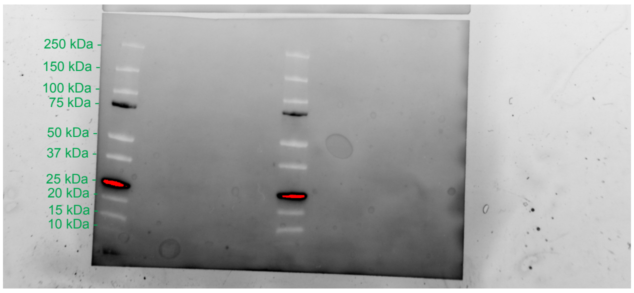

Figure 5f
(1- flipped horizontally in the main figure
&high exposure)

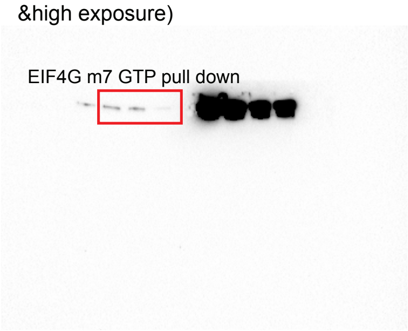

Figure 5f
(1- flipped horizontally in the main figure
&low exposure)

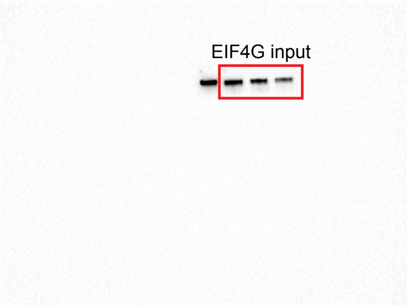

Figure 5f
(colorimetric image for ladder)

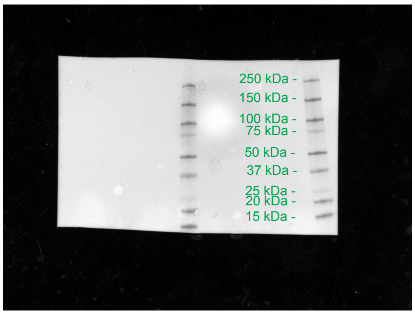

Figure 5f
(1- flipped horizontally in the main figure
2- same mebrane as above, stripped
and reblotted for EIF4E)

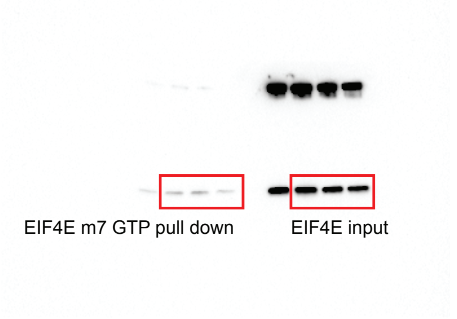

Figure 5f
(colorimetric image for ladder)

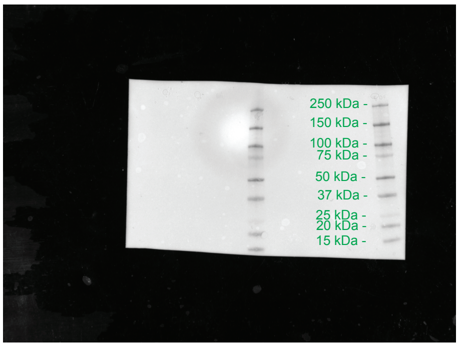

Figure 5g
(1- flipped horizontally in the main figure
and low exposure)

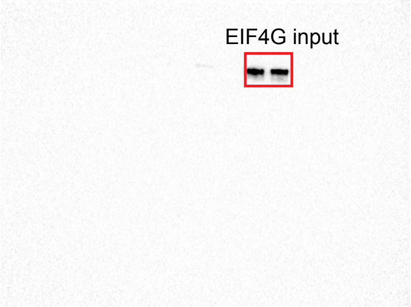

Figure 5g
(1- flipped horizontally in the main figure
and high exposure)

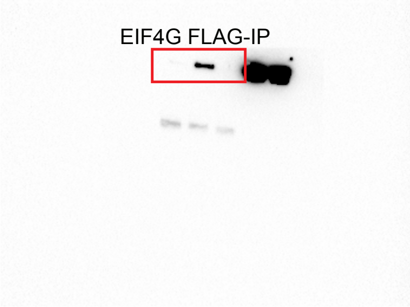

Figure 5g
(colorimetric image for ladder)

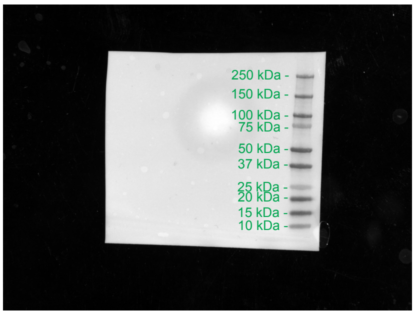

Figure 5g
(1- flipped horizontally in the main figure
2- same mebrane as above, stripped
and reblotted for MNK2
3- changed contrast on the raw image
to make the main figure brighter for printing
purposes)

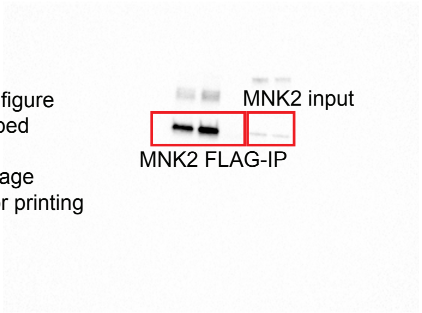

Figure 5g
(colorimetric image for ladder)

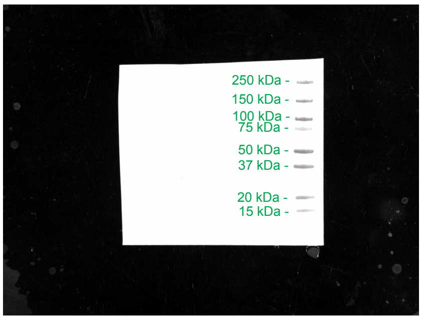

Figure 5h
(flipped horizontally and slightly rotated in the main figure)

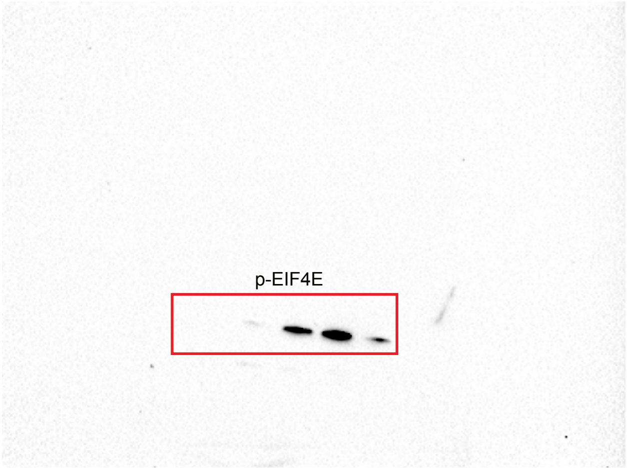

Figure 5h
(colorimetric image for ladder)

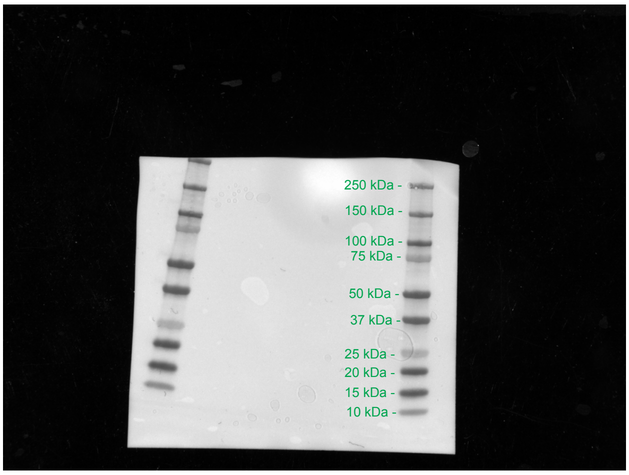

Figure 5h
(1 - flipped horizontally and slightly rotated in the main figure
2- same mebrane as above, stripped and reblotted for total EIF4E)

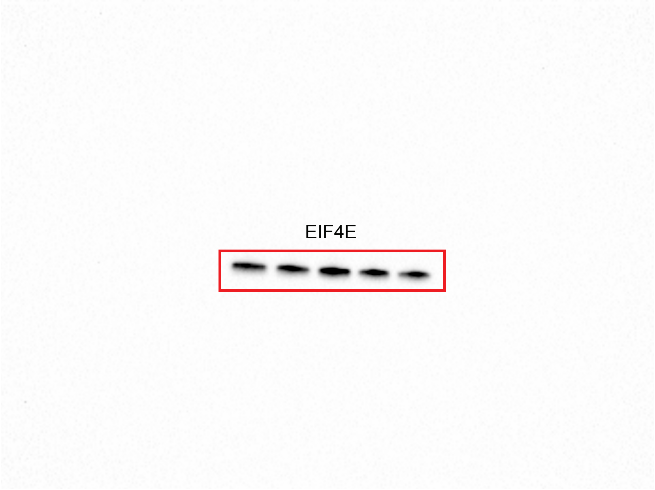

Figure 5h
(colorimetric image for ladder)

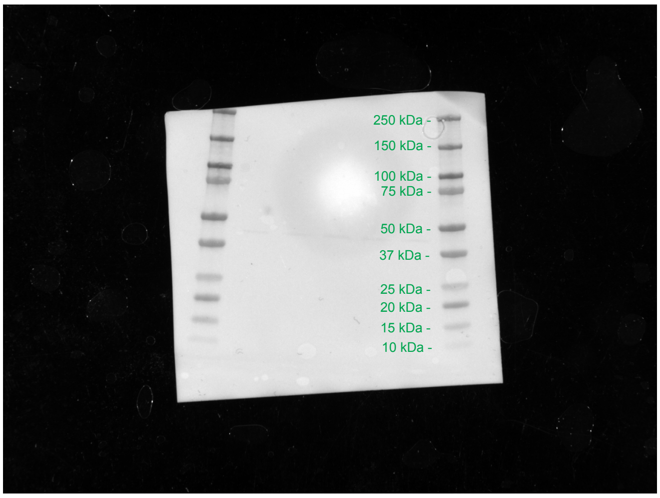

Figure 5h
(1 - flipped horizontally and slightly rotated in the main figure
2- same mebrane as above, stripped and reblotted for total ACTIN)

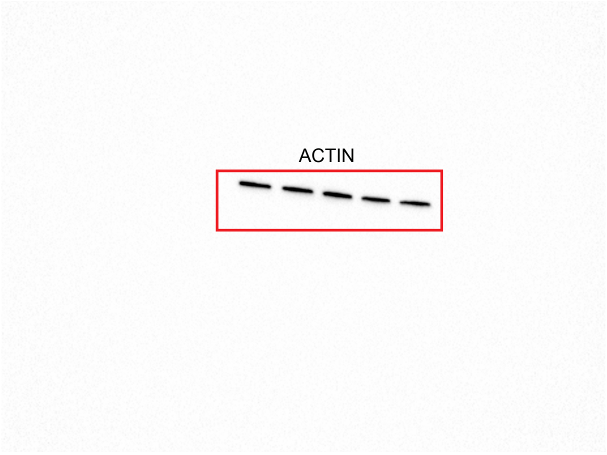

Figure 5h
(colorimetric image for ladder)

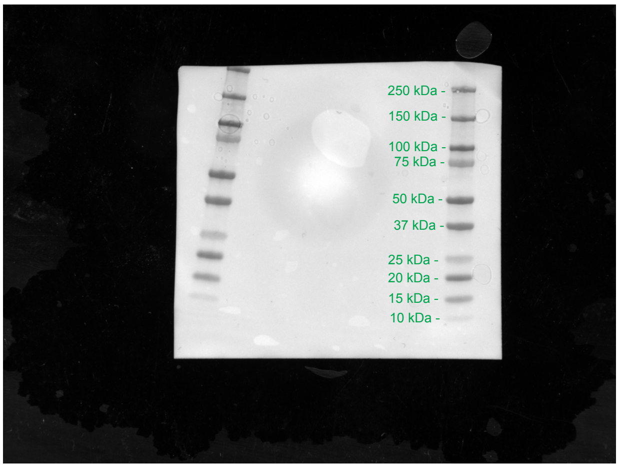

Supplement: Source Data Fig. 5 — Uncropped raw western blot images for Fig. 5. [file 41589_2022_1047_MOESM10_ESM.pdf]
