# Supplementary material for: MNK2 deficiency potentiates β-cell regeneration via translational regulation
Source: Nat Chem Biol. 2022 Jun 13;18(9):942–53. doi: 10.1038/s41589-022-01047-x (PMC7613404; doi:10.1038/s41589-022-01047-x)

Supplementary Fig 7k

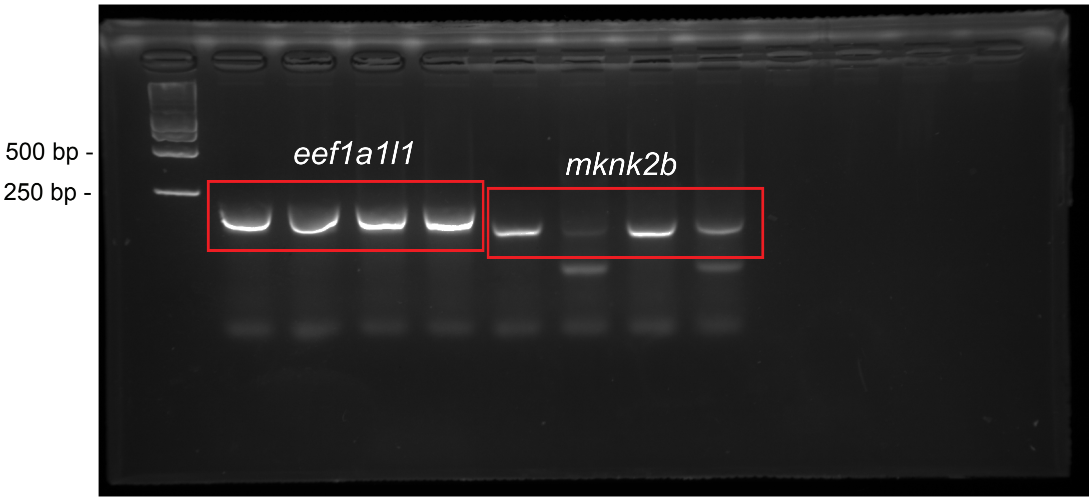

Supplementary Fig 7l

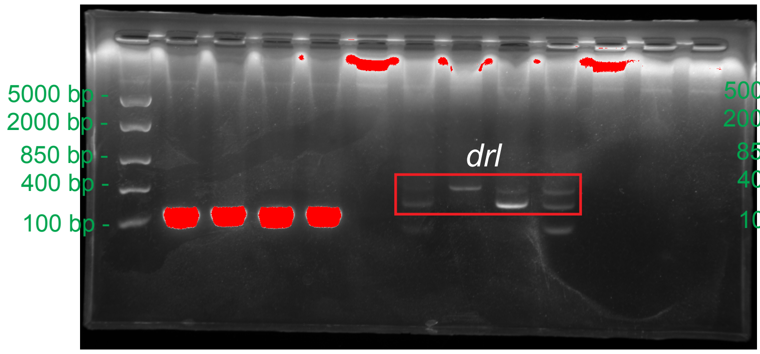

Supplementary Fig 7l (same gel different exposure)

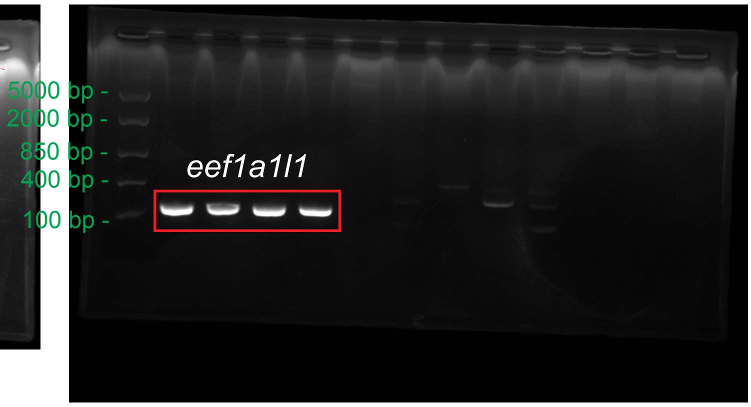

Supplementary Fig 7m

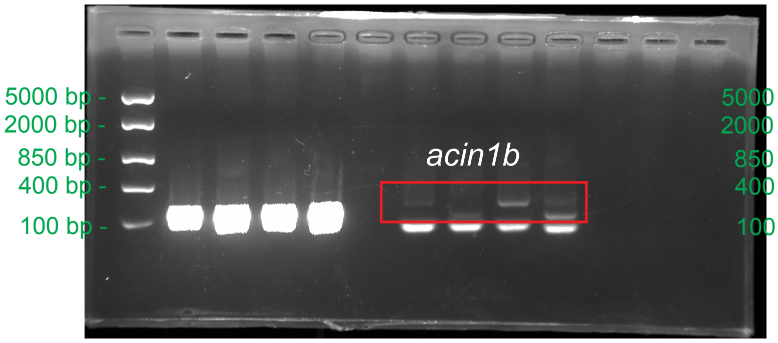

Supplementary Fig 7m (same gel different exposure)

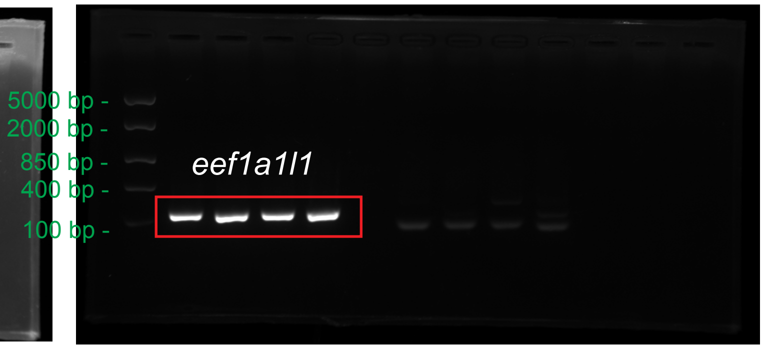

Supplement: Source Data Extended Data Fig. 7 — Uncropped raw agarose gel images for Extended Data Fig. 7. [file 41589_2022_1047_MOESM18_ESM.pdf]
